# Baicalein Prevents Fructose-Induced Hepatic Steatosis in Rats: In the Regulation of Fatty Acid De Novo Synthesis, Fatty Acid Elongation and Fatty Acid Oxidation

**DOI:** 10.3389/fphar.2022.917329

**Published:** 2022-06-30

**Authors:** Pan Li, Ruoyu Zhang, Meng Wang, Yuwei Chen, Zhiwei Chen, Xiumei Ke, Ling Zuo, Jianwei Wang

**Affiliations:** ^1^ Chongqing Key Laboratory of Traditional Chinese Medicine for Prevention and Cure of Metabolic Diseases, College of Traditional Chinese Medicine, Chongqing Medical University, Chongqing, China; ^2^ The Pharmacy Department, the Second People’s Hospital of Jiulongpo District, Chongqing, China

**Keywords:** non-alcoholic fatty liver, hepatic steatosis, Baicalein, fatty acid synthesis, fatty acid elongation, fatty acid oxidation

## Abstract

Non-alcoholic fatty liver disease (NAFLD), ranging from simple steatosis to non-alcoholic steatohepatitis (NASH), hepatic fibrosis and even hepatocellular carcinoma, is a liver disease worldwide without approved therapeutic drugs. Baicalein (BAL), a flavonoid compound extracted from the Traditional Chinese Medicine (TCM) Scutellariae Radix (*Scutellaria baicalensis* Georgi.), has been used in TCM clinical practice for thousands of years to treat liver diseases due to its “hepatoprotective effect”. However, the underlying liver-protecting mechanisms remain largely unknown. Here, we found that oral administration of BAL significantly decreased excess serum levels of triglyceride (TG), low-density lipoprotein cholesterol (LDL-C), aspartate aminotransferase (AST) as well as hepatic TG in fructose-fed rats. Attenuation of the increased vacuolization and Oil Red O staining area was evident on hepatic histological examination in BAL-treated rats. Mechanistically, results of RNA-sequencing, western-blot, real-time quantitative PCR (RT-qPCR) and hepatic metabolomics analyses indicated that BAL decreased fructose-induced excessive nuclear expressions of mature sterol regulatory element-binding protein 1c (mSREBP1c) and carbohydrate response element-binding protein (ChREBP), which led to the decline of lipogenic molecules [including fatty acid synthase (FASN), stearoyl-CoA desaturase 1 (SCD1), elongation of very long chain fatty acids 6 (ELOVL6), acetyl-CoA carboxylase (ACC)], accompanying with the alternation of hepatic fatty acids composition. Meanwhile, BAL enhanced fatty acid oxidation by activating AMPK/PGC1α signaling axis and PPARα signal pathway, which elicited high expression of carnitine palmitoyl transferase 1α (CPT1α) and Acyl-CoA oxidase 1 (ACO1) in livers of fructose-fed rats, respectively. BAL ameliorated fructose-induced hepatic steatosis, which is associated with regulating fatty acid synthesis, elongation and oxidation.

## Graphical Abstract

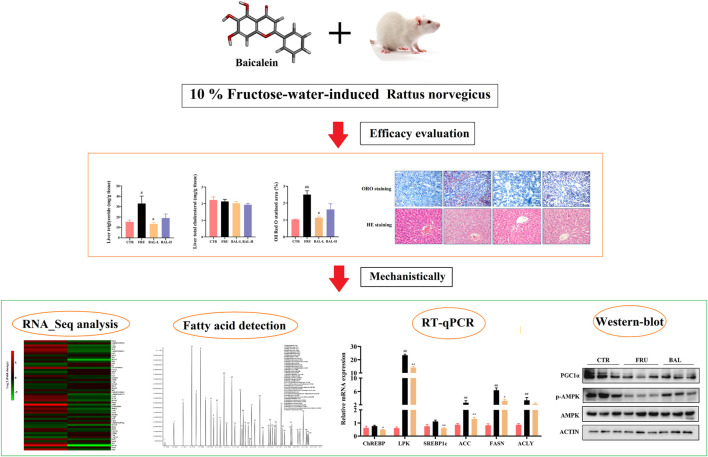

## Introduction

Non-alcoholic fatty liver disease (NAFLD) is the most common chronic liver metabolic disease, with an estimated prevalence of up to 25.2% worldwide (Younossi et al., 2016). The NAFLD spectrum of disease states can progress from simple hepatic steatosis to non-alcoholic steatohepatitis (NASH) followed by the progression into fibrosis, cirrhosis and even hepatocellular carcinoma. More importantly, it is an important pathogenic risk factor for diabetes, cardiovascular diseases and tumors (Eslam et al., 2020; Huang et al., 2021; Sun et al., 2021). At present, no treatment strategy has been approved by Food and Drug Administration (FDA) for NAFLD, and hence there is an urgent need for the developmental research of anti-NAFLD drug.

NAFLD is characterized by excessive lipid accumulation in the liver, and fatty acids (FAs) are the simplest lipids, serving as the basic components and synthetic materials for more complex lipids (including triglycerides, phospholipids, and sphingolipids) (Wang et al., 2011). Chronic and/or excessive consumption of carbohydrates and saturated FAs has a close relationship with *de novo lipogenesis* (DNL) and FAs metabolism *in vivo* (Matsuzaka et al., 2020). Elongation and desaturation of long-chain FAs are critical steps in DNL, and the length and saturation of long-chain FAs, which are regulated by the ELOVL fatty acid elongase family and stearoyl-CoA desaturase (SCD) family of proteins, play the significant roles in the developments of FAs function and metabolism (Guillou et al., 2010). Proper elongation and desaturation of FAs are essential for the maintenance of lipid homeostasis, and conversely, disruption of this balance results in metabolic disorders such as NAFLD and diabetes (Matsuzaka, 2021).

Baicalein (BAL, NCBI CID: 5281605), a major flavonoid compound isolated from the roots of the Traditional Chinese Medicine (TCM) *Scutellaria baicalensis* Georgi (Labiatae), has been used to treat liver disease for thousands of years in TCM clinical practice (Li-Weber, 2009; Dang et al., 2019). Previous studies indicated that BAL can protect the liver from high-fat diet (HFD)-induced hepatic steatosis, lipopolysaccharides (LPS)/d-galactosamine (D-gal) and high-cholesterol diet (HCD)-induced liver injury by maintaining V-ATPase assembly, antioxidant stress and anti-inflammatory effect, respectively (Alsaad et al., 2020; Zhu et al., 2020; Xiao et al., 2021). Importantly, it was observed that high oral doses of BAL (100–2,800 mg) were safe and well-tolerated in healthy subjects, implying that BAL is a promising natural product for clinical use (Li et al., 2014). However, the mechanisms underlying the effect of BAL on hepatic steatosis are not completely known and clear, particularly in the regulation of FAs composition and metabolism by BAL treatment in high carbohydrate-induced hepatic steatosis.

In this study, we established a fructose-induced hepatic steatosis rat model to investigate the pharmacological activity of BAL, and then RNA-Sequencing analysis, hepatic metabolomics, RT-qPCR and Western-blot analysis were performed to explain its underlying molecular mechanisms.

## Materials and Methods

### Agents

Baicalein (purity ≥98%) was purchased from Shanghai Macklin Biochemical Co., Ltd (Shanghai, China). Gum Arabic were obtained from Wako (Osaka, Japan). The antibody against FASN (CAT: 3180s), ACC (CAT: 3676s), AMPKα (CAT: 3732s), p-AMPKα (Thr 172, CAT: 2535s) and ACTIN (CAT: 970s) were purchased from Cell Signaling Technology (Beverly, MA). And mouse monoclonal antibody CPT1α (Cat: ab128568) was purchased from Abcam (Cambridge, England). The antibodies for ELOVL6 (CAT: 21160-1-AP) and Lamin B1 (CAT: 12987-1-AP) were from Proteintech (Rosemont, United States), Antibody against ChREBP (CAT: TA309750) was obtained from OriGene Technologies (Rockville, MD, United States), and Antibody against SREBP1c (CAT: sc-13551), PPARα (CAT: sc-398394) and SCD1 (CAT: sc-81776) were purchased from Santa Cruz Biotechnology (Santa Cruz, CA, United States).

### Animal Study

Male, Sprague-Dawley rats, weighing at 210–230 g, were acclimated to 12 h dark-light cycles in a temperature-controlled facility (24 ± 2°C, 55 ± 5% relative humidity) at the Laboratory Animal Centre of Chongqing Medical University, China. After 1 week of adaptable feeding, rats were randomly divided into four groups (*n* = 8 per group): 1) water control group, with free access to water [CTR: 5% gum arabic solution, intragastric administration once daily (i.g.)]; 2) fructose control group, with free access to 10% fructose solution (w/v, preparation every day, FRU group: 5% gum arabic solution, i. g.); 3) fructose-BAL low dose group (BAL was suspended in 5% gum arabic solutio, BAL-L group: 25 mg/kg, i. g.), and 4) fructose-BAL high dose group (BAL-H: 100 mg/kg, i. g.). Meanwhile, to account for variations in fructose intake, the fructose concentration was adjusted once every 3 days depending on the fructose consumption in the fructose control over the previous 3 days as we previously described (Liu et al., 2013). The experiments were lasted for 5 weeks, and then the blood samples were collected from the abdominal aorta after narcotizing by isoflurane (for 10 h-fasting). Rats were sacrificed by head dislocation. Tissue samples were isolated and immediately stored at −80°C for further studies.

### Determination of Serum Biochemical Index and Hepatic Triglyceride (TG) and Total Cholesterol (TC) Levels

Serum triglyceride (TG), total cholesterol (TC), high-density lipoprotein cholesterol (HDL-C), low-density lipoprotein cholesterol (LDL-C), alanine transaminase (ALT), and aspartate aminotransferase (AST) were enzymatically determined using biochemical test kits (Nanjing Jiancheng Bioengineering Research Institute Co., Ltd., Nanjing, China) strictly according to the instructions contained in the kits’ instruction manuals.

Moreover, the liver TG and TC levels were also detected in rats. Briefly, raw liver tissues were weighed and recorded accurately, the tissues were extracted by isopropyl alcohol with a ratio of 50:1 (50 mg tissue dissolved in 1 ml isopropyl alcohol). Then added the grinding beads for fully homogenizing the tissues and put the homogenate on the Four-Dimensional Rotating Mixer (Beyotime, Shanghai, China) at 4°C overnight. Finally, the homogenate of liver tissues was centrifugated at 3,000 rpm for 10 min and the supernatant were detected TG and TC using the commercial test kits (Nanjing Jiancheng Bioengineering Research Institute Co., Ltd., Nanjing, China) according to the manufacturer’s instructions. The levels of TG and TC in rat liver were expressed as the ratios of TG (or TC) value and liver tissue weight.

### Histological Examination

Hematoxylin and Eosin (H&E) staining was performed to examine the pathology of rat liver. Referred to previous study (Li W. et al., 2021), briefly, fresh rat liver tissues were fixed with 4% paraformaldehyde, dehydrated and embedded in paraffin after routine dehydration. Subsequently, sliced into 4-μm sections with Automatic Slicer (Shanghai Leica Instrument Co. Ltd., Shanghai, China), followed by dewaxing and H&E staining. Finally, the pathological morphological changes of liver tissues of rats were observed using an optical microscope.

In addition, Oil red O (ORO) Staining was also used to investigate the hepatic lipid accumulation in fructose-fed rats. As previous descriptions with a little adjustment (Li L. et al., 2021), liver tissues were fixed with 4% paraformaldehyde and put the tissues in 15–30% sucrose solution for dehydrating twice at 4°C. After that, embedding agent were used to embed. Then the tissues were cut into 8-μm sections using Automatic Slicer and stained with Oil red O (ORO). Finally, hematoxylin was used for counterstaining followed by washing sections with pure water three times and sealed the slices with glycerin gelatin. Observed with microscope inspection to examine hepatic lipid droplets, the lipid droplets are red while the nuclei are blue. And the ratio of the ORO-stained area to the total tissue area was calculated (%) using ImageJ (V: 1.8.0).

### Identification of Differently Expressed Genes by RNA_Sequence Analysis

Total RNA was extracted from rat livers (4 samples from each treatment group, including the CTR group, fructose-fed group, and BAL treatment group) using TRIzol^®^ Reagent according to the manufacturer’s instructions (Invitrogen) and genomic DNA was removed using DNase I (TaKara). The quality of the RNA was determined using the 2,100 Bioanalyser (Agilent) and quantified using the ND-2000 (NanoDrop Technologies). The RNA-seq transcriptome library was prepared with 1 μg of total RNA using the TruSeqTM RNA sample preparation Kit from Illumina (San Diego, CA). Briefly, messenger RNA was isolated using the polyA selection method by oligo (dT) beads and then fragmented using fragmentation buffer. Double-stranded cDNA was synthesized using a SuperScript double-stranded cDNA synthesis kit (Invitrogen, CA) and random hexamer primers (Illumina). Following that, the synthesized cDNA was subjected to end-repair, phosphorylation, and “A” base addition in accordance with the Illumin library construction protocol. Libraries were size selected for 300 bp cDNA target fragments on 2% Low Range Ultra Agarose, and then PCR amplified for 15 PCR cycles using Phusion DNA polymerase (NEB). After quantification with TBS380, the paired-end RNA-seq sequencing library was sequenced using the Illumina HiSeq xten/NovaSeq 6,000 sequencer (2 × 150 bp read length).

The raw paired-end reads were trimmed and quality controlled using SeqPrep (https://github.com/jstjohn/SeqPrep) and Sickle (https://github.com/najoshi/sickle) with default parameters. The clean reads were separately aligned to a reference genome in orientation mode using the HISAT2 (http://ccb.jhu.edu/software/hisat2/index.shtml) software. The mapped reads of each sample were assembled using StringTie (https://ccb.jhu.edu/software/stringtie/index.shtml?t=example). The expression was calculated based on the transcripts per million reads (TPM) to identify the differentially expressed genes (DEGs). The DEGs were identified using fold change and the *p-value* was calculated using *t*-test analysis, with a *fold change ≥ 1.5* and a *p-value ≤ 0.05* used as the threshold for up-and downregulated genes. Additionally, the functional enrichment of differentially expressed genes (shown in Supplementary Table S1 and Supplementary Table S2) was determined using the gene ontology (GO) and Kyoto Encyclopedia of Genes and Genomes (KEGG) annotation system. Finally, the genes and enrichment pathways of interest were selected for further investigation.

### Analysis of Hepatic Fatty Acids

Standard preparation: a total of 40 fatty acid methyl esters mixed standard solution were diluted into 0.5 mg/L, 1 mg/L, 5 mg/L, 10 mg/L, 25 mg/L, 50 mg/L, 100 mg/L, 250 mg/L, 500 mg/L and 1,000 mg/L of mixed standard sample, in which the concentration of the mixed standard sample is the total concentration of each component. And there were two gradients’ ratios (2% for 30 components and 4% for 10 30 components) of each component concentrated to the total in the 40 fatty acid methyl esters mixed standard solution. Then took 500 μl mixed standard, added 25 μl methyl n-octanoate with a concentration of 500 ppm (as the internal standard), after mixing, 1 μl mixed samples were injected and tested by Gas Chromatography-Mass Spectrometer (GC-MS), with split injection and the split ratio was 10:1.

Metabolite extraction: 30 mg liver tissues were exacted with 1 ml chloroform methanol solution and ultrasonic crushed for 30 min, then absorbed the supernatant and added 2 ml 1% sulfuric acid methanol solution at 80°C to methylate for 30 min. Exacted with 1 ml n -hexane following by washing with 5 ml pure water. After that, collected 500 μL supernatant and added 25 μl methyl nonadecanoate as the internal standard. After mixing, 1 μl mixed samples were injected and tested by GC-MS, with split injection and the split ratio was 10:1.

GC-MS analysis: briefly, the hepatic fatty acids were isolated and collected using an Agilent DB-WAX capillary column (30 m × 0.25 mm ID × 0.25 μm, Agilent, United States). Temperature program: Initial temperature was set to 50°C for 30 min, and then increased to 220°C at a rate of 10°C/min for 5 min. Helium was used as the carrier gas, with a flow rate of 1.0 ml/min. To test and determine the stability and repeatability of the system, quality control (QC) samples were interspersed and determined in the samples. Meanwhile, mass spectrometry was performed using an Agilent 7,890/5975c GC-MS (Agilent Technologies, United States), and the mass spectrum conditions were as follows. Inlet temperature: 280°C; ion source temperature: 230°C; transmission line temperature: 250°C; with electron bombardment ionization (EI) source, SIM scanning mode, and the electron energy was 70ev. Finally, the chromatographic peak area and retention time were extracted using the MSD Chemstation software (Agilent, United States), and hepatic fatty acid content was calculated using the drawn standard curve. The total ions chromatogram (TIC) and the detection standard curve for each fatty acid are shown in Supplementary Figure S1 and Supplementary Table S3, respectively.

### RT-qPCR Analysis

Total RNA was extracted from 50 mg liver tissues using the TRIzol^®^ reagent (Invitrogen, Chicago, United States). The absorbance ratio of 1.8–2.0 at 260/280 nm was determined using the NanoDrop 2000 spectrophotometer (Thermo Fisher Scientific, Wilmington, DE, United States), while the RNA purity was calculated simultaneously. RNA was reversed transcribed to cDNA using the TaqMan Reverse Transcription Reagents (Applied Biosystems, Foster City, United States). Following that, RT-qPCR was performed using the SYBR green kit (Kapa Biosystems, Wilmington, MA, United States) according to the manufacturer’s instructions. The primer sequences (Tsingke Biotechnology Co., Ltd, Shanghai, China) were shown in Table 1. Finally, GAPDH (internal control gene) mRNA was used to normalize the relative mRNA expression, and the results were reported using the 2^−ΔΔCt^ method.

**TABLE 1:** primers for RT-qPCR.

Gene	Species	Forward Primer	Reverse Primer
SCD1	Rat	CAG​TTC​CTA​CAC​GAC​CAC​CAC​TA	GGA​CGG​ATG​TCT​TCT​TCC​AGA​T
ACACA	Rat	TTC​CCA​TCC​GCC​TCT​TCC​TGA​C	TGC​TTG​TCT​CCA​TAC​GCC​TGA​AAC
ELOVL6	Rat	CTC​AGC​AAA​GCA​CCC​GAA​CTA​GG	CCA​AGA​GTA​CAG​GAG​CAC​AGT​GAT​G
PPARα	Rat	GTC​ATC​ACA​GAC​ACC​CTC​TCC​C	TGT​CCC​CAC​ATA​TTC​GAC​ACT​C
NR1H3	Rat	GAG​ACA​TCG​CGG​AGG​TAC​AA	TAA​TGA​ACT​CCA​CCT​GCA​GCC
FASN	Rat	ACC​TCA​TCA​CTA​GAA​GCC​ACC​AG	GTG​GTA​CTT​GGC​CTT​GGG​TTT​A
SREBP1c	Rat	CCT​GCT​TCT​CTG​GGC​TCC​TCT​C	GCA​CGG​ACG​GGT​ACA​TCT​TTA​CAG
ChREBP	Rat	GAA​GAC​CCA​AAG​ACC​AAG​ATG​C	TCT​GAC​AAC​AAA​GCA​GGA​GGT​G
AMPK	Rat	CTC​AAC​CGT​TCT​ATT​GCC​ACT​CT	AGG​AAA​GAG​GTA​ACT​GGG​CAA​AT
CPT1α	Rat	CAG​GAG​AGT​GCC​AGG​AGG​TCA​TAG	TGC​CGA​AAG​AGT​CAA​ATG​GGA​AGG
DGAT2	Rat	CCT​GGC​AAG​AAC​GCA​GTC​AC	GAG​CCC​TCC​TCA​AAG​ATC​ACC
MGAT2	Rat	GCG​ACA​AAG​GAA​GAA​CGA​CG	TTA​GAA​ACC​CTG​CGG​ATG​CC
ACO1	Rat	TTC​AAG​ACA​AAG​CCG​TCC​AA	TGC​TCC​CCT​CAA​GAA​AGT​CC
ACLY	Rat	GTT​GCG​TTT​GTG​GAC​ATG​CT	CCC​GAT​GAA​GCC​CAT​ACT​CC
LPK	Rat	GAC​CCG​AAG​TTC​CAG​ACA​AGG	ATG​AGC​CCG​TCG​TCA​ATG​TAG
GAPDH	Rat	GAA​GGT​CGG​TGT​GAA​CGG​AT	CCC​ATT​TGA​TGT​TAG​CGG​GAT

### Western Blotting Analysis

Briefly, 80 mg liver tissues were lysed using RIPA lysis buffer containing 1 mM protease inhibitor cocktail on ice for 30 min to extract total protein, and total protein concentrations were determined using a BCA kit (Beijing Solarbio Science and Technology Co., Ltd, Beijing, China). The denatured proteins were resolved using 8–12% SDS-PAGE (Bio-Rad Laboratories) and transferred to PVDF membranes. After blocking with 5% non-fat dry milk, the membranes were incubated with primary antibodies overnight at 4°C. The following day, after washing the membranes with TBST (0.1% Tween-20), they were incubated with an HRP-conjugated secondary antibody (Beijing Biosynthesis Biotechnology, Beijing, China) for 1 h at room temperature. After that, the membranes were washed three times with TBST. Finally, chemiluminescence was used to detect the protein expressions (Bio-Rad Laboratories, Inc, California, United States).

### Statistical Analysis

The values are expressed as mean ± SEM from at least six samples used for analysis throughout the experiment. Comparisons were performed using one-way ANOVA for multiple groups or the Student’s t-test for two groups (GraphPad Prism 8.0, San Diego, CA, United States). *p < 0.05* was considered to be statistically significant.

## Result

### BAL Ameliorates Fructose-Induced Metabolic Syndrome in Rats

Fructose-treated rats (FUR) showed a reduction in chow-intake and higher liver/body ratio compared with the rats without fructose-fed (CTR). These parameters (including chow-intake, fructose-intake, body weight and the liver/body ratio) were not altered with the different doses treatment of BAL in fructose-fed rats (Supplementary Figures S2A-D).

**FIGURE 1:**
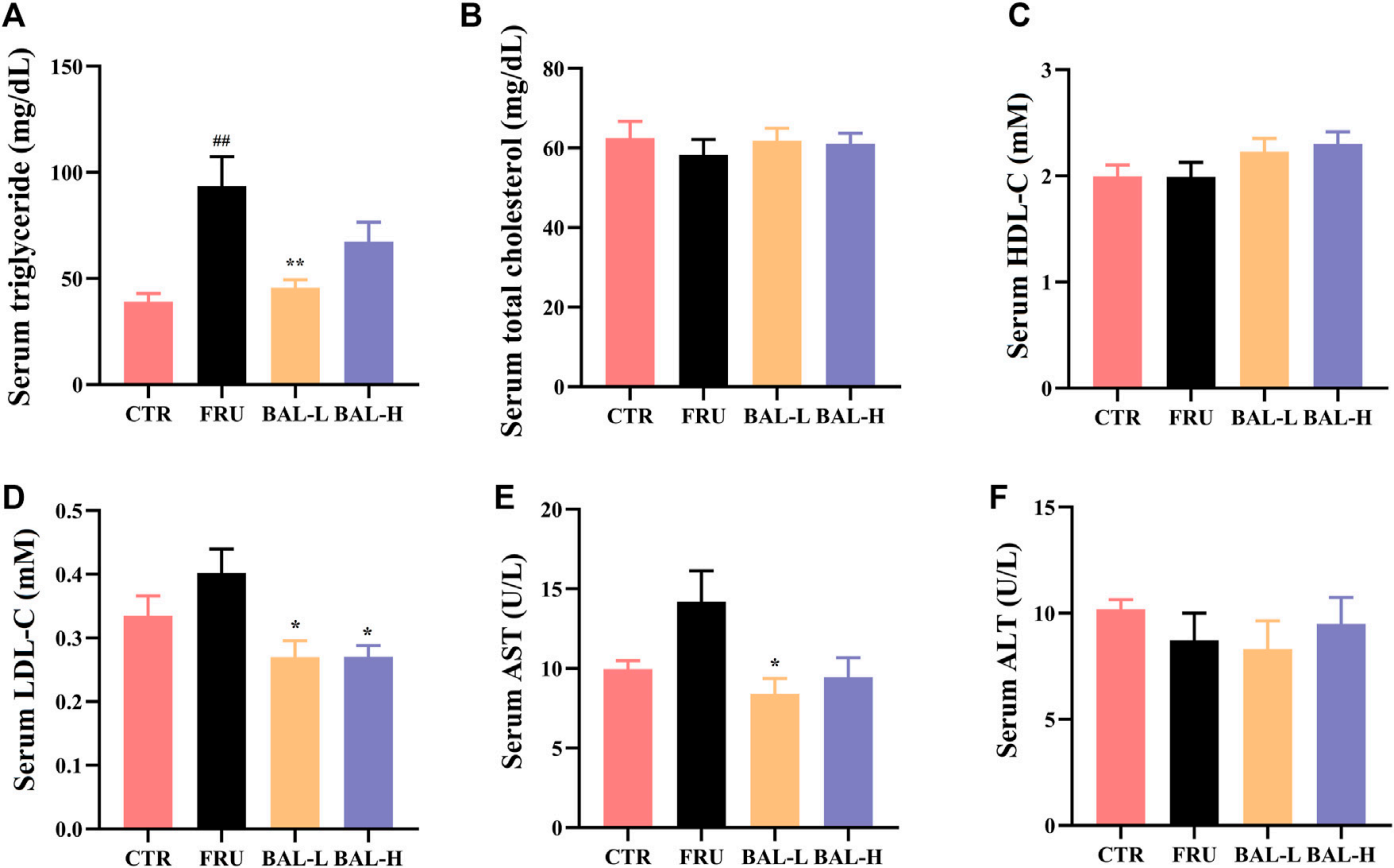
The serum biochemical indexes of rats during BAL treatment. **(A)** Serum TG. **(B)** Total TC in serum. **(C)** HDL-C in serum. **(D)** Serum LDL-C. **(E)** Serum level of AST. **(F)** ALT level in serum. Data were represented as Mean ± SEM (n ≥ 6/group), ^*##*^ *p < 0.01*, ^#^ *p < 0.05*, compared with CTR; **p < 0.01, *p < 0.05, compared to FRU.

Analysis of serum lipid profiles in fructose-fed rats showed that fructose-fed significantly increased serum TG in rats, which was decreased following BAL-L (25 mg/kg) treatment, and BAL-H (100 mg/kg) treatment rats showed a downward trend (*p = 0.18*) of serum TG (Figure 1A). Of note, it was also observed that fructose drinking did not change the serum TC, HDL-C, LDL-C, ALT and AST obviously, however, BAL treatment reduced the serum levels of LDL-C (both BAL-L and BAL-H treatment) and AST (BAL-L treatment) after 5 weeks of administration (Figures 1B–F). These results indicated that BAL ameliorates the metabolic disorders in fructose-fed rats.

### BAL Alleviates Hepatic Lipid Accumulation in Fructose-Induced Rats

**FIGURE 2:**
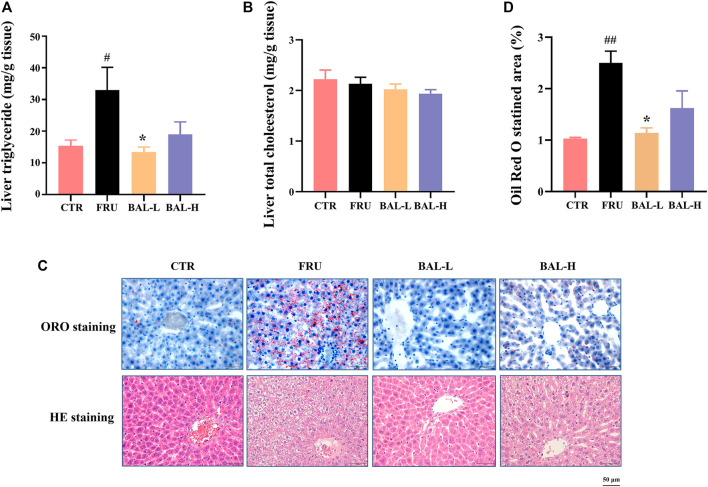
Effect of BAL treatment on hepatic lipid accumulation in fructose-fed rats. **(A)** TG in liver. **(B)** Hepatic content of TC. **(C)** Pathological morphology of liver by ORO staining and HE staining. **(D)** Relative quantification of ORO staining area by figure J software. Data were represented as Mean ± SEM (n = 6/group), ^*##*^ *p < 0.01*, ^*#*^ *p < 0.05*, compared with CTR; **p < 0.01, *p < 0.05, compared to FRU.

To investigate the effect of BAL on hepatic steatosis caused by excessive fructose consumption in rats, we examined the hepatic lipid profiles. As expected, fructose significantly increased the hepatic TG content in rats compared to CTR group, BAL-L treatment reversed the fructose-induced increase in TG content whereas BAL-H treatment appeared the trend of decrease (*p = 0.13*). Meanwhile, we could not find the differences in TC levels among these four groups (Figures 2A,B). Consistently, results of hepatic ORO and HE staining revealed that BAL-L treatment alleviated the increase in hepatic lipid droplet accumulation caused by fructose (Figures 2C,D), demonstrating that BAL showed an evident protect against fructose-induced hepatic steatosis.

### BAL Alters the Gene Expression Patterns in the Liver

**FIGURE 3:**
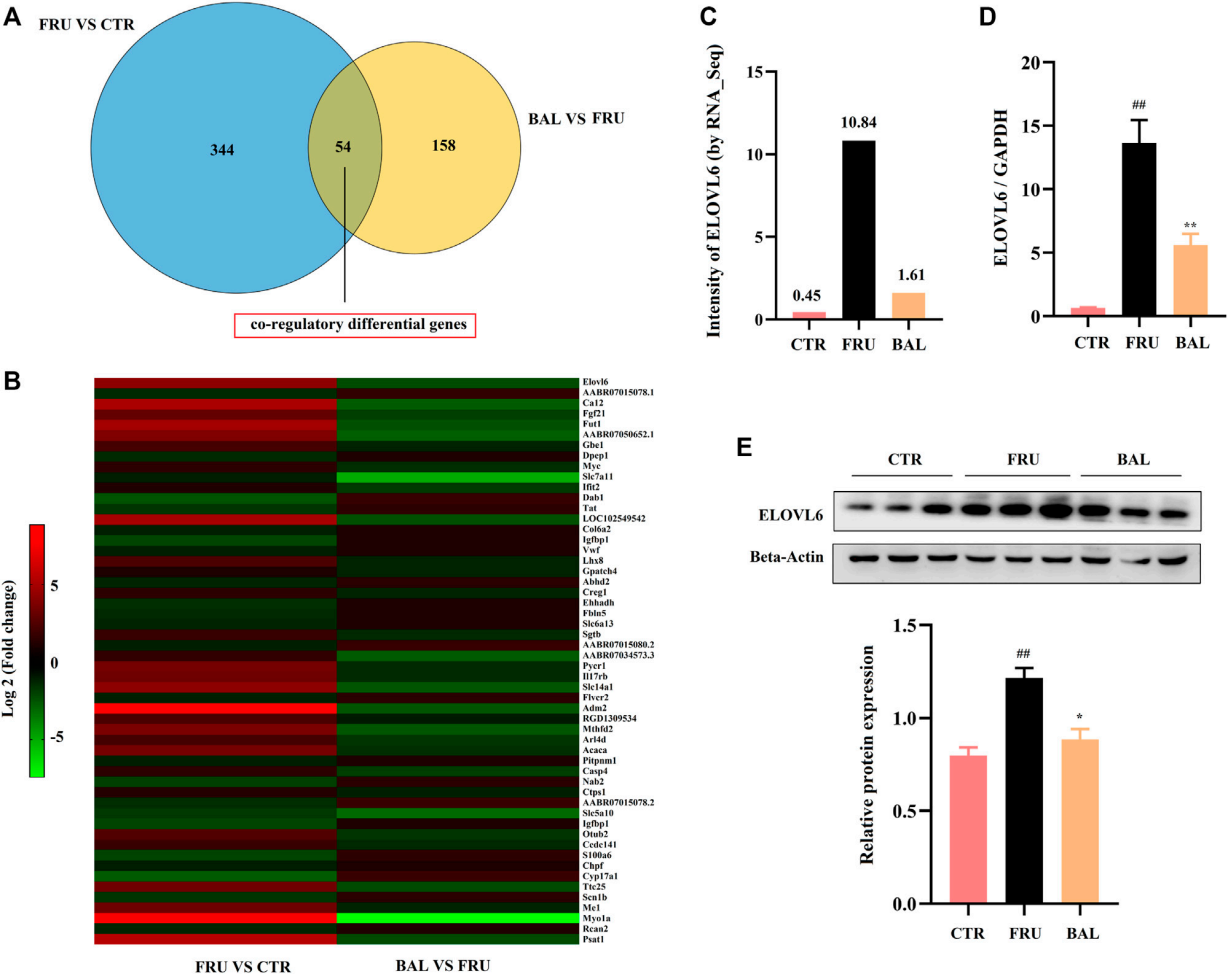
RNA-sequencing analysis and verification. **(A)** Venn analysis of differentially expressed genes. **(B)** Heat-map of co-regulatory differentially genes. **(C)** Intensity of ELOVL6 in RNA-Sequencing analysis. **(D)** RT-qPCR analysis of ELOVL6 in rat liver. **(E)** ELOVL6 protein expression in liver by western-blot. Data were represented as Mean ± SEM (*n* = 6/group), ^*##*^ *p < 0.01*, ^*#*^ *p < 0.05*, compared with CTR; **p < 0.01, *p < 0.05, compared to FRU.

To investigate how BAL treatment affected hepatic steatosis in fructose-fed rats, RNA-Sequencing analysis was carried out to identify the differentially expressed genes by BAL treatment (this was done using the BAL-L treatment). Results revealed that a total of 54 genes were significantly and differentially expressed following by fructose and BAL treatment among the three groups (named co-regulatory genes, Figure 3A). Cluster analysis of the co-regulatory genes was shown in Figure 3B. Meanwhile, analyzing the enrichment pathways of these genes with DAVID Bioinformatics Resources 6.8 (a functional annotation tool online, https://david.ncifcrf.gov/home.jsp) (Table 2).

**TABLE 2:** KEGG pathway enrichment analysis of co-regulatory genes (by Davide, Dec. 20th, 2021).

NO	Term	Count	Gene
1	Metabolic pathways	12.00	Gbe1, Tat, Fut1, Pycr1, Psat1, Me1, Cyp17a1, Acaca, Ctps1, Mthfd2, Chpf, Ehhadh
2	Fatty acid metabolism	4.00	Elovl6, Acaca, LOC102549542, Ehhadh
3	Biosynthesis of antibiotics	4.00	Tat, Pycr1, Psat1, Ehhadh
4	Biosynthesis of amino acids	3.00	Tat, Pycr1, Psat1
5	Carbon metabolism	3.00	Psat1, Me1, Ehhadh
6	PI3K-Akt signaling pathway	4.00	Fgf21, Myc, Vwf, Col6a2
7	Propanoate metabolism	2.00	Acaca, Ehhadh
8	Fatty acid elongation	2.00	Elovl6, LOC102549542
9	Biosynthesis of unsaturated fatty acids	2.00	Elovl6, LOC102549542
10	Pyruvate metabolism	2.00	Me1, Acaca

**Note:** rank by enrichment score of *p-value*.

As shown in Table 2, the top 10 pathways (rank by enrichment *p-value*) were obtained, which might be the important regulatory pathways to explain the anti-hepatic steatosis mechanisms of BAL. It was found that metabolic pathways, fatty acid metabolism, fatty acid elongation and biosynthesis of unsaturated fatty acids were closely related to hepatic steatosis and all of which were regulated by BAL. Importantly, among these hepatic fatty acids metabolic pathways, it was found that fructose-induction significantly increased ELOVL6 expression (almost 20 fold change, vs. CTR group), whereas BAL treatment markedly reduced its expression in rat liver (nearly 5-fold change vs. Fructose group). Similar conclusions were obtained in the RT-qPCR and Western-blot analyses (Figures 3C–E).

### BAL Changes the Content of Medium-And Long-Chain Fatty Acids in Rat Liver

**FIGURE 4:**
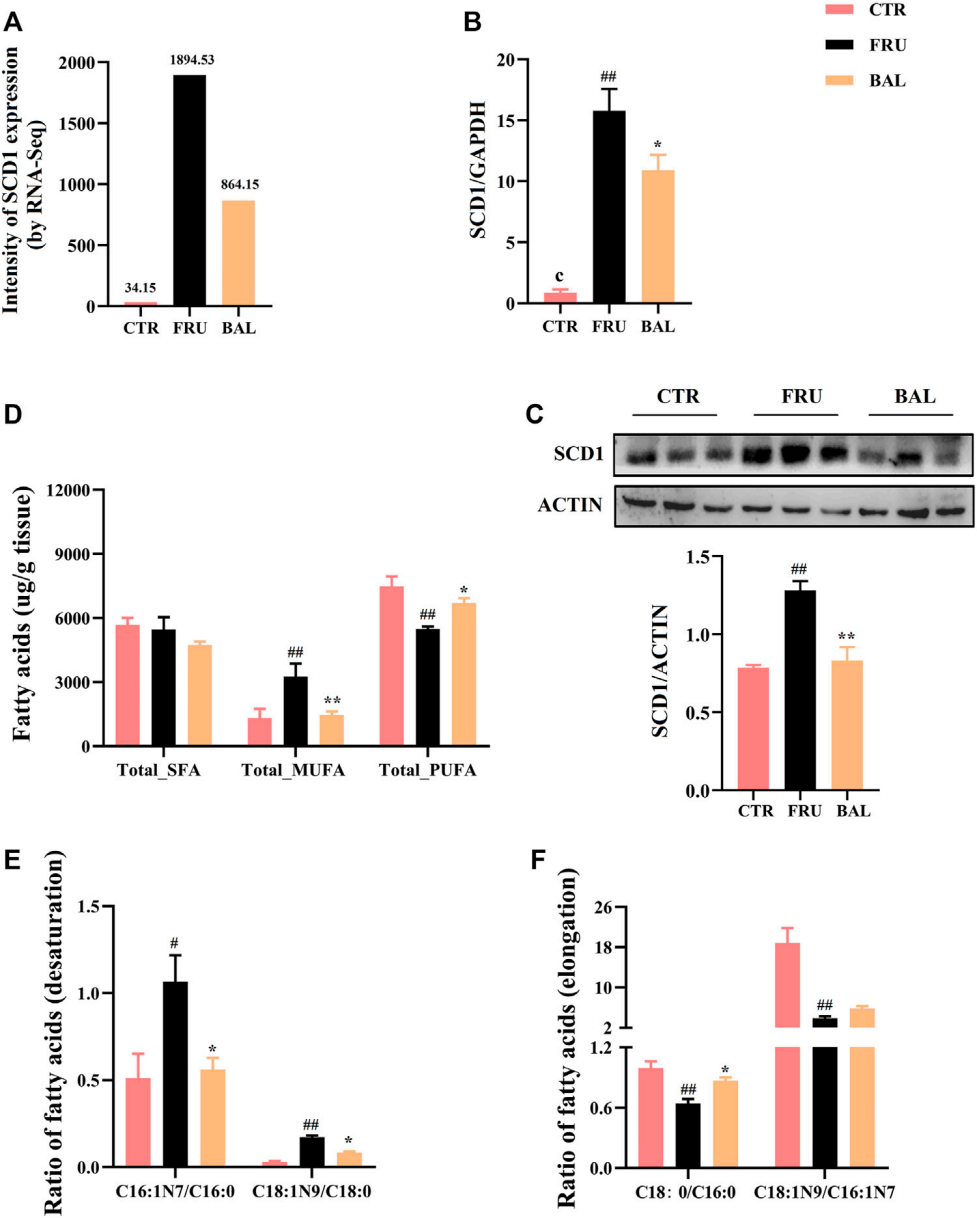
Effects on hepatic fatty acid composition and SCD1 expression caused by BAL treatment in fructose-fed rats. **(A)** SCD1 expression by RNA-sequencing analysis. **(B)** SCD1 mRNA level. **(C)** SCD1 expression in protein level. **(D)** Total of SFA, MUFA and PUFA in rat liver. **(E)** SCD1 activity. **(F)** ELOVL6 activity. Data were represented as Mean ± SEM (*n* = 6/group), ^*##*^ *p < 0.01*, ^*#*^ *p < 0.05*, compared with CTR; **p < 0.01, *p < 0.05, compared to FRU.

The metabolism of medium- and long-chain fatty acids influences fatty acid synthesis process. For instance, elongation and desaturation of long-chain FAs are crucial steps in DNL. Hence, we also investigated SCD1 (also known as SCD in rat) expression by BAL treatment. Results showed that BAL treatment significantly reversed the increase in SCD1 expression caused by fructose both at mRNA and protein level (Figures 4A–C).

Given that elongation and desaturation of long-chain fatty acids are critical steps in hepatic DNL, and play an important role in the development of hepatic steatosis. Meanwhile, having demonstrated the inhibition effect of BAL on ELOVL6 and SCD1 expression, hence we were more concentrated on the changes of fatty acid composition with BAL treatment in rat liver. Therefore, hepatic targeted metabolomics analysis was conducted to detect the medium- and long-chain fatty acids in rat liver. The contents of FAs were shown in Table 3. Notably, fructose significantly increased the content of C16:0 (methyl palmitate), C16:1N7 (methyl palmitoleate), and C18:1N9 (methyl oleate), decreased the content of C18:0 (methyl stearate), C18:2N6 (methyl linoleate), and C20:4N6 (methyl arachidonate). However, BAL treatment reduced the contents of C16:0, C16:1N7 and C18:1N9, but increased the content of C20:4N6 in rat liver. Of all categories of FAs, the total saturated fatty acids (SFA) were not changed in the three groups (Figure 4D). Moreover, fructose increased the level of total monounsaturated fatty acids (MUFA) and decreased the content of total polyunsaturated fatty acids (PUFA). In contrast, BAL treatment decreased the levels of MUFA (Figure 4D). Further analysis revealed that fructose decreased the ratio of C18:0/C16:0 instead of increasing it as expected, which was reversed by BAL treatment. In addition, the increase of C16:1N7/C16:0 and C18:1N9/C18:0 ratios induced by fructose was abolished by BAL treatment, indicating that BAL decreased the desaturation activity of long-chain FAs such as C16:0 and C18:0 (Figures 4E,F).

**TABLE 3:** The content of FFAs in rat liver (mean ± sem, unit:μg/g tissue).

FFAs	CTR	FRU	BAL
C6:0	0.0238 ± 0.0023	0.0133 ± 0.0006	0.0137 ± 0.0020
C8:0	0.3731 ± 0.1823	0.1711 ± 0.0292	0.0698 ± 0.0115
C10:0	0.1370 ± 0.0175	0.3766 ± 0.0615	0.0368 ± 0.0116
C12:0	0.8425 ± 0.1864	2.7780 ± 0.6246	0.5333 ± 0.1021
C14:0	35.1954 ± 7.8683	108.0984 ± 16.2117	34.2952 ± 4.5530
C14:1N5	1.4973 ± 0.5920	8.5985 ± 2.8922	1.3809 ± 0.2114
C15:0	21.2824 ± 3.3441	27.7182 ± 4.9968	22.1581 ± 1.4381
C15:1N5	1.4569 ± 0.1253	3.2773 ± 0.6461	2.0436 ± 0.2747
**C16:0**	**2,789.2323 ± 95.7927**	**3,353.4312 ± 322.5141** ^**##**^	**2,478.4479 ± 120.0530** ^**^
**C16:1N7**	**82.0571 ± 23.0386**	**572.7815 ± 64.5532** ^**##**^	**206.8358 ± 20.6805** ^*^
C17:0	60.4128 ± 7.9081	43.0747 ± 6.1495	47.5305 ± 2.3036
C17:1N7	9.2513 ± 3.4910	25.2344 ± 5.3490	13.4863 ± 2.0289
**C18:0**	**2,749.9536 ± 141.9080**	**2085.5766 ± 81.0418** ^**##**^	**2,135.5337 ± 66.6622**
**C18:1N9**	**1,385.8858 ± 365.2102**	**2,256.1982 ± 379.8247** ^**##**^	**1,197.7949 ± 144.8305** ^**^
C18:1TN9	6.0330 ± 0.9812	5.5797 ± 0.5003	5.8391 ± 0.3230
**C18:2N6**	**3,099.4381 ± 643.6702**	**2,277.0893 ± 497.7303** ^**##**^	**1969.9773 ± 109.1275**
C18:2TTN6	125.5125 ± 19.8160	176.5474 ± 28.3267	160.4374 ± 14.3505
C18:3N3	69.5923 ± 22.8171	54.8562 ± 25.0860	24.5476 ± 4.2529
C18:3N6	48.9816 ± 18.0330	30.3419 ± 10.5761	14.2424 ± 0.8417
C20:0	9.2276 ± 1.2421	6.2865 ± 0.4552	5.8662 ± 0.2218
C20:2N6	77.9030 ± 8.6679	64.0637 ± 13.7177	67.1992 ± 2.1656
C20:3N6	123.3998 ± 29.0607	128.1710 ± 14.7050	120.0093 ± 8.6532
**C20:4N6**	**3,494.6614 ± 175.7534**	**2,508.3840 ± 100.7882** ^**##**^	**2,951.4288 ± 86.5039** ^*^
C20:5N3	32.2346 ± 9.4160	16.7495 ± 3.5611	11.2942 ± 0.9658
C21:0	0.2835 ± 0.0403	0.1884 ± 0.0077	0.1833 ± 0.0203
C22:0	2.6981 ± 0.2829	1.9068 ± 0.0612	1.7246 ± 0.1039
C22:1N9	1.4688 ± 0.1394	1.7873 ± 0.2066	1.3007 ± 0.0534
C22:2N6	1.5374 ± 0.7306	0.7179 ± 0.1873	0.5172 ± 0.0519
C22:4N6	105.8847 ± 17.1469	107.0775 ± 25.1376	76.4959 ± 4.5467
C22:5N3	156.6995 ± 9.3627	127.3216 ± 25.5878	117.1898 ± 13.5488
C22:5N6	100.4668 ± 31.1574	89.8382 ± 11.2632	104.4745 ± 12.4036
C22:6N3	966.8221 ± 99.2398	695.7326 ± 39.8893	892.0808 ± 35.6812
C23:0	1.4308 ± 0.1752	1.0329 ± 0.0670	0.9796 ± 0.0616
C24:0	6.2681 ± 0.2504	5.1953 ± 0.1192	4.6291 ± 0.2379

**Note:** ^##^p < 0.01, ^#^p < 0.05, compared with CTR, group; ^**^p < 0.01, ^*^p < 0.05, compared with FRU, group. FFAs with significant differences among these three groups have been marked in bold.

**FIGURE 5:**
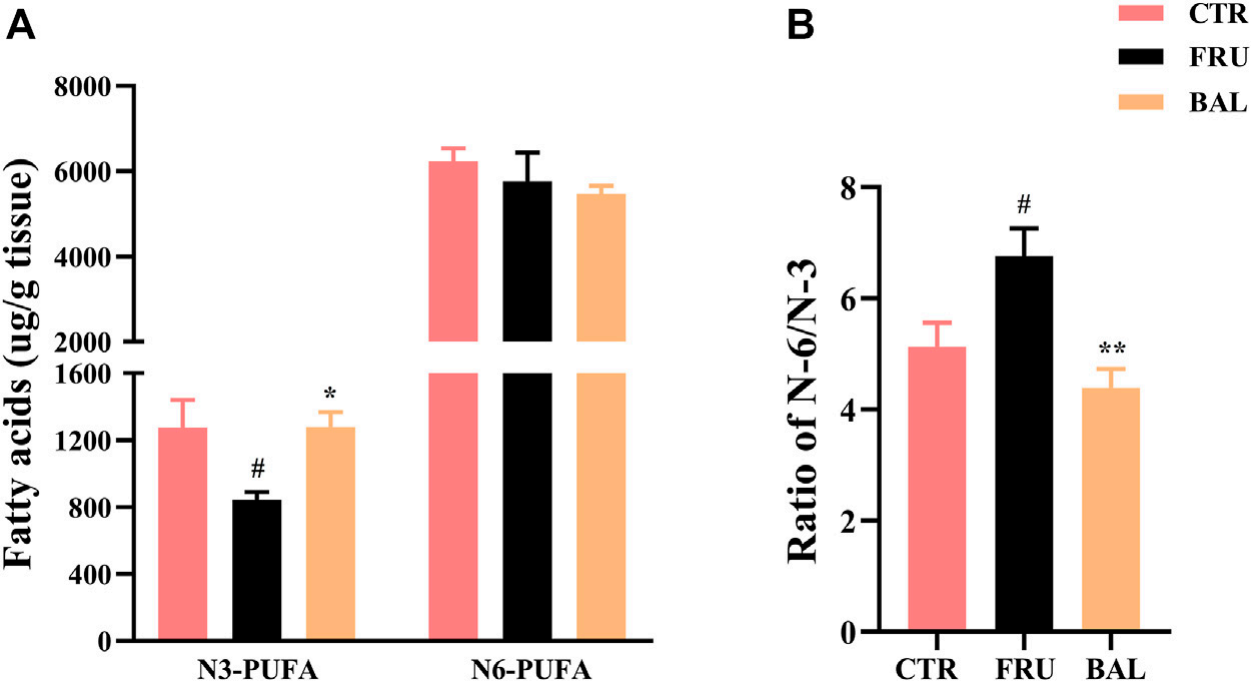
N-3 and N-6 PUFA in liver of fructose-fed rats. **(A)** Contents of the hepatic N-3 and N-6 PUFA. **(B)** Ratio of N-6 and N-3 PUFA. Data were represented as Mean ± SEM (*n* = 6/group), ^*##*^ *p < 0.01*, ^*#*^ *p < 0.05*, compared with CTR; **p < 0.01, *p < 0.05, compared to FRU.

As shown in Figure 5, prolonged administration of fructose significantly decreased the content of omage-3 (N-3) PUFA and increased the ratio of omage-6 (N-6)/N-3 PUFA in rat liver (“N-3” or “N-6” is depending on the positioning of the first double bond of nitrogen atom). Interestingly, we found that BAL treatment increased N-3 PUFA (Figure 5A), and decreased the ratio of N-6/N-3 PUFA (Figure 5B) in liver of fructose-fed rats. Meanwhile, it was observed that there were no evident differences in the levels of N-6 PUFA among these three groups (including CTR, Fructose-fed and BAL treatment groups).

#### BAL Suppresses Hepatic Lipid Accumulation via Inhibiting ChREBP1/SREBP1c-Mediated DNL

Carbohydrate response element-binding protein (ChREBP) and sterol regulatory element-binding protein 1c (SREBP1c) are the key transcription factors of genes involved in hepatic DNL, which are closely implicated in the development of fructose-induced hepatic steatosis (Ter Horst and Serlie, 2017). Excessive fructose consumption increases acetyl-CoA carboxylase (ACC), fatty acid synthase (FASN), SCD1 and ELOVL6 expression by activating the transcription of ChREBP and SREBP1c, and that alters FAs composition in liver (Bae et al., 2016; Hibi et al., 2021). Given that BAL significantly decreased SCD1 and ELOVL6 expression, and affected the composition of FAs (reduced C16:0 and C18:1, C18:1/C18:0, and C16:1/C16:0, ratios), we next explored the effects of BAL treatment on the pathways of SREBP1 and ChREBP1-mediated hepatic DNL in fructose-fed rats.

**FIGURE 6:**
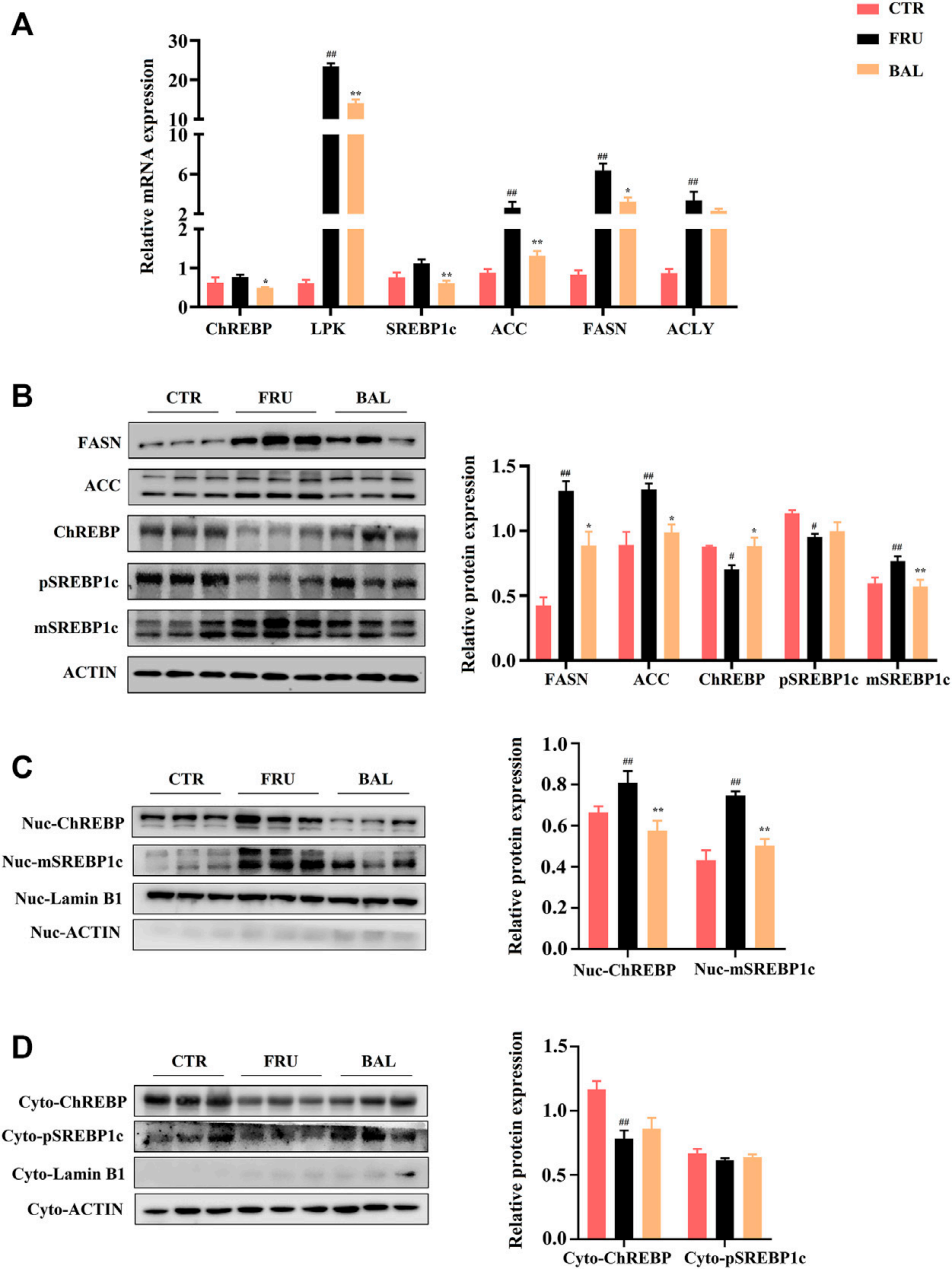
Effects of key molecules on ChREBP1/SREBP1c-mediated DNL pathway by BAL treatment. **(A)** The related genes expressions by RT-qPCR analysis. **(B)** Expressions of proteins in liver tissue. **(C)** The nuclear proteins expressions in liver. **(D)** Proteins expressions in cytoplasm of rat liver. Data were represented as Mean ± SEM (*n* = 6/group), ^*##*^ *p < 0.01*, ^*#*^ *p < 0.05*, compared with CTR; **p < 0.01, *p < 0.05, compared to FRU.

As shown in Figure 6A, it was observed that fructose significantly increased the mRNA levels of FAs synthetic genes, including those of liver pyruvate kinase (LPK), ACC, FASN, and ATP citrate lyase (ACLY). Although the expressions of ChREBP and SREBP1c were not significantly different between fructose-fed group and CTR group, we found that BAL treatment obviously decreased the mRNA levels of ChREBP, SREBP1c, ACC, FASN and ACLY compared with fructose-fed group. At protein level, excessive fructose consumption increased the expressions of FASN and ACC, which were significantly inhibited by BAL. Importantly, it was found that fructose significantly decreased the total expressions of precursor SREBP1c (pSREBP1c) and ChREBP, increased mature SREBP1c (mSREBP1c) expression in rat liver, and these effects were reversed by BAL [which also significantly reduced mSREBP1c expression, furthermore, BAL treatment strengthened ChREBP level and did not dramatically affect pSREBP1c expression (*p = 0.76*) (Figure 6B)]. Next, we investigated the expression of SREBP1c and ChREBP in cytoplasm and nuclear. As shown in Figure 6C, BAL treatment abolished the increased in expression levels of mSREBP1c and ChREBP in the nuclear caused by fructose. In the cytoplasm, BAL treatment caused no obvious changes in pSREBP1c and ChREBP expression (Figure 6D). Those evidence suggested that BAL could suppress fatty acid DNL by inhibiting the nuclear translocation activity of SREBP1c and ChREBP.

### BAL Preserves the Balance of Fatty Acid Composition by Activating AMPK Signal Pathway

**FIGURE 7:**
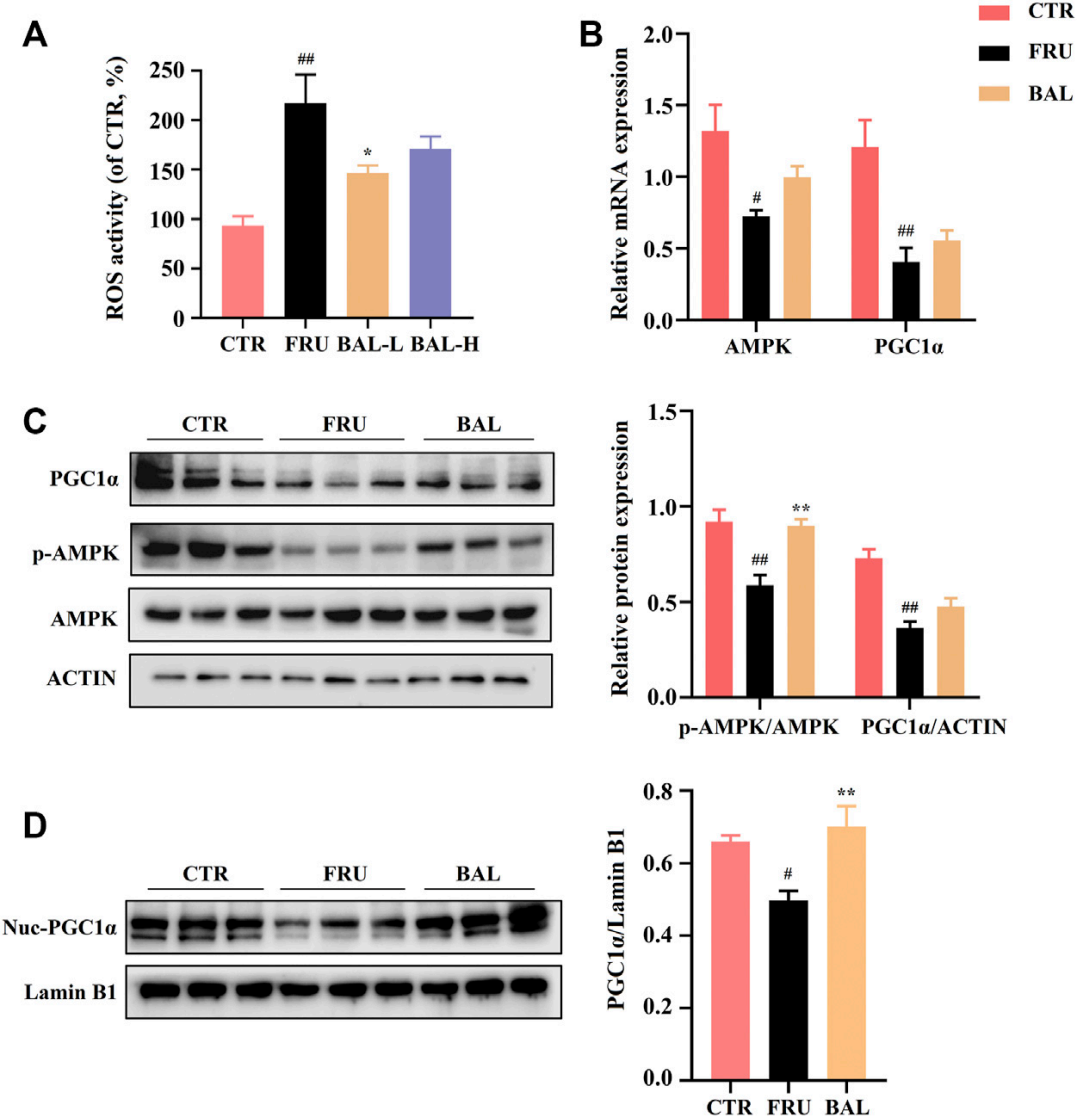
Effects of hepatic ROS and AMPK-PGC1α pathway by BAL treatment in fructose-fed rat liver. **(A)** ROS activity in rat liver. **(B)** mRNA level of AMPK and PGC1α. **(C)** PGC1α expression and AMPK phosphorylation level (Thr 172). **(D)** The nuclear expression of PGC1α. Data were represented as Mean ± SEM (*n* = 6/group), ^*##*^ *p < 0.01*, ^*#*^ *p < 0.05*, compared with CTR; **p < 0.01, *p < 0.05, compared to FRU.

Hepatic oxidative stress influences the development of hepatic steatosis. Excessive reactive oxygen species (ROS) production impairs FAs metabolism by disrupting mitochondrial homeostasis, and this may contribute to the occurrence of fructose-induced NAFLD (Jegatheesan and De Bandt, 2017; Ter Horst and Serlie, 2017; Chen et al., 2020). A previous study demonstrated that *elovl6* ^*−/−*^ knockout in mice resulted in elevation of 5′-AMP-activated protein kinase (AMPK) activity by modifying FAs composition elicited extensive ROS production (Sunaga et al., 2016). The AMPK regulates bioenergy metabolism, under physiological state, ROS activates AMPK, which in turn triggers the peroxisome proliferator-activated receptor-γ co-activator-1α (PGC1α)-dependent antioxidant response leading to a reduction in mitochondrial ROS production (Rabinovitch et al., 2017). A recent report showed that excessive intake of energy substances caused excessive ROS production, which impairs AMPK signaling by inducing the dissociation of AMPKα from liver kinase B1 (LKB1) (Jiang et al., 2021). As expected, fructose significantly aggravated ROS production in rat liver, which was reversed by a low dose of BAL (Figure 7A). At the molecular level, although BAL treatment increases mRNA level of AMPK, fructose significantly decreased the phosphorylation of AMPK (Thr 172), and this effect was reversed by BAL treatment (Figure 7B,C). Furthermore, although it was observed that BAL could not affect the reduction of PGC1α expression both in mRNA and protein levels, BAL increased the nuclear expression of PGC1α in rat liver (Figures 7B–D).

**FIGURE 8:**
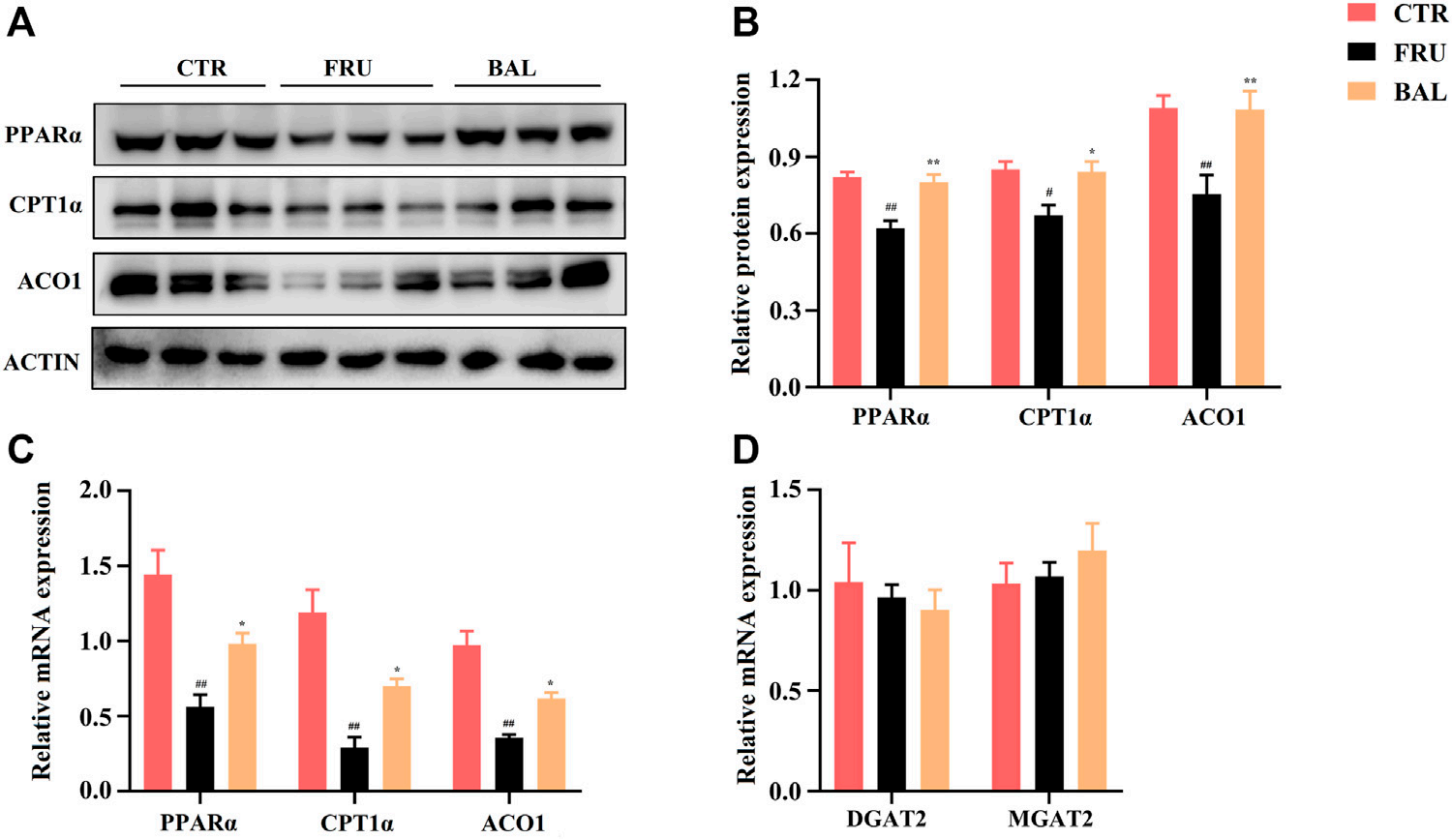
Effects of BAL treatment on PPARα elicited fatty acids oxidation pathway and the key enzymes’ (DGAT2 and MGAT2) levels in triglyceride synthesis of fructose-fed rat liver. **(A,B)** Protein expressions of ACO1, CPT1α and PPARα by western-blot analysis. **(C)** FAO associated genes expressions of PPARα, CPT1α and ACO1. **(D)** mRNA levels of DGAT2 and MGAT2. Data were represented as Mean ± SEM (*n* = 6/group), ^*##*^ *p < 0.01*, ^*#*^ *p < 0.05*, compared with CTR; **p < 0.01, *p < 0.05, compared to FRU.

ACC and SREBP1c are the important downstream regulatory factors of AMPK. The phosphorylation of AMPK can enhance ACC activity and suppress SREBP-1c cleavage. In this way, AMPK decreases SREBP1c-mediated DNL and enhances carnitine palmitoyl transferase 1α (CPT1α) activity of promoting FAs oxidation (FAO) in the liver (Steinberg et al., 2006; Li et al., 2011). As shown in Figures 6A,B, BAL treatment counteracted the increase the levels of SREBP1c (mRNA) and mSREBP1c (protein) induced by fructose. Meanwhile, fructose increased ACC expression and decreased CPT1α expression both in mRNA and protein levels, and these changes were reversed by BAL treatment (Figures 6A,B and Figures 8A,B). Together, these results show that BAL decreases ACC and SREBP1c activity, thereby reducing DNL in liver, and enhancing FAO by increasing CPT1α expression via AMPK signaling.

### BAL Ameliorates Hepatic Lipid Accumulation Through Promoting PPARα Elicited Fatty Acids Oxidation

The peroxisome proliferator-activated receptor *α* (PPARα) is an important protein that regulates liver energy balance by influencing progresses of hepatic fatty acid oxidation, DNL, gluconeogenesis and glycolysis. It can be activated by the long-chain fatty acids, especially PUFA (arachidonic acid, ALA, EPA, and DHA, the ligands of PPARα for activation of FAO in liver (Nakamura et al., 2014; Korbecki et al., 2019). As shown in Figure 4, treatment with BAL significantly increased hepatic PUFA content. Specifically, it increased arachidonic acid and DHA levels (Table 3). Based on these evidence we asked whether BAL treatment resulted in activation of PPAR signaling pathway? To test this hypothesis, we examined the effects by BAL treatment on PPARα pathway in rat liver. As expected, BAL treatment increased the expression of PPARα both at mRNA and protein level, and it also significantly enhanced the expression of CPT1α and ACO1 (Figures 8A–C), the two downstream regulators of PPARα, which are responsible for mitochondrial and peroxisomal FAO. These evidence demonstrated that BAL might accelerate FAO by activation of PPARα in rat liver.

In addition, we measured the levels of Diacylglycerol Acyltransferase 2 (DGAT2) and Monoacylglycerol Acyltransferase 2 (MGAT2), two critical enzymes involved in TG synthesis. Results showed that BAL treatment did not alter the mRNA levels of DGAT2 and MGAT2 in the liver of fructose-fed rats (Figure 8D).

## Discussion

In this study, we aimed to explore the protective effect of BAL on fructose-fed rats and its potential mechanisms. To observe the effects of BAL on rats effectively and conveniently, we chose the low dose (25 mg/kg) and high dose (100 mg/kg) of BAL for treatment based on the basal dose of BAL in rats referenced by previous studies (Ahad et al., 2014; El-Bassossy et al., 2014; Zhang J. et al., 2017). Surprisingly and interestingly, BAL treatment at low-dose (25 mg/kg) showed a better lowing-hepatic steatosis effect. Actually, some natural products (or Traditional Chinese Medicine) did not represent the better dosage-dependent effects owing to their drug selectivity, and it may be related to their pharmacokinetic parameters with oral absorption (as the nonlinear pharmacokinetics) (Ludden, 1991). Such as supplementation with green tea extract produced a dose-independent beneficial and parallel effect on the lipid profile and insulin resistance in NaCl-induced hypertensive rats (Stepien et al., 2018). Large Yellow Tea ameliorated glucose intolerance and insulin resistance in a dose-independent manner (Xu et al., 2018), and capsaicin evoked giant migrating contractions in a dose-independent manner at 5 and 10 mg dose in dogs (Hayashi et al., 2010). Importantly, the multi-peaks of the plasma concentration-time curves were observed and the non-linear pharmacokinetics for BAL and its metabolite baicalin were found after orally administrating different doses of BAL in monkeys (Tian et al., 2012), which may cause the result that low dose of BAL showed a stronger protective effect on hepatic steatosis in fructose-induced rats. Moreover, it was observed that BAL treatment did not alter the body weight and the ratio of liver/body, which indicated that BAL alleviated hepatic steatosis might be irrelevant with losing weight and alleviating liver weight. The present study was consistent with our previous study and the other similar researches that fructose-fed (4–6 weeks) could not significantly alter the body weight and serum TC level, as well as no obvious changes in HDL, TC and ALT levels caused by fructose-consumption in rats (Abd et al., 2016; Bai et al., 2020; Kim and Min, 2020; Yuan et al., 2020). ALT and AST are the indexes for evaluating liver injure, although it was found that fructose-fed could not change ALT level, fructose-fed seemed to increase AST level compared with CTR group rats (*p = 0.12*), importantly, we observed that BAL treatment significantly decreased serum AST, TG and LDL-C levels in rats, indicating BAL could relieve the metabolic disorders and mild liver injure caused by excessive fructose consumption in rats.

ELOVL6 is a fatty acyl elongase involved in the fatty acid synthesis, which progressively performs the initial and rate-limiting condensing reaction required for microsomal elongation of long-chain fatty acids, primarily elongating 12-, 14-, or 16-carbon chain saturated or monounsaturated fatty acids to 18-carbon chain fatty acids (Matsuzaka et al., 2002; Moon et al., 2014). Evidence suggests that hepatic ELOVL6 deficiency alters the fatty acid composition and decreases the long-chain fatty acids, which contributes to the decline of the ratio of the fatty acid (including C18:0/C16:0, C18:1/C16:1), and ultimately, it inhibits fatty acid synthesis via an effect on SREBP1c-medicated lipogenic pathway (Matsuzaka et al., 2007; Matsuzaka and Shimano, 2009). Additionally, SCD is a member of the fatty acid desaturase family that is elevated by dietary carbohydrates and catalyzes the conversion of SFA to MUFA. It catalyzes the conversion of long-chain FAs from C16:0 and C18:0 to C16:1 and C18:1 in the DNL progression, which is highly expressed in NAFLD (Flowers and Ntambi, 2009). In mice lacking SCD, fatty acid synthesis is decreased and fatty acid oxidation is increased, which protects against hepatic steatosis or insulin resistance (Flowers and Ntambi, 2009). In this study, it was discovered that BAL significantly decreased ELOVL6 and SCD1 expression that had been up-regulated by fructose, as confirmed by western-blot and RT-qPCR analyses. Unexpectedly, fructose-fed mice were unable to enhance the ratios of C18:0/C16:0 and C18:1/C16:1 (ELOLV6 activity), which may be due to the effects of fructose-induction on the strong activity of SCD1, which results in more C18:0 being converted to C18:1 via desaturation reaction. However, whether elovl6 and SCD1 compete for FAs metabolism or other metabolic pathways in rats with excessive fructose intake remains unknown and requires further investigation. Nonetheless, fructose-fed mice had significantly increased C16:1/C16:0 and C18:1/C18:0 ratios, indicating that fructose-fed mice could significantly increase SCD1 activity, whereas BAL treatment decreased SCD1 activity. These findings suggested that BAL may affect the FAs’ composition through regulating their elongation and desaturation by suppressing ELOVL6 and SCD1 expression or activity.

SREBP1 and ChREBP play an important role in hepatic fatty acid DNL, which promotes the expression of lipogenesis-related genes, including ACC, FASN, ELOVL6, and SCD1. SREBP1c is the main transcription factor that mediates the activation of lipogenesis. It is activated by SREBP cleavage activating protein (SCAP) for splicing from the inactive pSREBP1c in the endoplasmic reticulum (ER) to the active mSREBP1c in the nucleus, and the latter activates transcription of lipogenic genes (Moon, 2017). On the other hand, ChREBP is also a critical transcription factor involved in hepatic DNL, its trans-activity is regulated by a dephosphorylation mechanism, and dephosphorylated ChREBP forms a heterodimer with max like protein X (MLX) in the nucleus to exert transcriptional regulatory effects (Iizuka and Horikawa, 2008). In this study, BAL treatment significantly decreased the mRNA levels of ChREBP and SREBP1c. More importantly, we found that BAL significantly decreased the expression of the active forms of these two transcription factors, mSREBP1c and ChREBP, in the nuclear compartment, while increasing the expression of pSREBP1c and ChREBP in the cytoplasm of the fructose-fed rat liver. These findings showed that BAL may suppress hepatic DNL through regulating the activities of SREBP1c and ChREBP, which are the important regulatory mechanisms of BAL for relieving fructose-induced hepatic steatosis.

PUFA is gaining popularity as a result of its health benefits, which include immune-regulation, lipid oxidation, and antioxidant activity (Di Nunzio et al., 2016; Hu et al., 2018). Linoleic acid (C18: 2N6) and *α*-linolenic acid (C18:3N3) are essential FAs that can not be synthesized by humans and must therefore be obtained exclusively through diet. They are then endogenously metabolized to long-chain unsaturated N-6 and N-3 products by the desaturase and elongase enzymes, respectively (Koletzko et al., 2011). A high N-6/N-3 ratio may result in ER stress (an important pathogenic factor in NAFLD), which has been observed in NAFLD patients (Parker et al., 2012; Okada et al., 2018). N-3 PUFA are key regulators of hepatic gene transcription involved in lipogenesis and oxidation. N-3 PUFA may inhibit DNL by reducing the amount of mature SREBP-1 available in the nucleus, which may be attributed to N-3 PUFA-induced reduction in the effective half-life of SREBP-1 mRNA (Yahagi et al., 1999; Xu et al., 2001; Yoshikawa et al., 2002). Additionally, investigations have demonstrated that N-3 PUFA are potent PPARα activators, capable of upregulating several genes involved in fatty acid oxidation, including CYP4A1, ACO and CPT1 (El-Badry et al., 2007; Masterton et al., 2010). These findings suggested that regulating N-3 PUFA may be a promising therapy for NALFD. Furthermore, we discovered that BAL treatment significantly increased N-3 PUFA and decreased the ratio of N-6/N-3. As previously described, N-6 or N-3 PUFA cannot be synthesized in mammals due to a lack of metabolic enzymes (D12, which catalyzes the conversion of MUFA to PUFA, as well as the conversion of C18:1n-9 to C18:2n-6 (Spector, 1999; Rohrbach, 2009). Interestingly, we discovered that BAL treatment increased the C18:2n-6 content of rat liver, the main substrate for PUFA synthesis, which may be related to BAL’s ability to regulate intestinal absorption in fructose-fed rats. Previous studies have shown that high fructose intake can readily result in intestinal-barrier deterioration and endotoxaemia, thereby affecting the intestinal absorption function of nutrients (Zhang DM. et al., 2017; Todoric et al., 2020). Meanwhile, evidence suggested that BAL could improve the intestinal structure to re-balance the gut microbial composition in response to radiation-induced injuries (Wang et al., 2020), and it was found that BAL modulated the gut microbiota composition and improved gut barrier function in streptozotocin and high-fat-diet-induced diabetic rats (Zhang et al., 2018). Therefore, we hypothesized that BAL might regulate the gut microbiota to improve PUFA absorption (particularly C18:2n-6 in diets), thereby increasing N-3 PUFA or decreasing the N-6/N-3 PUFA ratio, which may be a novel mechanism for BAL in the treatment of NAFLD but requires further confirmation and study.

AMPK is a critical regulator of energy metabolism, and excessive energy substance intake results in excess production of ROS, which inhibits AMPK signaling by inducing the dissociation of AMPKα and LKB1 (Jiang et al., 2021). There is evidence that fructose promotes hepatic DNL, inhibits mitochondrial beta-oxidation of long-chain fatty acids, triglyceride formation, and steatosis followed by activation of protein fructosylation and the formation of ROS in the liver (Rohrbach, 2009). As expected, we found that fructose-fed significantly increased palmitic acid (C16:0) and oleic acid (C18:1N9) levels in the rat liver, the two primary raw materials for triglyceride synthesis that were decreased by BAL treatment. Furthermore, fructose-fed rats generated excess ROS, which reduced AMPK activity in the liver. This inhibition was reversed by BAL treatment, demonstrating that BAL treatment might activate the AMPK signaling pathway by inhibiting ROS production caused by excessive fructose intake in rats. Additionally, a previous study suggested that unsaturated fatty acids increase ROS generation by partially inhibiting electron transport and altering membrane fluidity in the forward electron transport mode of the liver, whereas FAs decrease ROS production through their uncoupling action in reverse electron transport conditions (Schonfeld and Wojtczak, 2007). Although there are still disputes regarding the production of ROS and fatty acids, which may be related to the inconsistency of experimental results *in vivo or in vitro*, FAs certainly play an important role in a variety of mitochondrial processes, including mitochondrial calcium homeostasis, gene expression, respiratory function, ROS production, and mitochondrial apoptosis (Rohrbach, 2009; Vergani, 2019). Therefore, we hypothesized that BAL decreased ROS production was related to its effects on altering FAs components, such as decreasing MUFA and increasing PUFA, particularly N3-PUFA in the liver, due to its beneficial effects on modulating lipogenesis, ER stress, and oxidative stress via AMPK activation (Okada et al., 2018; Scorletti and Byrne, 2018; Yang et al., 2019). However, potential mechanisms need to be further identified in future research.

It is evidence that indicated that BAL inhibits hepatic *de novo* lipogenesis via suppressing SREBP1c and ChREBP activities, thereby reversing the high expressions of FASN, ACC and SCD expression in oleic acid-induced hepatocytes and high-fat-diet (HFD) induced mice (Xing et al., 2021), we got the similar results in fructose-induced rats. These evidences suggested that excessive HFD or fructose consumption both enhance the activities of key regulators in hepatic DNL, SREBP1c and ChREBP in liver, and which were suppressed by BAL treatment. In regulation of hepatic fatty acid oxidation, a previous study indicated BAL may increase fatty acid oxidation by strengthening CPT1α and PPARα expression in HFD-induced mice (Pu et al., 2012). While in the present study, it was also found that BAL could enhance fatty acid oxidation in fructose-induced rats, which might be attributed to the high expressions of CPT1α, PPARα and ACO1. Moreover, BAL also activate AMPK by increasing its phosphorylation level. Furthermore, it was observed that BAL significantly increase hepatic polyunsaturated fatty acid, the ligands for the activation PPARα in hepatic fatty acid oxidation. Different from the HFD-induced hepatic steatosis, long-term fructose-intake mainly causes the excessive production of lipogenesis, hence hepatic *de novo* lipogenesis plays a significant role in this development (Jegatheesan and De Bandt, 2017). Our study focused on the main pathological process caused by excessive fructose-fed in rats, and systematically explained the regulatory mechanism of BAL in the relationship with DNL and hepatic fatty acid composition. On the other hand, differing from HFD-induced hepatic steatosis (Sun et al., 2020; Xing et al., 2021), fructose-fed did not cause the obvious changes in TC of rats, which was consistent by our previous studies (Bai et al., 2020; Yuan et al., 2020), implying BAL may not affect the synthetic and metabolic progress of TC in fructose-induced hepatic steatosis rat model.

## Conclusion

This study demonstrates that BAL ameliorates hepatic steatosis in rats fed by fructose over an extended period. Furthermore, BAL not only alters fatty acid composition by suppressing SREBP1c/ChREBP mediated fatty acid DNL and elongation, but also promotes fatty acids oxidation by activating the AMPK and PPARα signal pathways. Furthermore, our study enriches the pharmacological activity of BAL on various hepatic steatosis models, and provides a systematic molecular mechanism of BAL for the treatment of fructose-induced hepatic steatosis. In conclusion, BAL is a promising natural product for the treatment of hepatic steatosis in a “muti-targets, muti-regulation pathways” manner.

## Data Availability

The datasets presented in this study can be found in online repositories. The names of the repository/repositories and accession number(s) can be found below: https://www.ncbi.nlm.nih.gov/bioproject/PRJNA831629.
