# Supplementary material for: Baicalein Prevents Fructose-Induced Hepatic Steatosis in Rats: In the Regulation of Fatty Acid De Novo Synthesis, Fatty Acid Elongation and Fatty Acid Oxidation
Source: Front Pharmacol. 2022 Jun 30;13:917329. doi: 10.3389/fphar.2022.917329 (PMC9280198; doi:10.3389/fphar.2022.917329)
Supplement: Supplementary file 1 [file DataSheet1.DOCX]

**Supplemental_Material**

**Includes Table S1, Table S2, Table S3, Figure S1 and Figure S2**

**Table. S1|** Differently expressed genes (FRU vs CTR). FC: fold change; FRU: fructose-induced group rats; CTR; control group rats.

| **Gene ID** | **Gene name** | **FC(FRU/CTR)** | **Log_2_FC(FRU/CTR)** | **p-value** | **p-adjust** | **Significant** | **Regulate** |
| --- | --- | --- | --- | --- | --- | --- | --- |
| ENSRNOG00000019328 | Phgdh | 108.709 | 6.764321843 | 6.39593E-57 | 8.53537E-53 | yes | up |
| ENSRNOG00000020704 | Tkfc | 14.995 | 3.90643958 | 4.35602E-45 | 2.90656E-41 | yes | up |
| ENSRNOG00000020420 | Pklr | 25.069 | 4.64785891 | 5.20545E-42 | 2.31556E-38 | yes | up |
| ENSRNOG00000013971 | Psat1 | 45.917 | 5.520945617 | 3.12969E-41 | 1.04414E-37 | yes | up |
| ENSRNOG00000007546 | Asns | 46.315 | 5.533400646 | 5.23625E-40 | 1.39755E-36 | yes | up |
| ENSRNOG00000023150 | Gpi | 6.496 | 2.699585957 | 1.89828E-35 | 4.22209E-32 | yes | up |
| ENSRNOG00000047023 | Aldh1l1 | 3.059 | 1.613176894 | 1.29227E-29 | 2.46363E-26 | yes | up |
| ENSRNOG00000010887 | RGD1309534 | 5.061 | 2.339283382 | 4.92319E-29 | 8.2125E-26 | yes | up |
| ENSRNOG00000051232 | Gbe1 | 4.693 | 2.230599948 | 1.56547E-25 | 2.32125E-22 | yes | up |
| ENSRNOG00000017895 | Eno1 | 3.347 | 1.743014306 | 2.0398E-24 | 2.72212E-21 | yes | up |
| ENSRNOG00000009715 | Me1 | 10.324 | 3.367883264 | 9.52395E-24 | 1.15543E-20 | yes | up |
| ENSRNOG00000012404 | Thrsp | 9.361 | 3.226591305 | 9.16519E-21 | 1.01925E-17 | yes | up |
| ENSRNOG00000018964 | Gss | 5.362 | 2.422745166 | 1.39359E-20 | 1.43057E-17 | yes | up |
| ENSRNOG00000016064 | Tkt | 5.106 | 2.352060989 | 5.96214E-20 | 5.6832E-17 | yes | up |
| ENSRNOG00000048949 | LOC102549542 | 31.614 | 4.982504535 | 5.6639E-19 | 5.03898E-16 | yes | up |
| ENSRNOG00000007895 | Pdhb | 3.458 | 1.789745414 | 9.58318E-19 | 7.99297E-16 | yes | up |
| ENSRNOG00000017899 | Akr7a3 | 8.173 | 3.030840786 | 1.3418E-18 | 1.05331E-15 | yes | up |
| ENSRNOG00000021046 | Sult2b1 | 10.746 | 3.425666248 | 6.92589E-18 | 5.13478E-15 | yes | up |
| ENSRNOG00000015498 | Il17rb | 10.242 | 3.35648094 | 1.1915E-17 | 8.36873E-15 | yes | up |
| ENSRNOG00000008103 | Mdh1 | 2.627 | 1.393168698 | 1.46962E-17 | 9.80605E-15 | yes | up |
| ENSRNOG00000049075 | Fabp5 | 9.741 | 3.284103469 | 5.31253E-17 | 3.37599E-14 | yes | up |
| ENSRNOG00000006807 | Aldob | 2.547 | 1.349075313 | 1.99994E-16 | 1.21314E-13 | yes | up |
| ENSRNOG00000021245 | RGD1311739 | 7.736 | 2.951671913 | 8.15868E-16 | 4.73381E-13 | yes | up |
| ENSRNOG00000036833 | Zfp385a | 3.966 | 1.987794067 | 1.18915E-15 | 6.61217E-13 | yes | up |
| ENSRNOG00000006893 | Ppm1k | 0.251 | -1.996882552 | 1.49613E-15 | 7.98633E-13 | yes | down |
| ENSRNOG00000008989 | Cryl1 | 6.005 | 2.586244295 | 2.59207E-15 | 1.33043E-12 | yes | up |
| ENSRNOG00000058726 | AABR07054837.1 | 0.08 | -3.649829842 | 4.29002E-15 | 2.12038E-12 | yes | down |
| ENSRNOG00000045636 | Fasn | 29.233 | 4.869528485 | 4.47127E-15 | 2.13104E-12 | yes | up |
| ENSRNOG00000011052 | Gars | 3.155 | 1.657741147 | 1.01983E-14 | 4.69296E-12 | yes | up |
| ENSRNOG00000042237 | Tm6sf2 | 4.223 | 2.078146563 | 4.93354E-14 | 2.1946E-11 | yes | up |
| ENSRNOG00000012681 | Lgals9 | 3.052 | 1.609979748 | 5.78708E-14 | 2.49125E-11 | yes | up |
| ENSRNOG00000019982 | Ethe1 | 4.007 | 2.002421659 | 1.10642E-13 | 4.6141E-11 | yes | up |
| ENSRNOG00000029830 | Adm2 | 464.683 | 8.860103064 | 2.4431E-13 | 9.87976E-11 | yes | up |
| ENSRNOG00000006388 | Pygl | 4.054 | 2.019199198 | 5.10478E-13 | 2.00363E-10 | yes | up |
| ENSRNOG00000003442 | Adora1 | 7.792 | 2.961947585 | 8.07437E-13 | 3.07864E-10 | yes | up |
| ENSRNOG00000018487 | Slc3a2 | 4.586 | 2.197245005 | 9.54218E-13 | 3.53723E-10 | yes | up |
| ENSRNOG00000017693 | Slc2a5 | 18.489 | 4.208566137 | 1.53297E-12 | 5.52906E-10 | yes | up |
| ENSRNOG00000010833 | Mthfd2 | 13.467 | 3.751406682 | 2.13318E-12 | 7.4914E-10 | yes | up |
| ENSRNOG00000018755 | Acss2 | 5.28 | 2.40041118 | 3.14482E-12 | 1.07609E-09 | yes | up |
| ENSRNOG00000034013 | Acaca | 11.83 | 3.564320937 | 6.21431E-12 | 2.07325E-09 | yes | up |
| ENSRNOG00000025459 | Mars1 | 2.297 | 1.199978885 | 6.51728E-12 | 2.12129E-09 | yes | up |
| ENSRNOG00000061379 | C7 | 0.245 | -2.027131795 | 1.08574E-11 | 3.44982E-09 | yes | down |
| ENSRNOG00000019484 | Slc6a9 | 4.339 | 2.117404382 | 2.19243E-11 | 6.80418E-09 | yes | up |
| ENSRNOG00000008907 | Fggy | 2.193 | 1.132867483 | 2.36803E-11 | 7.18214E-09 | yes | up |
| ENSRNOG00000002643 | Ugdh | 3.021 | 1.595261836 | 2.70307E-11 | 8.01609E-09 | yes | up |
| ENSRNOG00000008047 | Khk | 3.214 | 1.684234634 | 3.13617E-11 | 9.09829E-09 | yes | up |
| ENSRNOG00000053769 | Soat2 | 3.28 | 1.713655697 | 3.76247E-11 | 1.0683E-08 | yes | up |
| ENSRNOG00000036682 | Pycr1 | 11.25 | 3.491868088 | 4.09705E-11 | 1.13906E-08 | yes | up |
| ENSRNOG00000016807 | Oat | 0.18 | -2.470121708 | 5.33488E-11 | 1.45294E-08 | yes | down |
| ENSRNOG00000028812 | Trim80 | 19.784 | 4.3062333 | 8.20087E-11 | 2.18881E-08 | yes | up |
| ENSRNOG00000020651 | Cars | 3.384 | 1.75870266 | 9.75254E-11 | 2.55191E-08 | yes | up |
| ENSRNOG00000008106 | Shmt2 | 3.068 | 1.617330445 | 1.11755E-10 | 2.86801E-08 | yes | up |
| ENSRNOG00000017473 | Ttc25 | 10.419 | 3.381181516 | 1.27464E-10 | 3.20944E-08 | yes | up |
| ENSRNOG00000001656 | Kcnj15 | 25.531 | 4.674164666 | 1.79562E-10 | 4.4375E-08 | yes | up |
| ENSRNOG00000003068 | Mrnip | 3.958 | 1.984655025 | 2.19431E-10 | 5.3242E-08 | yes | up |
| ENSRNOG00000018397 | Dnph1 | 4.525 | 2.177948638 | 2.33842E-10 | 5.57253E-08 | yes | up |
| ENSRNOG00000011561 | Nln | 2.795 | 1.482819386 | 2.56658E-10 | 6.00896E-08 | yes | up |
| ENSRNOG00000009708 | Tas1r1 | 14.877 | 3.895052535 | 2.9202E-10 | 6.71897E-08 | yes | up |
| ENSRNOG00000019260 | Ceacam20 | 6.1 | 2.608878369 | 3.12235E-10 | 7.06232E-08 | yes | up |
| ENSRNOG00000029223 | Prss32 | 20.995 | 4.391990283 | 4.38064E-10 | 9.74327E-08 | yes | up |
| ENSRNOG00000001517 | Pdk1 | 2.698 | 1.431893171 | 4.83082E-10 | 1.05684E-07 | yes | up |
| ENSRNOG00000010753 | Aig1 | 0.149 | -2.741954623 | 5.37586E-10 | 1.15711E-07 | yes | down |
| ENSRNOG00000009117 | Otub2 | 5.97 | 2.57778207 | 5.96726E-10 | 1.26402E-07 | yes | up |
| ENSRNOG00000030719 | Csmd1 | 8.85 | 3.14564445 | 7.22937E-10 | 1.48979E-07 | yes | up |
| ENSRNOG00000017914 | Cavin3 | 5.124 | 2.357350372 | 7.25636E-10 | 1.48979E-07 | yes | up |
| ENSRNOG00000007213 | Yars1 | 3.097 | 1.63081265 | 7.49916E-10 | 1.51631E-07 | yes | up |
| ENSRNOG00000002616 | Slc5a10 | 188.347 | 7.557247014 | 8.34359E-10 | 1.66187E-07 | yes | up |
| ENSRNOG00000033262 | Reep6 | 2.955 | 1.56310981 | 1.27239E-09 | 2.49707E-07 | yes | up |
| ENSRNOG00000000925 | Psph | 2.823 | 1.497471066 | 2.595E-09 | 5.01887E-07 | yes | up |
| ENSRNOG00000020083 | Scly | 2.866 | 1.519238042 | 3.85367E-09 | 7.34675E-07 | yes | up |
| ENSRNOG00000000903 | Asl | 0.358 | -1.480495159 | 3.94179E-09 | 7.40889E-07 | yes | down |
| ENSRNOG00000012582 | Eif4ebp1 | 2.823 | 1.497196927 | 4.53702E-09 | 8.40925E-07 | yes | up |
| ENSRNOG00000012929 | Wsb1 | 0.148 | -2.753303721 | 6.33296E-09 | 1.15772E-06 | yes | down |
| ENSRNOG00000003515 | Ephx1 | 2.511 | 1.328371663 | 1.40018E-08 | 2.52505E-06 | yes | up |
| ENSRNOG00000016924 | Acly | 11.348 | 3.504319216 | 1.7457E-08 | 3.10619E-06 | yes | up |
| ENSRNOG00000021130 | Abcc8 | 5.025 | 2.329098781 | 1.95308E-08 | 3.42945E-06 | yes | up |
| ENSRNOG00000018630 | LOC108351137 | 2.366 | 1.242322678 | 2.0214E-08 | 3.50333E-06 | yes | up |
| ENSRNOG00000021808 | Tecr | 1.809 | 0.855379049 | 2.10852E-08 | 3.57135E-06 | yes | up |
| ENSRNOG00000010519 | Cyp39a1 | 1.885 | 0.914744114 | 2.11418E-08 | 3.57135E-06 | yes | up |
| ENSRNOG00000009994 | Dlat | 3.253 | 1.701698196 | 2.25485E-08 | 3.76137E-06 | yes | up |
| ENSRNOG00000032560 | Cyp3a23/3a1 | 0.308 | -1.700675469 | 2.90363E-08 | 4.78382E-06 | yes | down |
| ENSRNOG00000014314 | Slc39a4 | 2.296 | 1.199127158 | 4.87387E-08 | 7.93193E-06 | yes | up |
| ENSRNOG00000001113 | Mmd2 | 0.009 | -6.858810093 | 5.23548E-08 | 8.41777E-06 | yes | down |
| ENSRNOG00000046053 | Nudt10 | 0.195 | -2.356072201 | 5.54861E-08 | 8.81503E-06 | yes | down |
| ENSRNOG00000016222 | Ppp1r3g | 0.042 | -4.563075718 | 6.04179E-08 | 9.44615E-06 | yes | down |
| ENSRNOG00000020995 | Fut1 | 28.592 | 4.837546477 | 6.08744E-08 | 9.44615E-06 | yes | up |
| ENSRNOG00000002753 | Adam11 | 0.151 | -2.728207025 | 6.3196E-08 | 9.69368E-06 | yes | down |
| ENSRNOG00000005420 | Abcg8 | 0.051 | -4.305161537 | 7.32121E-08 | 1.11025E-05 | yes | down |
| ENSRNOG00000020255 | Sars1 | 2.451 | 1.29314061 | 8.61622E-08 | 1.29195E-05 | yes | up |
| ENSRNOG00000010275 | Slc17a9 | 2.423 | 1.27692954 | 1.02481E-07 | 1.51957E-05 | yes | up |
| ENSRNOG00000018553 | Pitpnm1 | 0.455 | -1.135446948 | 1.26069E-07 | 1.84878E-05 | yes | down |
| ENSRNOG00000020525 | Col5a3 | 22.067 | 4.463830674 | 1.37704E-07 | 1.99745E-05 | yes | up |
| ENSRNOG00000061527 | Gck | 8.355 | 3.062597796 | 1.57009E-07 | 2.25299E-05 | yes | up |
| ENSRNOG00000013552 | Scd | 35.204 | 5.137674907 | 1.69363E-07 | 2.39664E-05 | yes | up |
| ENSRNOG00000008245 | AABR07054614.1 | 6.541 | 2.709543024 | 1.70611E-07 | 2.39664E-05 | yes | up |
| ENSRNOG00000045949 | Faah | 1.984 | 0.988748845 | 1.74544E-07 | 2.42635E-05 | yes | up |
| ENSRNOG00000016753 | Slc14a1 | 26.539 | 4.730053363 | 1.88226E-07 | 2.58956E-05 | yes | up |
| ENSRNOG00000061821 | AC109891.1 | 1.976 | 0.982846205 | 1.97849E-07 | 2.69417E-05 | yes | up |
| ENSRNOG00000005602 | Mthfd1 | 1.96 | 0.971052889 | 2.01421E-07 | 2.71511E-05 | yes | up |
| ENSRNOG00000047247 | Ptprs | 2.939 | 1.55536445 | 2.11831E-07 | 2.82689E-05 | yes | up |
| ENSRNOG00000050360 | Urad | 2.441 | 1.287221503 | 2.30017E-07 | 3.03919E-05 | yes | up |
| ENSRNOG00000020035 | Cyp17a1 | 0.147 | -2.761734742 | 2.53275E-07 | 3.31369E-05 | yes | down |
| ENSRNOG00000017852 | Nars1 | 1.905 | 0.929933367 | 2.77096E-07 | 3.59014E-05 | yes | up |
| ENSRNOG00000001792 | Slc12a8 | 40.07 | 5.324457322 | 3.12328E-07 | 4.00771E-05 | yes | up |
| ENSRNOG00000000867 | Vars1 | 1.947 | 0.961555393 | 3.27461E-07 | 4.16188E-05 | yes | up |
| ENSRNOG00000028348 | Lhx8 | 5.171 | 2.370338676 | 3.3368E-07 | 4.20091E-05 | yes | up |
| ENSRNOG00000002615 | Pmm2 | 1.818 | 0.862156882 | 4.02078E-07 | 5.01471E-05 | yes | up |
| ENSRNOG00000031263 | Haao | 1.723 | 0.784651015 | 4.27958E-07 | 5.28805E-05 | yes | up |
| ENSRNOG00000020369 | Igf2 | 0.11 | -3.178910511 | 4.35808E-07 | 5.33565E-05 | yes | down |
| ENSRNOG00000013925 | Nox4 | 0.35 | -1.515207745 | 4.7692E-07 | 5.78591E-05 | yes | down |
| ENSRNOG00000056847 | Gsta3 | 7.777 | 2.959258314 | 4.869E-07 | 5.85377E-05 | yes | up |
| ENSRNOG00000022268 | Pnpla3 | 60.013 | 5.907212784 | 5.00853E-07 | 5.96775E-05 | yes | up |
| ENSRNOG00000007410 | Dab1 | 0.176 | -2.50476046 | 5.57782E-07 | 6.58627E-05 | yes | down |
| ENSRNOG00000021174 | Macrod1 | 1.879 | 0.909875278 | 5.62634E-07 | 6.58627E-05 | yes | up |
| ENSRNOG00000043193 | Smim1 | 2.621 | 1.389989281 | 5.68316E-07 | 6.59144E-05 | yes | up |
| ENSRNOG00000020698 | Rnd2 | 0.363 | -1.463336778 | 5.72954E-07 | 6.59144E-05 | yes | down |
| ENSRNOG00000008079 | Ugp2 | 2.299 | 1.200880332 | 6.32154E-07 | 7.21034E-05 | yes | up |
| ENSRNOG00000033209 | Bmp8b | 75.739 | 6.242955384 | 6.71334E-07 | 7.5294E-05 | yes | up |
| ENSRNOG00000018304 | Lars1 | 2.581 | 1.367687295 | 6.71412E-07 | 7.5294E-05 | yes | up |
| ENSRNOG00000046643 | Cyp3a9 | 0.212 | -2.24054522 | 6.96604E-07 | 7.74682E-05 | yes | down |
| ENSRNOG00000025476 | Tmem252 | 3.798 | 1.925129975 | 7.16516E-07 | 7.90241E-05 | yes | up |
| ENSRNOG00000005177 | Tp53i3 | 2.246 | 1.167668467 | 7.54745E-07 | 8.25579E-05 | yes | up |
| ENSRNOG00000020990 | Fgf21 | 8.338 | 3.059700908 | 7.96112E-07 | 8.63749E-05 | yes | up |
| ENSRNOG00000000811 | Pkib | 0.191 | -2.391167883 | 8.58622E-07 | 9.24057E-05 | yes | down |
| ENSRNOG00000018778 | Cadm1 | 2.027 | 1.019076718 | 8.94035E-07 | 9.54472E-05 | yes | up |
| ENSRNOG00000003666 | Jchain | 0.323 | -1.628573277 | 9.04813E-07 | 9.58312E-05 | yes | down |
| ENSRNOG00000020554 | Slc50a1 | 2.603 | 1.380178097 | 1.07471E-06 | 0.000112929 | yes | up |
| ENSRNOG00000009592 | Cyb5r3 | 1.631 | 0.706133361 | 1.29856E-06 | 0.000135385 | yes | up |
| ENSRNOG00000032895 | Cyp4f4 | 0.533 | -0.907870355 | 1.33951E-06 | 0.000138572 | yes | down |
| ENSRNOG00000017766 | Ca12 | 34.153 | 5.093921424 | 1.63747E-06 | 0.000168037 | yes | up |
| ENSRNOG00000014616 | Iars1 | 3.156 | 1.658149426 | 1.64952E-06 | 0.000168037 | yes | up |
| ENSRNOG00000003330 | Acsf2 | 3.435 | 1.780358314 | 1.72352E-06 | 0.000174245 | yes | up |
| ENSRNOG00000008031 | Cacna2d4 | 4.135 | 2.04779445 | 1.77298E-06 | 0.000177898 | yes | up |
| ENSRNOG00000010941 | Tifa | 0.308 | -1.697054937 | 1.82805E-06 | 0.000182055 | yes | down |
| ENSRNOG00000010210 | Slc7a11 | 28.483 | 4.832015673 | 1.85159E-06 | 0.000183033 | yes | up |
| ENSRNOG00000016957 | Igfbp2 | 0.134 | -2.895618001 | 2.22098E-06 | 0.000217934 | yes | down |
| ENSRNOG00000031004 | Cyp2j4 | 0.379 | -1.400496153 | 2.4205E-06 | 0.000235778 | yes | down |
| ENSRNOG00000059447 | AC109901.2 | 0.257 | -1.960031463 | 2.48268E-06 | 0.000240083 | yes | down |
| ENSRNOG00000003123 | Rpa1 | 2.096 | 1.067816446 | 2.50606E-06 | 0.000240599 | yes | up |
| ENSRNOG00000004637 | Fbxo7 | 2.264 | 1.17892771 | 2.57944E-06 | 0.000245876 | yes | up |
| ENSRNOG00000015591 | Cndp2 | 1.726 | 0.787696508 | 2.62037E-06 | 0.000248006 | yes | up |
| ENSRNOG00000016322 | Camk2n1 | 0.266 | -1.908829952 | 2.74017E-06 | 0.000257022 | yes | down |
| ENSRNOG00000057092 | Slfn4 | 0.297 | -1.749042939 | 2.75415E-06 | 0.000257022 | yes | down |
| ENSRNOG00000015642 | Pabpc4 | 1.681 | 0.749569871 | 2.79791E-06 | 0.000259292 | yes | up |
| ENSRNOG00000053384 | Bmp7 | 2.541 | 1.34550885 | 2.90042E-06 | 0.000266939 | yes | up |
| ENSRNOG00000011203 | Farp1 | 1.843 | 0.881788171 | 2.99906E-06 | 0.000273252 | yes | up |
| ENSRNOG00000026866 | Syn3 | 8.507 | 3.088679045 | 3.00996E-06 | 0.000273252 | yes | up |
| ENSRNOG00000018044 | Phyh | 0.336 | -1.574848876 | 3.04769E-06 | 0.000274807 | yes | down |
| ENSRNOG00000057101 | AABR07050652.1 | 14.253 | 3.833202337 | 3.16388E-06 | 0.000283369 | yes | up |
| ENSRNOG00000001797 | Umps | 2.291 | 1.19590573 | 3.19481E-06 | 0.000284232 | yes | up |
| ENSRNOG00000016648 | Gulo | 1.635 | 0.709620829 | 3.98593E-06 | 0.000352267 | yes | up |
| ENSRNOG00000033697 | Casp4 | 2.856 | 1.513893135 | 4.20197E-06 | 0.000368916 | yes | up |
| ENSRNOG00000002930 | Ppl | 0.297 | -1.749753136 | 4.40202E-06 | 0.000383954 | yes | down |
| ENSRNOG00000016967 | Hfe | 1.793 | 0.842001657 | 4.8222E-06 | 0.000417872 | yes | up |
| ENSRNOG00000015880 | Dpep1 | 0.378 | -1.404374096 | 5.75757E-06 | 0.000495709 | yes | down |
| ENSRNOG00000057620 | Slc6a8 | 0.402 | -1.314222899 | 6.44827E-06 | 0.000551616 | yes | down |
| ENSRNOG00000011541 | Cygb | 0.427 | -1.229157107 | 6.71126E-06 | 0.000570457 | yes | down |
| ENSRNOG00000005250 | Abcg5 | 0.101 | -3.312662159 | 7.21472E-06 | 0.00060937 | yes | down |
| ENSRNOG00000036942 | Tnfrsf9 | 6.078 | 2.60358101 | 7.31891E-06 | 0.000614282 | yes | up |
| ENSRNOG00000009889 | Pgm1 | 1.593 | 0.671463247 | 7.73386E-06 | 0.000640723 | yes | up |
| ENSRNOG00000015149 | Nrg4 | 0.122 | -3.02925856 | 7.77721E-06 | 0.000640723 | yes | down |
| ENSRNOG00000023520 | Cs | 1.655 | 0.726847245 | 7.83874E-06 | 0.000640723 | yes | up |
| ENSRNOG00000000900 | Tpst1 | 0.518 | -0.948174132 | 7.85846E-06 | 0.000640723 | yes | down |
| ENSRNOG00000059586 | AABR07015080.2 | 0.451 | -1.150267282 | 7.874E-06 | 0.000640723 | yes | down |
| ENSRNOG00000049829 | AABR07060872.1 | 0.323 | -1.62842829 | 8.40705E-06 | 0.000679952 | yes | down |
| ENSRNOG00000034190 | Ighm | 0.297 | -1.749740066 | 9.18152E-06 | 0.000738117 | yes | down |
| ENSRNOG00000014050 | Ptges2 | 1.784 | 0.835415047 | 9.67181E-06 | 0.000772876 | yes | up |
| ENSRNOG00000004554 | Dcn | 0.422 | -1.243129626 | 1.12189E-05 | 0.000891171 | yes | down |
| ENSRNOG00000020942 | Plekha4 | 2.923 | 1.547685846 | 1.13664E-05 | 0.000897542 | yes | up |
| ENSRNOG00000012136 | Mrps30 | 1.828 | 0.8706359 | 1.16595E-05 | 0.00091527 | yes | up |
| ENSRNOG00000004177 | Myo1a | 187.484 | 7.550622892 | 1.19967E-05 | 0.000936236 | yes | up |
| ENSRNOG00000043416 | Bcl3 | 0.38 | -1.397235828 | 1.20786E-05 | 0.000937142 | yes | down |
| ENSRNOG00000059834 | Cib2 | 9.65 | 3.270522807 | 1.24318E-05 | 0.000958975 | yes | up |
| ENSRNOG00000007650 | Cd63 | 0.291 | -1.781495116 | 1.25386E-05 | 0.000959479 | yes | down |
| ENSRNOG00000000041 | Slc26a1 | 2.176 | 1.121974851 | 1.26362E-05 | 0.000959479 | yes | up |
| ENSRNOG00000021513 | Rtn4rl2 | 2.195 | 1.134349448 | 1.26541E-05 | 0.000959479 | yes | up |
| ENSRNOG00000053891 | AABR07018078.1 | 1.968 | 0.976579044 | 1.30282E-05 | 0.000977 | yes | up |
| ENSRNOG00000016448 | Eef2k | 0.554 | -0.853296069 | 1.30315E-05 | 0.000977 | yes | down |
| ENSRNOG00000003228 | Mid1ip1 | 3.38 | 1.756883117 | 1.32681E-05 | 0.000989175 | yes | up |
| ENSRNOG00000012181 | Lpl | 0.374 | -1.419658645 | 1.47311E-05 | 0.001092151 | yes | down |
| ENSRNOG00000004303 | Timp3 | 0.51 | -0.970446873 | 1.48316E-05 | 0.001093526 | yes | down |
| ENSRNOG00000060052 | Tdrd15 | 0.36 | -1.474210118 | 1.54174E-05 | 0.001130465 | yes | down |
| ENSRNOG00000036604 | Ifit2 | 2.275 | 1.185746082 | 1.64879E-05 | 0.001202353 | yes | up |
| ENSRNOG00000019924 | Thop1 | 1.705 | 0.769520294 | 1.72335E-05 | 0.001245152 | yes | up |
| ENSRNOG00000015807 | Pyroxd2 | 1.86 | 0.895455338 | 1.72614E-05 | 0.001245152 | yes | up |
| ENSRNOG00000003508 | Ergic1 | 2.769 | 1.469210276 | 1.79349E-05 | 0.00128678 | yes | up |
| ENSRNOG00000026344 | Togaram2 | 0.055 | -4.17527059 | 1.80349E-05 | 0.001287034 | yes | down |
| ENSRNOG00000000924 | Slc7a1 | 10.338 | 3.369908396 | 1.91427E-05 | 0.001358825 | yes | up |
| ENSRNOG00000007682 | Gria3 | 0.118 | -3.079045329 | 2.14479E-05 | 0.001514407 | yes | down |
| ENSRNOG00000016573 | Dgat2 | 1.595 | 0.673292473 | 2.22954E-05 | 0.001563004 | yes | up |
| ENSRNOG00000019206 | Nupr1 | 3.042 | 1.605106181 | 2.23705E-05 | 0.001563004 | yes | up |
| ENSRNOG00000027888 | Cmss1 | 2.609 | 1.383684331 | 2.26321E-05 | 0.00157305 | yes | up |
| ENSRNOG00000056445 | AABR07004530.1 | 0.349 | -1.520550588 | 2.40428E-05 | 0.00166244 | yes | down |
| ENSRNOG00000014119 | Farsb | 1.883 | 0.912969392 | 2.43721E-05 | 0.001676522 | yes | up |
| ENSRNOG00000006949 | C1qbp | 2.158 | 1.109810181 | 2.50237E-05 | 0.001712522 | yes | up |
| ENSRNOG00000007218 | Leap2 | 3.273 | 1.710399629 | 2.55439E-05 | 0.001739201 | yes | up |
| ENSRNOG00000011361 | Slc37a4 | 1.95 | 0.963419398 | 2.58552E-05 | 0.001751462 | yes | up |
| ENSRNOG00000032690 | Ube2e2 | 1.859 | 0.894370785 | 2.67624E-05 | 0.00180376 | yes | up |
| ENSRNOG00000010331 | Ctsb | 1.965 | 0.974678596 | 2.73772E-05 | 0.001826742 | yes | up |
| ENSRNOG00000036959 | RGD1561551 | 44.249 | 5.46758098 | 2.78935E-05 | 0.001851934 | yes | up |
| ENSRNOG00000007060 | Plin2 | 2.283 | 1.190855464 | 2.81215E-05 | 0.001857829 | yes | up |
| ENSRNOG00000046763 | Adssl1 | 2.099 | 1.069610314 | 2.83837E-05 | 0.001865914 | yes | up |
| ENSRNOG00000062027 | AABR07035343.1 | 0.389 | -1.363398775 | 2.88035E-05 | 0.001884232 | yes | down |
| ENSRNOG00000018453 | Nop2 | 1.98 | 0.985418456 | 3.02853E-05 | 0.001967456 | yes | up |
| ENSRNOG00000045999 | Tnfaip8l1 | 1.553 | 0.634830552 | 3.03706E-05 | 0.001967456 | yes | up |
| ENSRNOG00000058780 | Igfbp1 | 0.229 | -2.125054884 | 3.0638E-05 | 0.001975192 | yes | down |
| ENSRNOG00000049299 | AABR07051532.2 | 0.18 | -2.470108417 | 3.11031E-05 | 0.001995533 | yes | down |
| ENSRNOG00000047745 | AABR07035348.1 | 0.362 | -1.46636341 | 3.22551E-05 | 0.00205954 | yes | down |
| ENSRNOG00000011647 | S100a6 | 0.239 | -2.066529098 | 3.27157E-05 | 0.002070602 | yes | down |
| ENSRNOG00000038001 | Slc25a1 | 2.154 | 1.107204009 | 3.27386E-05 | 0.002070602 | yes | up |
| ENSRNOG00000022189 | Htatip2 | 2.198 | 1.136089876 | 3.40908E-05 | 0.002145949 | yes | up |
| ENSRNOG00000009434 | RGD1310507 | 1.701 | 0.766723852 | 3.4642E-05 | 0.002170411 | yes | up |
| ENSRNOG00000032745 | Slc17a3 | 2.856 | 1.513779553 | 3.51386E-05 | 0.002191237 | yes | up |
| ENSRNOG00000005515 | Rhbdl3 | 0.39 | -1.35954959 | 3.55008E-05 | 0.002203528 | yes | down |
| ENSRNOG00000048874 | Gckr | 2.152 | 1.105347248 | 3.80701E-05 | 0.002352063 | yes | up |
| ENSRNOG00000020060 | Atf5 | 2.05 | 1.035866005 | 4.01449E-05 | 0.002468817 | yes | up |
| ENSRNOG00000002393 | Eprs | 2.056 | 1.039817931 | 4.10013E-05 | 0.002509917 | yes | up |
| ENSRNOG00000012457 | Cyc1 | 1.635 | 0.709079417 | 4.36652E-05 | 0.002660785 | yes | up |
| ENSRNOG00000047895 | Mettl1 | 2.578 | 1.366027157 | 4.39698E-05 | 0.002667169 | yes | up |
| ENSRNOG00000001388 | Sds | 0.028 | -5.149093417 | 4.60114E-05 | 0.00277838 | yes | down |
| ENSRNOG00000016348 | Tat | 0.307 | -1.702897619 | 4.65858E-05 | 0.002800393 | yes | down |
| ENSRNOG00000057713 | Cav2 | 0.494 | -1.017588309 | 4.70201E-05 | 0.002813825 | yes | down |
| ENSRNOG00000016356 | Got1 | 0.362 | -1.467792235 | 4.88433E-05 | 0.002909882 | yes | down |
| ENSRNOG00000052925 | NEWGENE_621351 | 0.356 | -1.489203147 | 5.24846E-05 | 0.003099146 | yes | down |
| ENSRNOG00000011228 | Layn | 0.309 | -1.692524191 | 5.32509E-05 | 0.003130543 | yes | down |
| ENSRNOG00000002839 | Slc19a2 | 0.515 | -0.957471247 | 5.47761E-05 | 0.003185237 | yes | down |
| ENSRNOG00000002013 | Sidt1 | 0.115 | -3.11529321 | 5.48561E-05 | 0.003185237 | yes | down |
| ENSRNOG00000003244 | Ltc4s | 0.306 | -1.710199817 | 5.48973E-05 | 0.003185237 | yes | down |
| ENSRNOG00000013313 | Nceh1 | 0.394 | -1.341938713 | 5.78638E-05 | 0.003338307 | yes | down |
| ENSRNOG00000008415 | Nab2 | 0.26 | -1.944643412 | 5.80358E-05 | 0.003338307 | yes | down |
| ENSRNOG00000055466 | Derl2 | 1.777 | 0.829228818 | 6.07913E-05 | 0.003454064 | yes | up |
| ENSRNOG00000011526 | Pcsk6 | 2.309 | 1.206986114 | 6.11383E-05 | 0.003454064 | yes | up |
| ENSRNOG00000028630 | Ksr2 | 0.447 | -1.160088989 | 6.11989E-05 | 0.003454064 | yes | down |
| ENSRNOG00000020701 | Brca1 | 0.316 | -1.660389951 | 6.16065E-05 | 0.003454064 | yes | down |
| ENSRNOG00000012881 | Fgl2 | 0.585 | -0.772735471 | 6.16965E-05 | 0.003454064 | yes | down |
| ENSRNOG00000015910 | Lmntd1 | 2.732 | 1.449872011 | 6.18803E-05 | 0.003454064 | yes | up |
| ENSRNOG00000013409 | Gclm | 2.012 | 1.00828418 | 6.22065E-05 | 0.003454064 | yes | up |
| ENSRNOG00000039745 | Pm20d1 | 1.606 | 0.683655122 | 6.23776E-05 | 0.003454064 | yes | up |
| ENSRNOG00000013719 | Dph5 | 1.929 | 0.94780403 | 6.6641E-05 | 0.003674893 | yes | up |
| ENSRNOG00000056076 | LOC103694877 | 3.187 | 1.672286486 | 6.75496E-05 | 0.003694466 | yes | up |
| ENSRNOG00000006589 | Mif | 3.187 | 1.672286486 | 6.75496E-05 | 0.003694466 | yes | up |
| ENSRNOG00000019232 | RGD1311345 | 1.577 | 0.657574883 | 6.87852E-05 | 0.003746686 | yes | up |
| ENSRNOG00000057569 | Ahnak | 0.496 | -1.011758955 | 7.35447E-05 | 0.003989651 | yes | down |
| ENSRNOG00000042182 | Mroh2a | 4.489 | 2.166325479 | 7.40169E-05 | 0.00399478 | yes | up |
| ENSRNOG00000011937 | Sgtb | 3.425 | 1.77624037 | 7.44382E-05 | 0.00399478 | yes | up |
| ENSRNOG00000015160 | Gem | 0.116 | -3.101694015 | 7.45534E-05 | 0.00399478 | yes | down |
| ENSRNOG00000015581 | Dnah6 | 0.117 | -3.092288407 | 7.48366E-05 | 0.00399478 | yes | down |
| ENSRNOG00000014915 | Gsr | 1.643 | 0.716455468 | 7.63082E-05 | 0.004057102 | yes | up |
| ENSRNOG00000008676 | Emp1 | 0.424 | -1.238196821 | 7.68402E-05 | 0.004069178 | yes | down |
| ENSRNOG00000015153 | Ints3 | 1.611 | 0.687659447 | 7.71611E-05 | 0.00407002 | yes | up |
| ENSRNOG00000013585 | Nmnat3 | 2.678 | 1.421290921 | 7.94552E-05 | 0.004174526 | yes | up |
| ENSRNOG00000056476 | Slc22a13 | 36.249 | 5.179870716 | 8.14679E-05 | 0.004263488 | yes | up |
| ENSRNOG00000023269 | AABR07021759.1 | 0.128 | -2.962511699 | 8.23249E-05 | 0.004275743 | yes | down |
| ENSRNOG00000010655 | Mttp | 1.543 | 0.626127098 | 8.23429E-05 | 0.004275743 | yes | up |
| ENSRNOG00000003486 | Mnda | 0.513 | -0.962297676 | 8.56219E-05 | 0.004428774 | yes | down |
| ENSRNOG00000016413 | Pstpip1 | 0.346 | -1.530190174 | 9.09775E-05 | 0.004687625 | yes | down |
| ENSRNOG00000039906 | Abo3 | 49.563 | 5.631191894 | 9.80896E-05 | 0.005034637 | yes | up |
| ENSRNOG00000011820 | Acpp | 4.784 | 2.25827481 | 9.912E-05 | 0.005068032 | yes | up |
| ENSRNOG00000043866 | AY172581.24 | 0.196 | -2.353013244 | 0.000100321 | 0.005096857 | yes | down |
| ENSRNOG00000018367 | Taldo1 | 2.145 | 1.101213171 | 0.000100448 | 0.005096857 | yes | up |
| ENSRNOG00000059468 | AABR07034573.3 | 3.022 | 1.595724585 | 0.000101001 | 0.005105542 | yes | up |
| ENSRNOG00000005479 | Slc1a2 | 0.295 | -1.761548553 | 0.000102637 | 0.005168656 | yes | down |
| ENSRNOG00000050000 | AABR07034739.1 | 0.279 | -1.840628009 | 0.000103475 | 0.005191258 | yes | down |
| ENSRNOG00000056216 | Casp7 | 1.723 | 0.784542429 | 0.000107441 | 0.005366908 | yes | up |
| ENSRNOG00000014613 | Ddah1 | 0.459 | -1.123791304 | 0.000107781 | 0.005366908 | yes | down |
| ENSRNOG00000039079 | Ces2h | 1.659 | 0.730724579 | 0.000110436 | 0.005478682 | yes | up |
| ENSRNOG00000011952 | Samm50 | 1.649 | 0.72125084 | 0.000113667 | 0.005615407 | yes | up |
| ENSRNOG00000043098 | Mt2A | 0.219 | -2.189209803 | 0.000114033 | 0.005615407 | yes | down |
| ENSRNOG00000048233 | LOC100910668 | 2.74 | 1.454007225 | 0.00011452 | 0.005615685 | yes | up |
| ENSRNOG00000056457 | Gpd1 | 2.173 | 1.119751228 | 0.000114881 | 0.005615685 | yes | up |
| ENSRNOG00000000589 | RGD1310495 | 1.587 | 0.666121224 | 0.000116372 | 0.005667836 | yes | up |
| ENSRNOG00000054742 | AABR07066188.1 | 0.171 | -2.548744989 | 0.000118256 | 0.005738638 | yes | down |
| ENSRNOG00000012495 | Podxl | 0.507 | -0.979888035 | 0.000123797 | 0.005983265 | yes | down |
| ENSRNOG00000014806 | Pnkd | 1.779 | 0.831338018 | 0.000124194 | 0.005983265 | yes | up |
| ENSRNOG00000054325 | AABR07004397.2 | 0.192 | -2.380398377 | 0.000125308 | 0.006015211 | yes | down |
| ENSRNOG00000008332 | Smo | 0.367 | -1.447727428 | 0.000126151 | 0.006033994 | yes | down |
| ENSRNOG00000011667 | Fastk | 1.696 | 0.762385174 | 0.000132862 | 0.0063323 | yes | up |
| ENSRNOG00000012447 | Setd6 | 1.618 | 0.694472359 | 0.000136557 | 0.006485237 | yes | up |
| ENSRNOG00000020110 | B4gat1 | 0.266 | -1.909588746 | 0.000137587 | 0.006510975 | yes | down |
| ENSRNOG00000021139 | Esrra | 1.547 | 0.629279709 | 0.000138696 | 0.006540289 | yes | up |
| ENSRNOG00000057201 | AABR07035782.1 | 0.08 | -3.637060531 | 0.000140002 | 0.006578609 | yes | down |
| ENSRNOG00000059443 | LOC103690028 | 0.012 | -6.3757421 | 0.000143183 | 0.006704503 | yes | down |
| ENSRNOG00000017979 | Mrto4 | 2.034 | 1.024535237 | 0.000143827 | 0.006711083 | yes | up |
| ENSRNOG00000013061 | Tusc3 | 2.432 | 1.282274197 | 0.000144809 | 0.006733355 | yes | up |
| ENSRNOG00000011276 | Tmem254 | 1.808 | 0.854403826 | 0.000146146 | 0.006766317 | yes | up |
| ENSRNOG00000020770 | Arl4d | 4.46 | 2.157056995 | 0.000146532 | 0.006766317 | yes | up |
| ENSRNOG00000007319 | Trib3 | 3.943 | 1.979140848 | 0.000148765 | 0.00684577 | yes | up |
| ENSRNOG00000057626 | Kif1b | 2.543 | 1.346343112 | 0.000152251 | 0.006982095 | yes | up |
| ENSRNOG00000020897 | Prpf19 | 1.569 | 0.650190938 | 0.000153357 | 0.007008743 | yes | up |
| ENSRNOG00000010170 | Tubb4b | 1.93 | 0.948316552 | 0.000155414 | 0.007078512 | yes | up |
| ENSRNOG00000008639 | Pabpc1 | 1.754 | 0.810308217 | 0.000161978 | 0.007352357 | yes | up |
| ENSRNOG00000009597 | Cyp4a1 | 0.277 | -1.852756599 | 0.000164379 | 0.007436046 | yes | down |
| ENSRNOG00000012876 | Slc6a13 | 0.406 | -1.298705893 | 0.000166363 | 0.007500383 | yes | down |
| ENSRNOG00000009663 | Apex1 | 2.051 | 1.036003982 | 0.00016748 | 0.007525327 | yes | up |
| ENSRNOG00000019441 | Hsd3b2 | 0.029 | -5.104855475 | 0.000173513 | 0.007770242 | yes | down |
| ENSRNOG00000021102 | Scn1b | 0.313 | -1.677289466 | 0.000175665 | 0.007840315 | yes | down |
| ENSRNOG00000003291 | Creg1 | 2.889 | 1.530393611 | 0.000180741 | 0.008039961 | yes | up |
| ENSRNOG00000012580 | Ccdc141 | 3.741 | 1.903439458 | 0.000182641 | 0.008097507 | yes | up |
| ENSRNOG00000034089 | Ttc21a | 5.487 | 2.456000256 | 0.000184407 | 0.008148696 | yes | up |
| ENSRNOG00000025444 | Lipo1 | 0.317 | -1.655227881 | 0.000185831 | 0.008184557 | yes | down |
| ENSRNOG00000050949 | Ttc39c | 1.682 | 0.750296445 | 0.000191114 | 0.008377052 | yes | up |
| ENSRNOG00000058249 | Pgk1 | 1.683 | 0.751235278 | 0.000191458 | 0.008377052 | yes | up |
| ENSRNOG00000057740 | Ccdc148 | 0.167 | -2.584133928 | 0.000202076 | 0.008812752 | yes | down |
| ENSRNOG00000026636 | Urm1 | 1.618 | 0.694247619 | 0.000212266 | 0.009226986 | yes | up |
| ENSRNOG00000005669 | Ca8 | 0.533 | -0.907695911 | 0.000214855 | 0.009304029 | yes | down |
| ENSRNOG00000017965 | Afg3l2 | 1.584 | 0.664014309 | 0.000215432 | 0.009304029 | yes | up |
| ENSRNOG00000010549 | Tspo | 3 | 1.584783749 | 0.000223294 | 0.009581547 | yes | up |
| ENSRNOG00000010262 | Hdc | 0.208 | -2.263363858 | 0.000224672 | 0.009609788 | yes | down |
| ENSRNOG00000019891 | Sgta | 1.562 | 0.643128403 | 0.000227106 | 0.009682838 | yes | up |
| ENSRNOG00000051690 | Clec9a | 0.367 | -1.444764609 | 0.000228445 | 0.009708899 | yes | down |
| ENSRNOG00000050205 | Afmid | 1.614 | 0.69056279 | 0.000231921 | 0.009825332 | yes | up |
| ENSRNOG00000001254 | Col6a2 | 0.489 | -1.033384452 | 0.000234285 | 0.009894102 | yes | down |
| ENSRNOG00000025764 | AC128848.1 | 0.218 | -2.197162474 | 0.000236931 | 0.009956512 | yes | down |
| ENSRNOG00000003743 | Dars | 1.557 | 0.638808723 | 0.000237255 | 0.009956512 | yes | up |
| ENSRNOG00000008754 | Flvcr2 | 0.447 | -1.161391092 | 0.000239891 | 0.010035563 | yes | down |
| ENSRNOG00000007136 | Anxa7 | 1.665 | 0.735431407 | 0.000244813 | 0.010191929 | yes | up |
| ENSRNOG00000031612 | Gls2 | 0.472 | -1.0840464 | 0.000245156 | 0.010191929 | yes | down |
| ENSRNOG00000019456 | Kars | 1.541 | 0.623769344 | 0.000246362 | 0.010210237 | yes | up |
| ENSRNOG00000007235 | Atp5mc1 | 2.26 | 1.176099834 | 0.000248989 | 0.010287187 | yes | up |
| ENSRNOG00000056836 | Cav1 | 0.455 | -1.135550802 | 0.000252722 | 0.010409194 | yes | down |
| ENSRNOG00000006082 | Rmdn2 | 1.817 | 0.861616962 | 0.000255688 | 0.010498935 | yes | up |
| ENSRNOG00000022009 | Mzb1 | 0.217 | -2.201790203 | 0.000260075 | 0.010593138 | yes | down |
| ENSRNOG00000052070 | Aldh1a3 | 0.475 | -1.07397156 | 0.000260363 | 0.010593138 | yes | down |
| ENSRNOG00000030788 | Olr35 | 0.163 | -2.618080283 | 0.000262494 | 0.010616739 | yes | down |
| ENSRNOG00000038365 | Plekha2 | 0.507 | -0.980456229 | 0.000263263 | 0.010616739 | yes | down |
| ENSRNOG00000045647 | Hax1 | 1.896 | 0.923175163 | 0.00026333 | 0.010616739 | yes | up |
| ENSRNOG00000006302 | Gclc | 2.381 | 1.251513133 | 0.00027649 | 0.011113728 | yes | up |
| ENSRNOG00000050828 | Vkorc1 | 1.878 | 0.909015084 | 0.00027853 | 0.011162091 | yes | up |
| ENSRNOG00000001235 | Gna12 | 1.908 | 0.931792605 | 0.000287371 | 0.011481919 | yes | up |
| ENSRNOG00000021438 | Tuba1c | 1.816 | 0.860724451 | 0.000289801 | 0.011539733 | yes | up |
| ENSRNOG00000047493 | Slco1a4 | 0.393 | -1.346111186 | 0.000290547 | 0.011539733 | yes | down |
| ENSRNOG00000013410 | Spink1l | 0.4 | -1.321828751 | 0.00029869 | 0.011827966 | yes | down |
| ENSRNOG00000018991 | Gsn | 0.411 | -1.282176338 | 0.00030183 | 0.011916918 | yes | down |
| ENSRNOG00000012061 | Prkcb | 0.29 | -1.784668779 | 0.000305054 | 0.012008683 | yes | down |
| ENSRNOG00000000512 | Slc26a8 | 1.991 | 0.993604119 | 0.000323209 | 0.012648741 | yes | up |
| ENSRNOG00000003759 | Galc | 3.318 | 1.730306623 | 0.000326412 | 0.012693824 | yes | up |
| ENSRNOG00000014652 | Aqp8 | 1.88 | 0.910353126 | 0.000327149 | 0.012693824 | yes | up |
| ENSRNOG00000002280 | Sh3bgrl | 0.579 | -0.788424165 | 0.000327214 | 0.012693824 | yes | down |
| ENSRNOG00000007431 | Shroom1 | 1.551 | 0.633176349 | 0.00033011 | 0.01276902 | yes | up |
| ENSRNOG00000057296 | AC112001.1 | 0.192 | -2.382317551 | 0.000337692 | 0.013024584 | yes | down |
| ENSRNOG00000017447 | Eif2s2 | 1.554 | 0.636370189 | 0.000339188 | 0.013044547 | yes | up |
| ENSRNOG00000008218 | Atp6v0e2 | 2.531 | 1.339989063 | 0.000340467 | 0.013056119 | yes | up |
| ENSRNOG00000010319 | Lcp1 | 0.498 | -1.0064475 | 0.000344905 | 0.013180188 | yes | down |
| ENSRNOG00000014257 | Ces4a | 2.112 | 1.078300644 | 0.000345677 | 0.013180188 | yes | up |
| ENSRNOG00000018369 | Prx | 0.422 | -1.24319793 | 0.000353897 | 0.013455141 | yes | down |
| ENSRNOG00000009515 | Pgm3 | 0.577 | -0.793683664 | 0.000357539 | 0.013555 | yes | down |
| ENSRNOG00000025584 | Agap2 | 0.343 | -1.542185934 | 0.000369857 | 0.01398226 | yes | down |
| ENSRNOG00000006327 | Rcc2 | 1.798 | 0.846646786 | 0.000373572 | 0.014082817 | yes | up |
| ENSRNOG00000019365 | Ablim3 | 0.327 | -1.614703509 | 0.000374955 | 0.014095135 | yes | down |
| ENSRNOG00000017869 | Irf8 | 0.516 | -0.955389504 | 0.000395151 | 0.014812614 | yes | down |
| ENSRNOG00000061890 | Ust5r | 0.567 | -0.81747863 | 0.000403523 | 0.015084068 | yes | down |
| ENSRNOG00000014426 | Lox | 0.194 | -2.362940548 | 0.000407286 | 0.015182223 | yes | down |
| ENSRNOG00000003583 | Smyd2 | 1.781 | 0.832471593 | 0.000410245 | 0.015249923 | yes | up |
| ENSRNOG00000002141 | Cd200 | 0.211 | -2.244152767 | 0.000414323 | 0.015321078 | yes | down |
| ENSRNOG00000021682 | Dcakd | 1.864 | 0.898061421 | 0.000414456 | 0.015321078 | yes | up |
| ENSRNOG00000019023 | Tars1 | 1.63 | 0.704531401 | 0.000435726 | 0.016062896 | yes | up |
| ENSRNOG00000017780 | Akr7a2 | 1.711 | 0.775260646 | 0.000438163 | 0.016108208 | yes | up |
| ENSRNOG00000005569 | Phospho1 | 1.811 | 0.857123119 | 0.000440283 | 0.016141693 | yes | up |
| ENSRNOG00000015290 | Tpi1 | 1.626 | 0.700933077 | 0.00044248 | 0.016177788 | yes | up |
| ENSRNOG00000007446 | Apip | 1.863 | 0.897276803 | 0.000449827 | 0.016401491 | yes | up |
| ENSRNOG00000021663 | Vxn | 0.355 | -1.49312317 | 0.000453972 | 0.016507498 | yes | down |
| ENSRNOG00000007506 | Ndufaf4 | 1.763 | 0.817806468 | 0.000456715 | 0.016562137 | yes | up |
| ENSRNOG00000061695 | Slc23a1 | 2.01 | 1.007102501 | 0.000463069 | 0.016708051 | yes | up |
| ENSRNOG00000001415 | Ap1s1 | 1.63 | 0.704591383 | 0.000463243 | 0.016708051 | yes | up |
| ENSRNOG00000019270 | P2ry6 | 0.368 | -1.44140115 | 0.000464739 | 0.01671684 | yes | down |
| ENSRNOG00000050016 | Gtf3a | 1.701 | 0.766598364 | 0.000471747 | 0.016923298 | yes | up |
| ENSRNOG00000019470 | Hao2 | 0.616 | -0.699790822 | 0.000473765 | 0.016950103 | yes | down |
| ENSRNOG00000012458 | Cyp2e1 | 0.411 | -1.2828551 | 0.000475704 | 0.016973989 | yes | down |
| ENSRNOG00000013291 | Cyp2c23 | 0.61 | -0.714119511 | 0.000483137 | 0.017193236 | yes | down |
| ENSRNOG00000031163 | Nfkbiz | 0.447 | -1.160702037 | 0.000487413 | 0.017299273 | yes | down |
| ENSRNOG00000028629 | Akt1 | 2.025 | 1.017964665 | 0.000489044 | 0.017311134 | yes | up |
| ENSRNOG00000018969 | Gpatch4 | 2.056 | 1.039723973 | 0.000493788 | 0.017432815 | yes | up |
| ENSRNOG00000046955 | Cbx6 | 0.512 | -0.965666735 | 0.000504529 | 0.017765005 | yes | down |
| ENSRNOG00000047933 | Phykpl | 1.817 | 0.861465896 | 0.000506583 | 0.017790395 | yes | up |
| ENSRNOG00000007088 | Xpot | 1.995 | 0.996335707 | 0.000513001 | 0.017968494 | yes | up |
| ENSRNOG00000001770 | Ehhadh | 0.343 | -1.544280797 | 0.000517189 | 0.018067751 | yes | down |
| ENSRNOG00000009963 | Ctps1 | 2.394 | 1.259278772 | 0.000520034 | 0.018087936 | yes | up |
| ENSRNOG00000019413 | Atg16l2 | 0.519 | -0.947184135 | 0.000520477 | 0.018087936 | yes | down |
| ENSRNOG00000002667 | Lamc2 | 0.295 | -1.762053858 | 0.000522189 | 0.018100297 | yes | down |
| ENSRNOG00000021010 | Arl2 | 1.727 | 0.787906116 | 0.000526133 | 0.018154136 | yes | up |
| ENSRNOG00000000814 | Fabp7 | 0.258 | -1.952728196 | 0.000526463 | 0.018154136 | yes | down |
| ENSRNOG00000011140 | Prxl2a | 1.686 | 0.753816118 | 0.0005333 | 0.01824842 | yes | up |
| ENSRNOG00000046947 | Ak5 | 4.078 | 2.027966323 | 0.000541812 | 0.018492286 | yes | up |
| ENSRNOG00000006947 | Pdhx | 2.269 | 1.182039359 | 0.000544011 | 0.018519952 | yes | up |
| ENSRNOG00000010350 | Rcan2 | 0.407 | -1.295461378 | 0.000547998 | 0.018608229 | yes | down |
| ENSRNOG00000012960 | Uap1l1 | 0.451 | -1.147424048 | 0.000550215 | 0.018636077 | yes | down |
| ENSRNOG00000021424 | Cd300lf | 2.216 | 1.147768983 | 0.000564899 | 0.019036826 | yes | up |
| ENSRNOG00000023546 | Hspb1 | 0.218 | -2.197385454 | 0.000567739 | 0.019084335 | yes | down |
| ENSRNOG00000001847 | Ccdc91 | 1.574 | 0.654890149 | 0.000584822 | 0.01956003 | yes | up |
| ENSRNOG00000008843 | Eci1 | 0.349 | -1.517101555 | 0.000588723 | 0.01964126 | yes | down |
| ENSRNOG00000017120 | Abhd2 | 0.426 | -1.231125842 | 0.000594873 | 0.019796958 | yes | down |
| ENSRNOG00000055647 | Rbfa | 1.559 | 0.640606303 | 0.000602527 | 0.019959256 | yes | up |
| ENSRNOG00000009730 | Cyp7b1 | 0.469 | -1.093480738 | 0.000602741 | 0.019959256 | yes | down |
| ENSRNOG00000021176 | Mtmr11 | 0.299 | -1.739588145 | 0.000605051 | 0.019986138 | yes | down |
| ENSRNOG00000009589 | Angptl7 | 46.726 | 5.546143611 | 0.000616348 | 0.020309041 | yes | up |
| ENSRNOG00000036571 | Ces2c | 0.5 | -1.000528855 | 0.000631602 | 0.020725656 | yes | down |
| ENSRNOG00000020384 | Fam13b | 0.666 | -0.58603499 | 0.000632098 | 0.020725656 | yes | down |
| ENSRNOG00000000184 | Tmprss6 | 1.864 | 0.898400104 | 0.000636606 | 0.020822326 | yes | up |
| ENSRNOG00000030328 | Fam98a | 1.538 | 0.620614597 | 0.000640003 | 0.020882241 | yes | up |
| ENSRNOG00000004503 | L3hypdh | 1.929 | 0.947854287 | 0.000647704 | 0.021081984 | yes | up |
| ENSRNOG00000019485 | Bckdk | 2.145 | 1.101309636 | 0.000651651 | 0.021158827 | yes | up |
| ENSRNOG00000017469 | Anxa1 | 0.374 | -1.419383343 | 0.000655067 | 0.021218116 | yes | down |
| ENSRNOG00000016551 | Bcl2l11 | 0.51 | -0.971719262 | 0.000659975 | 0.021325348 | yes | down |
| ENSRNOG00000007462 | Septin8 | 0.49 | -1.028050769 | 0.000672694 | 0.021631574 | yes | down |
| ENSRNOG00000009949 | Pcdh18 | 0.537 | -0.897345743 | 0.000682163 | 0.021883324 | yes | down |
| ENSRNOG00000007607 | Nr4a1 | 0.274 | -1.865893679 | 0.000693757 | 0.022201896 | yes | down |
| ENSRNOG00000002775 | Npl | 0.438 | -1.192453103 | 0.00069738 | 0.022203327 | yes | down |
| ENSRNOG00000016516 | Mbp | 2.596 | 1.376072025 | 0.000698565 | 0.022203327 | yes | up |
| ENSRNOG00000006709 | Pzp | 0.606 | -0.721735511 | 0.000698793 | 0.022203327 | yes | down |
| ENSRNOG00000003148 | Timp2 | 0.558 | -0.8421914 | 0.000704155 | 0.022270085 | yes | down |
| ENSRNOG00000037638 | Timm50 | 1.588 | 0.667497007 | 0.000704232 | 0.022270085 | yes | up |
| ENSRNOG00000042319 | AABR07065814.2 | 0.193 | -2.372188529 | 0.000712976 | 0.022377262 | yes | down |
| ENSRNOG00000008346 | Itgb6 | 0.179 | -2.481158798 | 0.000713361 | 0.022377262 | yes | down |
| ENSRNOG00000022871 | LOC691170 | 0.509 | -0.973819393 | 0.000714328 | 0.022377262 | yes | down |
| ENSRNOG00000049385 | Adamtsl4 | 0.618 | -0.693559622 | 0.000722672 | 0.022480316 | yes | down |
| ENSRNOG00000013816 | Cmas | 1.524 | 0.608332737 | 0.000736491 | 0.022856921 | yes | up |
| ENSRNOG00000046601 | Unc5cl | 0.401 | -1.3176779 | 0.000740026 | 0.022907671 | yes | down |
| ENSRNOG00000051450 | Abracl | 0.396 | -1.336641387 | 0.000741615 | 0.022907671 | yes | down |
| ENSRNOG00000010224 | Rab30 | 0.303 | -1.724664876 | 0.000743276 | 0.022907671 | yes | down |
| ENSRNOG00000021380 | Fads6 | 0.637 | -0.650488755 | 0.000749114 | 0.022995788 | yes | down |
| ENSRNOG00000010468 | Elovl6 | 19.655 | 4.296805518 | 0.000749582 | 0.022995788 | yes | up |
| ENSRNOG00000018761 | Eif3c | 1.788 | 0.838159681 | 0.000752842 | 0.023042829 | yes | up |
| ENSRNOG00000031707 | Tuba3a | 0.265 | -1.918472724 | 0.000773379 | 0.023597165 | yes | down |
| ENSRNOG00000010695 | Pdgfc | 0.497 | -1.007477612 | 0.000774489 | 0.023597165 | yes | down |
| ENSRNOG00000049244 | Sepsecs | 1.74 | 0.799218287 | 0.000782513 | 0.02378732 | yes | up |
| ENSRNOG00000016896 | Rpl3 | 1.825 | 0.867652147 | 0.000788096 | 0.023902598 | yes | up |
| ENSRNOG00000011421 | Smap2 | 1.6 | 0.677844503 | 0.000791388 | 0.023922611 | yes | up |
| ENSRNOG00000001189 | Sik1 | 0.487 | -1.038660757 | 0.000792476 | 0.023922611 | yes | down |
| ENSRNOG00000019971 | Kxd1 | 1.803 | 0.850629835 | 0.000794134 | 0.023922611 | yes | up |
| ENSRNOG00000028116 | LOC102552988 | 2.444 | 1.289417537 | 0.000799098 | 0.02401794 | yes | up |
| ENSRNOG00000056246 | Gls | 0.572 | -0.806962051 | 0.000803425 | 0.024044483 | yes | down |
| ENSRNOG00000050539 | Fbln5 | 0.402 | -1.314272679 | 0.000804307 | 0.024044483 | yes | down |
| ENSRNOG00000000923 | Cct6a | 1.603 | 0.680588368 | 0.000811471 | 0.02417206 | yes | up |
| ENSRNOG00000010291 | Slc46a1 | 1.862 | 0.897050949 | 0.000815716 | 0.02424438 | yes | up |
| ENSRNOG00000014454 | Ap1m1 | 1.802 | 0.849994057 | 0.000834246 | 0.024740043 | yes | up |
| ENSRNOG00000007327 | Pars2 | 1.852 | 0.889238734 | 0.000841757 | 0.024904617 | yes | up |
| ENSRNOG00000022769 | Sp100 | 0.603 | -0.72889864 | 0.000843528 | 0.024904617 | yes | down |
| ENSRNOG00000054724 | Pycr3 | 1.65 | 0.722225995 | 0.000846916 | 0.024936496 | yes | up |
| ENSRNOG00000017326 | Ctbp2 | 0.471 | -1.085976798 | 0.000850749 | 0.024952178 | yes | down |
| ENSRNOG00000008586 | Aldh1l2 | 2.602 | 1.379376549 | 0.000859402 | 0.02509895 | yes | up |
| ENSRNOG00000002418 | Tgfb2 | 0.372 | -1.427683541 | 0.000863508 | 0.02509895 | yes | down |
| ENSRNOG00000015124 | Gpam | 3.041 | 1.604676875 | 0.000863522 | 0.02509895 | yes | up |
| ENSRNOG00000014605 | Lig4 | 0.512 | -0.966688996 | 0.000864174 | 0.02509895 | yes | down |
| ENSRNOG00000010277 | Idh3a | 2.051 | 1.036572066 | 0.000865157 | 0.02509895 | yes | up |
| ENSRNOG00000006963 | Ctse | 0.421 | -1.249340146 | 0.000868425 | 0.025139121 | yes | down |
| ENSRNOG00000025088 | Tmc3 | 1.973 | 0.98015218 | 0.000870634 | 0.025148506 | yes | up |
| ENSRNOG00000011971 | C1s | 0.482 | -1.052172581 | 0.000909832 | 0.026223982 | yes | down |
| ENSRNOG00000022296 | Pnpla5 | 28.503 | 4.833033766 | 0.000913988 | 0.02628701 | yes | up |
| ENSRNOG00000025001 | Pcolce | 0.389 | -1.363316312 | 0.000918892 | 0.026371217 | yes | down |
| ENSRNOG00000020038 | Chpf | 0.483 | -1.049085846 | 0.00094874 | 0.027142629 | yes | down |
| ENSRNOG00000058742 | AABR07067023.1 | 0.212 | -2.235833274 | 0.000949839 | 0.027142629 | yes | down |
| ENSRNOG00000005930 | Nnmt | 0.508 | -0.978284063 | 0.000960268 | 0.027382008 | yes | down |
| ENSRNOG00000003496 | Tbc1d9 | 0.451 | -1.150249756 | 0.00098932 | 0.028090368 | yes | down |
| ENSRNOG00000001130 | Nos1 | 3.794 | 1.92356083 | 0.001002023 | 0.02839064 | yes | up |
| ENSRNOG00000007666 | Cacnb4 | 4.988 | 2.318594602 | 0.001007389 | 0.028482214 | yes | up |
| ENSRNOG00000011690 | Rmdn3 | 1.505 | 0.590058305 | 0.001009948 | 0.028489697 | yes | up |
| ENSRNOG00000000938 | Ran | 1.7 | 0.765531119 | 0.001018024 | 0.028489697 | yes | up |
| ENSRNOG00000011500 | Pou2af1 | 0.224 | -2.160357697 | 0.001019602 | 0.028489697 | yes | down |
| ENSRNOG00000038047 | Mt1 | 0.309 | -1.696615756 | 0.001020463 | 0.028489697 | yes | down |
| ENSRNOG00000003597 | Tuba4a | 1.75 | 0.807410528 | 0.001027671 | 0.028626316 | yes | up |
| ENSRNOG00000019689 | Vwf | 0.473 | -1.080846758 | 0.001029646 | 0.028626316 | yes | down |
| ENSRNOG00000052668 | Tcf24 | 0.344 | -1.538149111 | 0.001041917 | 0.028907238 | yes | down |
| ENSRNOG00000018655 | Adsl | 1.594 | 0.672994546 | 0.001060326 | 0.029356963 | yes | up |
| ENSRNOG00000028236 | RGD1309104 | 0.538 | -0.894011423 | 0.001073336 | 0.029655624 | yes | down |
| ENSRNOG00000049308 | Sfxn2 | 0.658 | -0.604823299 | 0.001077977 | 0.02972232 | yes | down |
| ENSRNOG00000003772 | Csrp2 | 0.457 | -1.129824793 | 0.001080224 | 0.029722872 | yes | down |
| ENSRNOG00000008432 | Slc22a5 | 0.577 | -0.793310637 | 0.001091878 | 0.029935344 | yes | down |
| ENSRNOG00000031506 | Ftl1 | 1.963 | 0.9732897 | 0.001092433 | 0.029935344 | yes | up |
| ENSRNOG00000054555 | AABR07044551.1 | 0.312 | -1.678553646 | 0.001095797 | 0.029966013 | yes | down |
| ENSRNOG00000036678 | Aspscr1 | 1.726 | 0.787644262 | 0.001111363 | 0.03032953 | yes | up |
| ENSRNOG00000008947 | Cpz | 0.369 | -1.439763737 | 0.00111993 | 0.030500961 | yes | down |
| ENSRNOG00000059057 | Shld2 | 1.647 | 0.720079642 | 0.001126193 | 0.030546828 | yes | up |
| ENSRNOG00000029535 | Nrbp2 | 0.526 | -0.926631956 | 0.001134817 | 0.030688839 | yes | down |
| ENSRNOG00000019811 | LOC100362432 | 1.543 | 0.625731092 | 0.001136027 | 0.030688839 | yes | up |
| ENSRNOG00000007027 | Hgf | 0.589 | -0.763270949 | 0.001189755 | 0.032010635 | yes | down |
| ENSRNOG00000025930 | Chst13 | 2.008 | 1.005511394 | 0.001193143 | 0.03201218 | yes | up |
| ENSRNOG00000055909 | Apoa4 | 1.62 | 0.696381411 | 0.001218178 | 0.032469657 | yes | up |
| ENSRNOG00000026748 | Dennd2a | 0.562 | -0.832392987 | 0.001218981 | 0.032469657 | yes | down |
| ENSRNOG00000058393 | Taf15 | 1.559 | 0.640985453 | 0.001233737 | 0.032797261 | yes | up |
| ENSRNOG00000004048 | Lrrk2 | 0.486 | -1.039852201 | 0.001237642 | 0.032835664 | yes | down |
| ENSRNOG00000005890 | Serbp1 | 1.598 | 0.676420219 | 0.001245364 | 0.032974952 | yes | up |
| ENSRNOG00000057214 | AABR07043510.1 | 3.22 | 1.686923124 | 0.001251438 | 0.032987755 | yes | up |
| ENSRNOG00000043503 | Ehd1 | 1.79 | 0.839605528 | 0.001252916 | 0.032987755 | yes | up |
| ENSRNOG00000031031 | Zfp292 | 0.549 | -0.865679447 | 0.001253263 | 0.032987755 | yes | down |
| ENSRNOG00000033824 | Gpd2 | 1.761 | 0.816637491 | 0.00126536 | 0.033240604 | yes | up |
| ENSRNOG00000025689 | Abhd1 | 0.509 | -0.975244408 | 0.001275601 | 0.033301429 | yes | down |
| ENSRNOG00000051912 | Acnat2 | 0.297 | -1.750872304 | 0.001275905 | 0.033301429 | yes | down |
| ENSRNOG00000003895 | Rgs1 | 0.259 | -1.949544109 | 0.001276348 | 0.033301429 | yes | down |
| ENSRNOG00000009084 | Rpusd3 | 1.703 | 0.767888792 | 0.001277657 | 0.033301429 | yes | up |
| ENSRNOG00000059900 | Bst2 | 2.054 | 1.038570596 | 0.001289621 | 0.033429475 | yes | up |
| ENSRNOG00000019721 | Ypel3 | 0.493 | -1.020471162 | 0.00129259 | 0.033429475 | yes | down |
| ENSRNOG00000016265 | Acsl5 | 1.762 | 0.816816543 | 0.001310959 | 0.033838974 | yes | up |
| ENSRNOG00000020451 | Cd81 | 1.502 | 0.586572571 | 0.001314567 | 0.033866598 | yes | up |
| ENSRNOG00000061403 | AABR07039446.2 | 0.491 | -1.025209366 | 0.001319994 | 0.033920638 | yes | down |
| ENSRNOG00000019778 | Cavin1 | 0.544 | -0.878101857 | 0.001321748 | 0.033920638 | yes | down |
| ENSRNOG00000002194 | Coq2 | 1.571 | 0.652041245 | 0.001332121 | 0.034121224 | yes | up |
| ENSRNOG00000059504 | AABR07015078.2 | 0.371 | -1.430247389 | 0.001342259 | 0.034315039 | yes | down |
| ENSRNOG00000028390 | Hhipl1 | 6.148 | 2.620103627 | 0.0013475 | 0.034383159 | yes | up |
| ENSRNOG00000045991 | LOC100911558 | 0.496 | -1.010175786 | 0.001364476 | 0.03468367 | yes | down |
| ENSRNOG00000034203 | AABR07064312.1 | 0.451 | -1.148996685 | 0.001373104 | 0.034836648 | yes | down |
| ENSRNOG00000001712 | Alg3 | 1.669 | 0.738836804 | 0.001389649 | 0.035136621 | yes | up |
| ENSRNOG00000046283 | Tmem41a | 1.901 | 0.926500964 | 0.001390194 | 0.035136621 | yes | up |
| ENSRNOG00000049911 | LOC102556347 | 2.124 | 1.086806763 | 0.001413862 | 0.035561081 | yes | up |
| ENSRNOG00000003827 | Wipi1 | 0.426 | -1.231362356 | 0.001415042 | 0.035561081 | yes | down |
| ENSRNOG00000030154 | Cyp4a2 | 0.361 | -1.470845331 | 0.001415729 | 0.035561081 | yes | down |
| ENSRNOG00000021725 | Unc119b | 1.509 | 0.59387936 | 0.001422874 | 0.035625243 | yes | up |
| ENSRNOG00000051743 | AABR07043822.1 | 0.501 | -0.996035734 | 0.001446754 | 0.036097753 | yes | down |
| ENSRNOG00000024128 | Aco2 | 1.736 | 0.795754774 | 0.001449106 | 0.036097753 | yes | up |
| ENSRNOG00000054890 | Flna | 0.6 | -0.737374911 | 0.001449861 | 0.036097753 | yes | down |
| ENSRNOG00000036913 | Resf1 | 0.589 | -0.762842926 | 0.001454952 | 0.03611311 | yes | down |
| ENSRNOG00000011039 | Gch1 | 1.59 | 0.668837241 | 0.0014585 | 0.03611311 | yes | up |
| ENSRNOG00000027229 | Slc35d2 | 1.547 | 0.629740025 | 0.001460742 | 0.03611311 | yes | up |
| ENSRNOG00000051079 | AC095947.4 | 0.156 | -2.679287579 | 0.001463796 | 0.03611311 | yes | down |
| ENSRNOG00000056524 | Abcd1 | 1.722 | 0.78380035 | 0.001464008 | 0.03611311 | yes | up |
| ENSRNOG00000027784 | Tsku | 2.395 | 1.260053817 | 0.001472187 | 0.036181086 | yes | up |
| ENSRNOG00000059519 | Bbox1 | 1.672 | 0.741729315 | 0.001475666 | 0.036199942 | yes | up |
| ENSRNOG00000025269 | Slc25a44 | 1.909 | 0.933088114 | 0.001507969 | 0.036924491 | yes | up |
| ENSRNOG00000045844 | Impact | 0.534 | -0.904672584 | 0.001512251 | 0.036961509 | yes | down |
| ENSRNOG00000050404 | Pmepa1 | 0.303 | -1.721494244 | 0.001530541 | 0.037272034 | yes | down |
| ENSRNOG00000037839 | P2ry10 | 0.353 | -1.501898325 | 0.001533899 | 0.037285758 | yes | down |
| ENSRNOG00000000433 | Prrt1 | 0.26 | -1.94603864 | 0.001547513 | 0.037548296 | yes | down |
| ENSRNOG00000020497 | Plekha1 | 2.11 | 1.077173907 | 0.00157381 | 0.038117051 | yes | up |
| ENSRNOG00000014918 | Klrg1 | 0.347 | -1.527075087 | 0.00157882 | 0.038169112 | yes | down |
| ENSRNOG00000038331 | Akr1c1 | 0.389 | -1.362238943 | 0.001595341 | 0.038369598 | yes | down |
| ENSRNOG00000013358 | Aqp11 | 2.336 | 1.223811076 | 0.001595738 | 0.038369598 | yes | up |
| ENSRNOG00000059801 | Lima1 | 2.299 | 1.200953766 | 0.001598777 | 0.038373516 | yes | up |
| ENSRNOG00000002273 | Naaa | 0.441 | -1.179521858 | 0.001614008 | 0.038645654 | yes | down |
| ENSRNOG00000054391 | Snurf | 4.202 | 2.07095648 | 0.001625298 | 0.038645654 | yes | up |
| ENSRNOG00000027024 | Rgs16 | 11.439 | 3.515833062 | 0.001629083 | 0.038645654 | yes | up |
| ENSRNOG00000055078 | Cyp4b1 | 0.586 | -0.770310935 | 0.001630386 | 0.038645654 | yes | down |
| ENSRNOG00000002986 | Efhc2 | 20.34 | 4.346232028 | 0.001637174 | 0.038725994 | yes | up |
| ENSRNOG00000000043 | Idua | 0.533 | -0.906721774 | 0.001639579 | 0.038725994 | yes | down |
| ENSRNOG00000006204 | Slc30a3 | 0.37 | -1.435868928 | 0.001650484 | 0.038846057 | yes | down |
| ENSRNOG00000010830 | Slc25a4 | 0.48 | -1.058717757 | 0.001657534 | 0.038943295 | yes | down |
| ENSRNOG00000051056 | AABR07063279.1 | 2.651 | 1.406628968 | 0.001675912 | 0.039305887 | yes | up |
| ENSRNOG00000021006 | Tmem147 | 1.845 | 0.883782742 | 0.001682116 | 0.039347319 | yes | up |
| ENSRNOG00000055956 | AABR07015078.1 | 0.385 | -1.377836358 | 0.001683576 | 0.039347319 | yes | down |
| ENSRNOG00000003463 | Srebf1 | 3.442 | 1.783432316 | 0.001692029 | 0.039475749 | yes | up |
| ENSRNOG00000015035 | Myo7b | 2.176 | 1.121763071 | 0.001696947 | 0.039521403 | yes | up |
| ENSRNOG00000027742 | Adamtsl2 | 0.396 | -1.335880071 | 0.001726503 | 0.040007665 | yes | down |
| ENSRNOG00000021103 | Nrxn2 | 0.611 | -0.709931673 | 0.001728723 | 0.040007665 | yes | down |
| ENSRNOG00000058562 | AABR07065651.8 | 0.09 | -3.479688466 | 0.001729818 | 0.040007665 | yes | down |
| ENSRNOG00000012574 | Pum3 | 1.622 | 0.697730808 | 0.001749766 | 0.040312571 | yes | up |
| ENSRNOG00000002035 | Paqr3 | 2.177 | 1.122112532 | 0.001750513 | 0.040312571 | yes | up |
| ENSRNOG00000020300 | Lsp1 | 0.452 | -1.146450386 | 0.001752064 | 0.040312571 | yes | down |
| ENSRNOG00000011636 | Dennd6a | 0.651 | -0.619594108 | 0.00178185 | 0.040927347 | yes | down |
| ENSRNOG00000015236 | Mybbp1a | 1.832 | 0.873752055 | 0.00179363 | 0.041024712 | yes | up |
| ENSRNOG00000006873 | Dnase1 | 7.082 | 2.824165531 | 0.001794723 | 0.041024712 | yes | up |
| ENSRNOG00000026902 | Lyve1 | 0.561 | -0.833359597 | 0.001795311 | 0.041024712 | yes | down |
| ENSRNOG00000015125 | LOC100911548 | 0.493 | -1.019887398 | 0.001802818 | 0.04112583 | yes | down |
| ENSRNOG00000049121 | Bysl | 1.616 | 0.692782694 | 0.001813887 | 0.041307709 | yes | up |
| ENSRNOG00000032381 | Naglu | 1.552 | 0.633849833 | 0.001845475 | 0.041884132 | yes | up |
| ENSRNOG00000018536 | Pck2 | 2.434 | 1.283095847 | 0.001858945 | 0.042118203 | yes | up |
| ENSRNOG00000016538 | Itga8 | 0.4 | -1.322855106 | 0.001872867 | 0.042304751 | yes | down |
| ENSRNOG00000022354 | Pusl1 | 1.777 | 0.829768852 | 0.001873519 | 0.042304751 | yes | up |
| ENSRNOG00000002636 | Abat | 1.527 | 0.610690418 | 0.001879106 | 0.042317385 | yes | up |
| ENSRNOG00000004003 | Dusp10 | 0.301 | -1.732992381 | 0.001882035 | 0.042317385 | yes | down |
| ENSRNOG00000055295 | Pecr | 1.554 | 0.635797348 | 0.001883591 | 0.042317385 | yes | up |
| ENSRNOG00000024201 | Fer1l5 | 0.283 | -1.820812615 | 0.001902173 | 0.04259145 | yes | down |
| ENSRNOG00000025539 | Vps13a | 1.907 | 0.931034425 | 0.001923516 | 0.042997179 | yes | up |
| ENSRNOG00000030486 | Prdm6 | 0.209 | -2.257831166 | 0.001928421 | 0.043034753 | yes | down |
| ENSRNOG00000011024 | Zdhhc20 | 0.615 | -0.70067143 | 0.00193484 | 0.043105915 | yes | down |
| ENSRNOG00000053753 | AABR07070307.1 | 0.185 | -2.43541729 | 0.00194587 | 0.04327939 | yes | down |
| ENSRNOG00000048834 | Plin3 | 1.815 | 0.860272281 | 0.002018077 | 0.044736283 | yes | up |
| ENSRNOG00000006762 | Snu13 | 1.568 | 0.64864439 | 0.002029363 | 0.044845681 | yes | up |
| ENSRNOG00000006444 | Fkbp4 | 1.538 | 0.621054337 | 0.002032093 | 0.044845681 | yes | up |
| ENSRNOG00000015346 | Obsl1 | 0.355 | -1.492369317 | 0.002034955 | 0.044845681 | yes | down |
| ENSRNOG00000008570 | Cadps | 11.222 | 3.488239666 | 0.002036454 | 0.044845681 | yes | up |
| ENSRNOG00000014166 | Smoc2 | 0.388 | -1.366233143 | 0.002074853 | 0.045599288 | yes | down |
| ENSRNOG00000008996 | Dpysl5 | 6.081 | 2.604260224 | 0.00207751 | 0.045599288 | yes | up |
| ENSRNOG00000007197 | Nr1h4 | 1.798 | 0.846240017 | 0.002085005 | 0.045654637 | yes | up |
| ENSRNOG00000014371 | Cdh13 | 0.396 | -1.336559674 | 0.002086874 | 0.045654637 | yes | down |
| ENSRNOG00000016478 | Eif5a | 1.676 | 0.744666934 | 0.002094108 | 0.045682008 | yes | up |
| ENSRNOG00000018184 | Tpm1 | 1.698 | 0.763763448 | 0.002096283 | 0.045682008 | yes | up |
| ENSRNOG00000003136 | Fcrla | 0.284 | -1.818347494 | 0.002099106 | 0.045682008 | yes | down |
| ENSRNOG00000004032 | Tmem94 | 1.773 | 0.82597977 | 0.002102058 | 0.045682008 | yes | up |
| ENSRNOG00000013917 | Igsf10 | 0.538 | -0.893567605 | 0.002105241 | 0.045682008 | yes | down |
| ENSRNOG00000018404 | Aars | 1.607 | 0.684527252 | 0.002145844 | 0.04648748 | yes | up |
| ENSRNOG00000052564 | Gpx3 | 0.431 | -1.215451945 | 0.002167843 | 0.046860678 | yes | down |
| ENSRNOG00000004330 | Chrdl1 | 0.214 | -2.225406333 | 0.002170094 | 0.046860678 | yes | down |
| ENSRNOG00000004180 | Tafa2 | 0.381 | -1.391866357 | 0.002188062 | 0.047140762 | yes | down |
| ENSRNOG00000019854 | Napsa | 0.453 | -1.142247846 | 0.002200668 | 0.047291325 | yes | down |
| ENSRNOG00000015089 | Mcoln2 | 5.215 | 2.382535202 | 0.002208078 | 0.047374279 | yes | up |
| ENSRNOG00000010164 | Mrps2 | 1.633 | 0.707170447 | 0.002225343 | 0.047622276 | yes | up |
| ENSRNOG00000028460 | Lrrc8e | 0.192 | -2.378390096 | 0.002228341 | 0.047622276 | yes | down |
| ENSRNOG00000013445 | Aaas | 1.611 | 0.687989324 | 0.002230343 | 0.047622276 | yes | up |
| ENSRNOG00000050675 | Myl4 | 4.171 | 2.060221586 | 0.00225461 | 0.048063522 | yes | up |
| ENSRNOG00000047100 | LOC367975 | 5.536 | 2.468963934 | 0.002272097 | 0.048359077 | yes | up |
| ENSRNOG00000019430 | Coro1a | 0.496 | -1.010945474 | 0.00229384 | 0.048744099 | yes | down |
| ENSRNOG00000007128 | Nop56 | 1.762 | 0.817379086 | 0.002301956 | 0.048838803 | yes | up |
| ENSRNOG00000020736 | Nadsyn1 | 1.503 | 0.587800963 | 0.00231219 | 0.04897806 | yes | up |
| ENSRNOG00000058003 | Spon1 | 0.281 | -1.829675877 | 0.002326016 | 0.049192846 | yes | down |
| ENSRNOG00000028219 | Tmem209 | 1.7 | 0.765600406 | 0.002337897 | 0.049258649 | yes | up |
| ENSRNOG00000003635 | Disp1 | 1.901 | 0.926797589 | 0.002340201 | 0.049258649 | yes | up |
| ENSRNOG00000024277 | AABR07035470.1 | 0.418 | -1.259238686 | 0.002346081 | 0.049304644 | yes | down |
| ENSRNOG00000000588 | Slc16a10 | 0.335 | -1.577065068 | 0.00235523 | 0.049419094 | yes | down |
| ENSRNOG00000059750 | AC141102.1 | 0.393 | -1.345908537 | 0.002366765 | 0.049519592 | yes | down |
| ENSRNOG00000004500 | Myc | 2.858 | 1.514857884 | 0.002367441 | 0.049519592 | yes | up |
| ENSRNOG00000059968 | Jak2 | 0.6 | -0.737373872 | 0.002373329 | 0.049565053 | yes | down |
| ENSRNOG00000019073 | Ikbkb | 1.545 | 0.627797497 | 0.002384231 | 0.049714949 | yes | up |
| ENSRNOG00000010128 | Slc27a2 | 0.568 | -0.815778934 | 0.002391362 | 0.049758362 | yes | down |
| ENSRNOG00000016182 | Tgfa | 2.103 | 1.072762439 | 0.002393771 | 0.049758362 | yes | up |

**Table S2|** Differently expressed genes (BAL vs FRU). FC: fold change; FRU: fructose-induced group rats; BAL; low dose of baicalein treatment.

| Gene ID | Gene name | FC(BAL/FRU) | Log_2_FC(BAL/FRU) | p-value | p-adjust | Significant | Regulate |
| --- | --- | --- | --- | --- | --- | --- | --- |
| ENSRNOG00000016957 | Igfbp2 | 13.294 | 3.732701746 | 5.90672E-14 | 6.67164E-10 | yes | up |
| ENSRNOG00000051232 | Gbe1 | 0.438 | -1.189845632 | 3.29264E-12 | 1.85952E-08 | yes | down |
| ENSRNOG00000036604 | Ifit2 | 0.294 | -1.76784834 | 1.13122E-10 | 4.25904E-07 | yes | down |
| ENSRNOG00000007219 | LOC103692716 | 0.266 | -1.910196371 | 5.68001E-10 | 1.60389E-06 | yes | down |
| ENSRNOG00000021380 | Fads6 | 1.785 | 0.835816577 | 6.98817E-09 | 1.48241E-05 | yes | up |
| ENSRNOG00000058847 | AABR07044001.4 | 4.235 | 2.082357137 | 7.87469E-09 | 1.48241E-05 | yes | up |
| ENSRNOG00000018345 | Abce1 | 0.513 | -0.962791582 | 1.51216E-08 | 2.43998E-05 | yes | down |
| ENSRNOG00000059586 | AABR07015080.2 | 3.258 | 1.703989827 | 1.89571E-08 | 2.51917E-05 | yes | up |
| ENSRNOG00000000900 | Tpst1 | 1.903 | 0.928361632 | 2.0073E-08 | 2.51917E-05 | yes | up |
| ENSRNOG00000003897 | Col1a1 | 2.724 | 1.445876936 | 2.29579E-08 | 2.59309E-05 | yes | up |
| ENSRNOG00000019689 | Vwf | 2.027 | 1.019575024 | 4.63679E-08 | 4.76114E-05 | yes | up |
| ENSRNOG00000003743 | Dars | 0.461 | -1.11676588 | 7.42841E-08 | 6.99199E-05 | yes | down |
| ENSRNOG00000033697 | Casp4 | 0.271 | -1.885024646 | 8.97351E-08 | 7.7966E-05 | yes | down |
| ENSRNOG00000010519 | Cyp39a1 | 0.544 | -0.878834209 | 1.38686E-07 | 0.000108752 | yes | down |
| ENSRNOG00000059468 | AABR07034573.3 | 0.157 | -2.669758299 | 1.47152E-07 | 0.000108752 | yes | down |
| ENSRNOG00000014387 | Chac1 | 0.198 | -2.333497566 | 1.54054E-07 | 0.000108752 | yes | down |
| ENSRNOG00000007506 | Ndufaf4 | 0.349 | -1.519954839 | 1.76232E-07 | 0.000117091 | yes | down |
| ENSRNOG00000020853 | Scap | 1.755 | 0.811096131 | 1.89471E-07 | 0.000118893 | yes | up |
| ENSRNOG00000031203 | Scfd1 | 0.4 | -1.32359401 | 2.90958E-07 | 0.000172967 | yes | down |
| ENSRNOG00000020342 | Samd11 | 2.123 | 1.085964285 | 3.5378E-07 | 0.000188203 | yes | up |
| ENSRNOG00000020573 | Efna1 | 1.963 | 0.973194619 | 3.64805E-07 | 0.000188203 | yes | up |
| ENSRNOG00000009484 | Ptcd3 | 0.518 | -0.948831415 | 3.66575E-07 | 0.000188203 | yes | down |
| ENSRNOG00000013291 | Cyp2c23 | 2.107 | 1.07507541 | 5.61697E-07 | 0.000275842 | yes | up |
| ENSRNOG00000017447 | Eif2s2 | 0.443 | -1.173492952 | 6.42025E-07 | 0.000296957 | yes | down |
| ENSRNOG00000014525 | Hspd1 | 0.528 | -0.921161181 | 6.57274E-07 | 0.000296957 | yes | down |
| ENSRNOG00000010210 | Slc7a11 | 0.031 | -5.016565881 | 7.01162E-07 | 0.000304601 | yes | down |
| ENSRNOG00000000546 | Nt5dc1 | 0.537 | -0.898096757 | 7.86417E-07 | 0.000318218 | yes | down |
| ENSRNOG00000010887 | RGD1309534 | 0.493 | -1.019138381 | 7.88853E-07 | 0.000318218 | yes | down |
| ENSRNOG00000017120 | Abhd2 | 2.583 | 1.368959313 | 8.9262E-07 | 0.00034766 | yes | up |
| ENSRNOG00000059504 | AABR07015078.2 | 3.691 | 1.883968924 | 1.20933E-06 | 0.000449255 | yes | up |
| ENSRNOG00000007753 | Plaa | 0.564 | -0.8251807 | 1.23301E-06 | 0.000449255 | yes | down |
| ENSRNOG00000008331 | Washc4 | 0.472 | -1.083794796 | 1.36532E-06 | 0.000473617 | yes | down |
| ENSRNOG00000017510 | Mfge8 | 1.754 | 0.810882543 | 1.38374E-06 | 0.000473617 | yes | up |
| ENSRNOG00000025910 | Hccs | 0.485 | -1.044101117 | 1.537E-06 | 0.000510601 | yes | down |
| ENSRNOG00000004177 | Myo1a | 0.006 | -7.4282559 | 1.6695E-06 | 0.000535369 | yes | down |
| ENSRNOG00000003600 | Pnpt1 | 0.539 | -0.891561613 | 1.70635E-06 | 0.000535369 | yes | down |
| ENSRNOG00000006652 | Ddx1 | 0.59 | -0.760851021 | 1.80375E-06 | 0.000537456 | yes | down |
| ENSRNOG00000025459 | Mars1 | 0.539 | -0.892315065 | 1.80817E-06 | 0.000537456 | yes | down |
| ENSRNOG00000005374 | Lyar | 0.46 | -1.119550509 | 1.87192E-06 | 0.000542138 | yes | down |
| ENSRNOG00000009430 | Gnl2 | 0.56 | -0.836025423 | 2.28864E-06 | 0.000646255 | yes | down |
| ENSRNOG00000008754 | Flvcr2 | 2.646 | 1.404013312 | 2.65693E-06 | 0.000727578 | yes | up |
| ENSRNOG00000003746 | Gjb1 | 1.6 | 0.678296312 | 2.70547E-06 | 0.000727578 | yes | up |
| ENSRNOG00000027408 | Ppid | 0.409 | -1.288572669 | 2.82995E-06 | 0.000743355 | yes | down |
| ENSRNOG00000054901 | Rif1 | 0.341 | -1.550070994 | 3.20859E-06 | 0.000800064 | yes | down |
| ENSRNOG00000017473 | Ttc25 | 0.213 | -2.231272893 | 3.21673E-06 | 0.000800064 | yes | down |
| ENSRNOG00000020851 | Aplp1 | 1.866 | 0.900284682 | 3.25834E-06 | 0.000800064 | yes | up |
| ENSRNOG00000005266 | Amdhd1 | 2.161 | 1.111755094 | 3.38666E-06 | 0.00081388 | yes | up |
| ENSRNOG00000049994 | Ifi44l | 0.365 | -1.452287384 | 3.98018E-06 | 0.000936587 | yes | down |
| ENSRNOG00000019708 | Ctsf | 1.598 | 0.676582515 | 4.75226E-06 | 0.001095445 | yes | up |
| ENSRNOG00000007124 | Krcc1 | 0.48 | -1.059927616 | 4.85407E-06 | 0.001096535 | yes | down |
| ENSRNOG00000046621 | AABR07043748.1 | 0.154 | -2.701984939 | 5.50176E-06 | 0.001203691 | yes | down |
| ENSRNOG00000048194 | LOC100912380 | 1.683 | 0.751224348 | 5.64813E-06 | 0.001203691 | yes | up |
| ENSRNOG00000045747 | Capns1 | 1.683 | 0.751224348 | 5.64813E-06 | 0.001203691 | yes | up |
| ENSRNOG00000059519 | Bbox1 | 0.58 | -0.786945126 | 6.12446E-06 | 0.001281033 | yes | down |
| ENSRNOG00000007529 | Bmf | 1.966 | 0.975084712 | 7.09855E-06 | 0.001457783 | yes | up |
| ENSRNOG00000016348 | Tat | 2.825 | 1.498241705 | 7.39788E-06 | 0.001492125 | yes | up |
| ENSRNOG00000013971 | Psat1 | 0.219 | -2.189615113 | 7.97843E-06 | 0.001552366 | yes | down |
| ENSRNOG00000014191 | Zfp395 | 1.836 | 0.8766732 | 8.04395E-06 | 0.001552366 | yes | up |
| ENSRNOG00000001884 | Ranbp1 | 0.499 | -1.002672917 | 8.10886E-06 | 0.001552366 | yes | down |
| ENSRNOG00000000923 | Cct6a | 0.581 | -0.782303193 | 8.75251E-06 | 0.001647661 | yes | down |
| ENSRNOG00000001592 | Cct8 | 0.559 | -0.838598642 | 9.08975E-06 | 0.001682871 | yes | down |
| ENSRNOG00000002393 | Eprs | 0.515 | -0.957115041 | 9.23754E-06 | 0.001682871 | yes | down |
| ENSRNOG00000023657 | Gprin3 | 0.31 | -1.69001106 | 9.56927E-06 | 0.001715634 | yes | down |
| ENSRNOG00000012505 | Ppwd1 | 0.403 | -1.309970855 | 1.02665E-05 | 0.001792346 | yes | down |
| ENSRNOG00000010921 | Taf1d | 0.377 | -1.409087694 | 1.03145E-05 | 0.001792346 | yes | down |
| ENSRNOG00000018553 | Pitpnm1 | 2.021 | 1.015353699 | 1.24536E-05 | 0.002131271 | yes | up |
| ENSRNOG00000018304 | Lars1 | 0.502 | -0.993681521 | 1.33137E-05 | 0.002220775 | yes | down |
| ENSRNOG00000048949 | LOC102549542 | 0.186 | -2.42929848 | 1.33699E-05 | 0.002220775 | yes | down |
| ENSRNOG00000010027 | Atr | 0.415 | -1.26905639 | 1.37354E-05 | 0.002248423 | yes | down |
| ENSRNOG00000043077 | Zfpm1 | 1.866 | 0.899779491 | 1.50163E-05 | 0.002392391 | yes | up |
| ENSRNOG00000043223 | Zfp949 | 0.378 | -1.40341748 | 1.50385E-05 | 0.002392391 | yes | down |
| ENSRNOG00000018375 | Eif2b3 | 0.499 | -1.002337901 | 1.62877E-05 | 0.002555132 | yes | down |
| ENSRNOG00000007779 | Kank4 | 2.729 | 1.448616918 | 1.71763E-05 | 0.002657619 | yes | up |
| ENSRNOG00000018972 | Rab18 | 0.62 | -0.69005779 | 1.76502E-05 | 0.002665717 | yes | down |
| ENSRNOG00000015332 | Thoc1 | 0.532 | -0.90966047 | 1.79667E-05 | 0.002665717 | yes | down |
| ENSRNOG00000019525 | Hspa9 | 0.553 | -0.853443284 | 1.81105E-05 | 0.002665717 | yes | down |
| ENSRNOG00000055956 | AABR07015078.1 | 3.143 | 1.652175733 | 1.81964E-05 | 0.002665717 | yes | up |
| ENSRNOG00000016659 | Habp2 | 2.085 | 1.059784619 | 1.86797E-05 | 0.002665717 | yes | up |
| ENSRNOG00000013090 | Gadd45g | 0.206 | -2.27671916 | 1.88252E-05 | 0.002665717 | yes | down |
| ENSRNOG00000009117 | Otub2 | 0.309 | -1.695698085 | 1.8988E-05 | 0.002665717 | yes | down |
| ENSRNOG00000058251 | AABR07069728.1 | 0.39 | -1.359160538 | 1.91167E-05 | 0.002665717 | yes | down |
| ENSRNOG00000018021 | Brix1 | 0.471 | -1.085161991 | 1.96188E-05 | 0.002702366 | yes | down |
| ENSRNOG00000007152 | Bhlhe40 | 0.41 | -1.287083896 | 2.07458E-05 | 0.002823181 | yes | down |
| ENSRNOG00000042318 | Pglyrp2 | 1.715 | 0.778131322 | 2.28807E-05 | 0.003076643 | yes | up |
| ENSRNOG00000010427 | Ipo7 | 0.467 | -1.100032253 | 2.47655E-05 | 0.003290902 | yes | down |
| ENSRNOG00000030630 | Ehmt2 | 1.556 | 0.637393848 | 2.67091E-05 | 0.003507894 | yes | up |
| ENSRNOG00000047439 | Pot1b | 0.325 | -1.619532444 | 2.70764E-05 | 0.003515268 | yes | down |
| ENSRNOG00000029830 | Adm2 | 0.167 | -2.58498531 | 2.85582E-05 | 0.003665504 | yes | down |
| ENSRNOG00000007831 | Ufl1 | 0.583 | -0.777439199 | 2.95381E-05 | 0.00373305 | yes | down |
| ENSRNOG00000016486 | Nop58 | 0.353 | -1.501887149 | 2.97454E-05 | 0.00373305 | yes | down |
| ENSRNOG00000005615 | Gadd45a | 0.259 | -1.950112275 | 3.01773E-05 | 0.003742661 | yes | down |
| ENSRNOG00000046968 | Nol8 | 0.385 | -1.376290452 | 3.04847E-05 | 0.003742661 | yes | down |
| ENSRNOG00000014599 | Dhx36 | 0.561 | -0.835008919 | 3.10804E-05 | 0.003747016 | yes | down |
| ENSRNOG00000058478 | Mapk8ip1 | 2.127 | 1.088931476 | 3.11837E-05 | 0.003747016 | yes | up |
| ENSRNOG00000008079 | Ugp2 | 0.508 | -0.976531243 | 3.24615E-05 | 0.003859506 | yes | down |
| ENSRNOG00000012255 | Ktn1 | 0.514 | -0.960665673 | 3.37206E-05 | 0.003967438 | yes | down |
| ENSRNOG00000057101 | AABR07050652.1 | 0.145 | -2.785577728 | 3.48073E-05 | 0.00399575 | yes | down |
| ENSRNOG00000048577 | Zfp955a | 0.243 | -2.043131857 | 3.52032E-05 | 0.00399575 | yes | down |
| ENSRNOG00000060594 | LOC100911672 | 2.285 | 1.192253275 | 3.53208E-05 | 0.00399575 | yes | up |
| ENSRNOG00000020594 | Rhbdf1 | 2.113 | 1.079513296 | 3.53763E-05 | 0.00399575 | yes | up |
| ENSRNOG00000053410 | Adcy10 | 3.684 | 1.881385901 | 3.6111E-05 | 0.00403835 | yes | up |
| ENSRNOG00000010833 | Mthfd2 | 0.167 | -2.583219069 | 3.67664E-05 | 0.004071339 | yes | down |
| ENSRNOG00000018273 | Ncl | 0.484 | -1.047070091 | 4.12052E-05 | 0.004495744 | yes | down |
| ENSRNOG00000018730 | Nectin2 | 2.168 | 1.116235341 | 4.13951E-05 | 0.004495744 | yes | up |
| ENSRNOG00000006608 | Tmem70 | 0.566 | -0.820626552 | 4.18411E-05 | 0.004500904 | yes | down |
| ENSRNOG00000014981 | Usp14 | 0.607 | -0.720105865 | 4.41366E-05 | 0.004659719 | yes | down |
| ENSRNOG00000029698 | Pim3 | 2.634 | 1.397476553 | 4.41425E-05 | 0.004659719 | yes | up |
| ENSRNOG00000050545 | AABR07015056.1 | 2.798 | 1.484343702 | 4.49887E-05 | 0.004701821 | yes | up |
| ENSRNOG00000018606 | Olr59 | 0.406 | -1.299486904 | 4.53739E-05 | 0.004701821 | yes | down |
| ENSRNOG00000007895 | Pdhb | 0.542 | -0.882381118 | 4.6528E-05 | 0.004762458 | yes | down |
| ENSRNOG00000017133 | LOC306766 | 1.64 | 0.713513539 | 4.68024E-05 | 0.004762458 | yes | up |
| ENSRNOG00000016260 | Polr3g | 0.473 | -1.080059889 | 4.84567E-05 | 0.004886775 | yes | down |
| ENSRNOG00000007699 | Golga5 | 0.621 | -0.686789299 | 4.97996E-05 | 0.004936697 | yes | down |
| ENSRNOG00000000415 | Asf1a | 0.541 | -0.885110669 | 4.98259E-05 | 0.004936697 | yes | down |
| ENSRNOG00000011294 | Usp33 | 0.573 | -0.803713984 | 5.21611E-05 | 0.005123123 | yes | down |
| ENSRNOG00000012455 | Tardbp | 0.614 | -0.7037213 | 5.42859E-05 | 0.005285851 | yes | down |
| ENSRNOG00000000587 | Rpf2 | 0.396 | -1.336021529 | 5.48872E-05 | 0.005298724 | yes | down |
| ENSRNOG00000017091 | Slc25a46 | 0.547 | -0.871334543 | 5.57487E-05 | 0.005334406 | yes | down |
| ENSRNOG00000006221 | Uba3 | 0.589 | -0.762980004 | 5.62014E-05 | 0.005334406 | yes | down |
| ENSRNOG00000001720 | Hes1 | 1.931 | 0.949528193 | 5.81509E-05 | 0.005473452 | yes | up |
| ENSRNOG00000012392 | Dnajc2 | 0.498 | -1.005914468 | 5.86992E-05 | 0.005479404 | yes | down |
| ENSRNOG00000032776 | Srp54a | 0.555 | -0.85043201 | 5.92216E-05 | 0.005482852 | yes | down |
| ENSRNOG00000012564 | RGD1564541 | 0.528 | -0.922471793 | 6.19658E-05 | 0.005690274 | yes | down |
| ENSRNOG00000002871 | Rbm25l1 | 0.447 | -1.162375148 | 6.49516E-05 | 0.005914063 | yes | down |
| ENSRNOG00000010750 | Twistnb | 0.363 | -1.462337711 | 6.545E-05 | 0.005914063 | yes | down |
| ENSRNOG00000018390 | Pld3 | 1.578 | 0.657953479 | 6.66545E-05 | 0.005964205 | yes | up |
| ENSRNOG00000009949 | Pcdh18 | 1.978 | 0.984395761 | 6.7219E-05 | 0.005964205 | yes | up |
| ENSRNOG00000030633 | Lmf2 | 1.609 | 0.685841015 | 6.79908E-05 | 0.005964205 | yes | up |
| ENSRNOG00000013547 | Slc6a12 | 1.557 | 0.639057001 | 6.81171E-05 | 0.005964205 | yes | up |
| ENSRNOG00000018436 | Apoa5 | 1.767 | 0.821390688 | 6.86859E-05 | 0.005967748 | yes | up |
| ENSRNOG00000046257 | Ctdspl | 1.924 | 0.94414051 | 7.52899E-05 | 0.006451546 | yes | up |
| ENSRNOG00000005478 | Fkbp9 | 1.583 | 0.662446612 | 7.53965E-05 | 0.006451546 | yes | up |
| ENSRNOG00000006636 | Otud6b | 0.44 | -1.184013864 | 7.64575E-05 | 0.006493135 | yes | down |
| ENSRNOG00000015498 | Il17rb | 0.374 | -1.417658179 | 7.82462E-05 | 0.006595453 | yes | down |
| ENSRNOG00000002643 | Ugdh | 0.595 | -0.74948837 | 7.8982E-05 | 0.00660816 | yes | down |
| ENSRNOG00000011420 | Mtmr7 | 0.518 | -0.948305436 | 8.41194E-05 | 0.00698624 | yes | down |
| ENSRNOG00000022252 | Kbtbd3 | 0.426 | -1.229662866 | 8.55072E-05 | 0.007049665 | yes | down |
| ENSRNOG00000007446 | Apip | 0.437 | -1.194581733 | 8.66919E-05 | 0.007095543 | yes | down |
| ENSRNOG00000001066 | Itih2 | 1.504 | 0.589236146 | 8.80434E-05 | 0.007154317 | yes | up |
| ENSRNOG00000016021 | Lims2 | 1.581 | 0.660709971 | 9.35767E-05 | 0.007549637 | yes | up |
| ENSRNOG00000008626 | Manea | 0.422 | -1.244599944 | 9.67998E-05 | 0.007754281 | yes | down |
| ENSRNOG00000009935 | Xpo1 | 0.567 | -0.818469687 | 0.000100666 | 0.008007206 | yes | down |
| ENSRNOG00000008064 | Naga | 1.739 | 0.798257526 | 0.000104124 | 0.00822434 | yes | up |
| ENSRNOG00000003551 | B4galt3 | 1.714 | 0.777240678 | 0.000108057 | 0.008475724 | yes | up |
| ENSRNOG00000010170 | Tubb4b | 0.525 | -0.929712505 | 0.0001096 | 0.008537476 | yes | down |
| ENSRNOG00000016266 | Mphosph10 | 0.469 | -1.091243401 | 0.000114603 | 0.008866062 | yes | down |
| ENSRNOG00000050323 | Srsf1 | 0.59 | -0.762213146 | 0.000116044 | 0.008916463 | yes | down |
| ENSRNOG00000029152 | Tmem69 | 0.556 | -0.845658418 | 0.000119674 | 0.009071921 | yes | down |
| ENSRNOG00000037604 | Ascc3 | 0.611 | -0.710033733 | 0.000121545 | 0.009107153 | yes | down |
| ENSRNOG00000010237 | Map7d1 | 1.786 | 0.836738091 | 0.000121751 | 0.009107153 | yes | up |
| ENSRNOG00000002487 | Mbnl3 | 0.495 | -1.015388802 | 0.000122694 | 0.009117272 | yes | down |
| ENSRNOG00000056747 | AABR07015055.1 | 2.155 | 1.107411788 | 0.0001253 | 0.009250099 | yes | up |
| ENSRNOG00000003674 | Pir | 0.556 | -0.847225302 | 0.000126756 | 0.009296778 | yes | down |
| ENSRNOG00000012124 | Trappc13 | 0.575 | -0.798351068 | 0.00013281 | 0.009677985 | yes | down |
| ENSRNOG00000003712 | Ppp4r3b | 0.467 | -1.097395926 | 0.000137551 | 0.009858353 | yes | down |
| ENSRNOG00000005258 | Myef2 | 0.406 | -1.300881257 | 0.000137851 | 0.009858353 | yes | down |
| ENSRNOG00000031576 | Zfp455 | 0.43 | -1.217564379 | 0.000137903 | 0.009858353 | yes | down |
| ENSRNOG00000015969 | Rpf1 | 0.518 | -0.948296953 | 0.000139288 | 0.009894718 | yes | down |
| ENSRNOG00000009243 | Oaf | 2.106 | 1.074467627 | 0.000144858 | 0.010226041 | yes | up |
| ENSRNOG00000022054 | Paqr7 | 2.146 | 1.101762657 | 0.000147557 | 0.010351928 | yes | up |
| ENSRNOG00000019273 | Capn7 | 0.573 | -0.803898686 | 0.000149523 | 0.010425056 | yes | down |
| ENSRNOG00000014588 | Dbr1 | 0.541 | -0.885162161 | 0.000156804 | 0.010865635 | yes | down |
| ENSRNOG00000020035 | Cyp17a1 | 3.692 | 1.884516541 | 0.000159582 | 0.010990699 | yes | up |
| ENSRNOG00000017967 | Asb13 | 0.566 | -0.821719696 | 0.000166891 | 0.011379966 | yes | down |
| ENSRNOG00000031656 | Ccnh | 0.497 | -1.009027676 | 0.000167249 | 0.011379966 | yes | down |
| ENSRNOG00000060048 | Eif2s3y | 0.452 | -1.147161107 | 0.000174292 | 0.011689901 | yes | down |
| ENSRNOG00000003825 | Wdr75 | 0.546 | -0.872512667 | 0.000174763 | 0.011689901 | yes | down |
| ENSRNOG00000059057 | Shld2 | 0.54 | -0.888613231 | 0.000175184 | 0.011689901 | yes | down |
| ENSRNOG00000014702 | Elovl2 | 1.791 | 0.84052655 | 0.000175944 | 0.011689901 | yes | up |
| ENSRNOG00000011194 | Tpp2 | 0.583 | -0.778023047 | 0.000182481 | 0.012053372 | yes | down |
| ENSRNOG00000034177 | Efna5 | 1.929 | 0.948179288 | 0.000187058 | 0.012283841 | yes | up |
| ENSRNOG00000055082 | Maz | 1.502 | 0.586971082 | 0.000188157 | 0.012284569 | yes | up |
| ENSRNOG00000006432 | Trnt1 | 0.466 | -1.101877382 | 0.000189918 | 0.012295925 | yes | down |
| ENSRNOG00000010116 | Leo1 | 0.637 | -0.651198687 | 0.000190508 | 0.012295925 | yes | down |
| ENSRNOG00000022485 | Chuk | 0.613 | -0.707125852 | 0.000192244 | 0.012337488 | yes | down |
| ENSRNOG00000059702 | Tex9 | 0.419 | -1.254156581 | 0.000195263 | 0.01246045 | yes | down |
| ENSRNOG00000031421 | Eif1a | 0.451 | -1.148362473 | 0.000204429 | 0.012972045 | yes | down |
| ENSRNOG00000028036 | Adamts7 | 2.867 | 1.519686954 | 0.000211796 | 0.013364469 | yes | up |
| ENSRNOG00000060896 | AABR07063424.1 | 3.146 | 1.653453477 | 0.000218675 | 0.013721838 | yes | up |
| ENSRNOG00000026420 | Iqcg | 0.444 | -1.17220206 | 0.000220851 | 0.013781839 | yes | down |
| ENSRNOG00000025892 | Set | 0.591 | -0.757854287 | 0.000226039 | 0.014028065 | yes | down |
| ENSRNOG00000032436 | Tmod3 | 0.593 | -0.753713902 | 0.000228931 | 0.014129926 | yes | down |
| ENSRNOG00000002839 | Slc19a2 | 1.816 | 0.861030588 | 0.000234337 | 0.014384978 | yes | up |
| ENSRNOG00000013438 | Yae1 | 0.51 | -0.971114922 | 0.000236446 | 0.014435964 | yes | down |
| ENSRNOG00000004549 | Dhx40 | 0.577 | -0.792733848 | 0.000243235 | 0.014488265 | yes | down |
| ENSRNOG00000055154 | AABR07063462.1 | 2.96 | 1.565592598 | 0.000244676 | 0.014488265 | yes | up |
| ENSRNOG00000014945 | Kpna3 | 0.6 | -0.736948961 | 0.000244999 | 0.014488265 | yes | down |
| ENSRNOG00000058780 | Igfbp1 | 2.14 | 1.097291873 | 0.000245759 | 0.014488265 | yes | up |
| ENSRNOG00000039717 | Ipo11 | 0.559 | -0.839571684 | 0.000246113 | 0.014488265 | yes | down |
| ENSRNOG00000012385 | Naa15 | 0.443 | -1.174976564 | 0.000246374 | 0.014488265 | yes | down |
| ENSRNOG00000018059 | Ihh | 2.505 | 1.325087537 | 0.000247473 | 0.014488265 | yes | up |
| ENSRNOG00000012803 | Wdr61 | 0.642 | -0.639396066 | 0.000247564 | 0.014488265 | yes | down |
| ENSRNOG00000002207 | Guf1 | 0.53 | -0.915965456 | 0.000252657 | 0.014710103 | yes | down |
| ENSRNOG00000016753 | Slc14a1 | 0.166 | -2.588296523 | 0.000261558 | 0.015105783 | yes | down |
| ENSRNOG00000016596 | Hspa4 | 0.6 | -0.738166423 | 0.000262128 | 0.015105783 | yes | down |
| ENSRNOG00000057623 | Copb1 | 0.658 | -0.602840245 | 0.00026755 | 0.015339997 | yes | down |
| ENSRNOG00000016695 | Mmp2 | 1.99 | 0.99261976 | 0.000270015 | 0.015403112 | yes | up |
| ENSRNOG00000005890 | Serbp1 | 0.593 | -0.75448557 | 0.000273283 | 0.015461904 | yes | down |
| ENSRNOG00000060914 | Nudcd2 | 0.523 | -0.935678539 | 0.00027439 | 0.015461904 | yes | down |
| ENSRNOG00000002616 | Slc5a10 | 0.115 | -3.117350349 | 0.00027656 | 0.015461904 | yes | down |
| ENSRNOG00000006082 | Rmdn2 | 0.534 | -0.905564868 | 0.000277922 | 0.015461904 | yes | down |
| ENSRNOG00000013720 | Aebp1 | 1.842 | 0.881410698 | 0.000280492 | 0.015461904 | yes | up |
| ENSRNOG00000007859 | Cep83 | 0.395 | -1.338552219 | 0.000281211 | 0.015461904 | yes | down |
| ENSRNOG00000011976 | Nudt7 | 0.573 | -0.80332603 | 0.000281691 | 0.015461904 | yes | down |
| ENSRNOG00000009857 | Ftsj3 | 0.617 | -0.696978676 | 0.000281997 | 0.015461904 | yes | down |
| ENSRNOG00000018969 | Gpatch4 | 0.444 | -1.172381103 | 0.000283906 | 0.015491409 | yes | down |
| ENSRNOG00000014532 | Lbp | 1.87 | 0.903339295 | 0.000288204 | 0.015604018 | yes | up |
| ENSRNOG00000004301 | Dcaf13 | 0.509 | -0.974667549 | 0.000290813 | 0.015641566 | yes | down |
| ENSRNOG00000059408 | Prr12 | 1.781 | 0.833058296 | 0.000292841 | 0.015676 | yes | up |
| ENSRNOG00000024372 | Cwf19l2 | 0.451 | -1.147466627 | 0.000294351 | 0.015682525 | yes | down |
| ENSRNOG00000007315 | Thoc2 | 0.491 | -1.025916209 | 0.000299669 | 0.015890905 | yes | down |
| ENSRNOG00000001229 | Col18a1 | 1.802 | 0.849981945 | 0.000305656 | 0.016132664 | yes | up |
| ENSRNOG00000003310 | Tmem63a | 1.68 | 0.748450217 | 0.000309991 | 0.016285338 | yes | up |
| ENSRNOG00000016459 | Eif3j | 0.545 | -0.87485975 | 0.000319783 | 0.016721995 | yes | down |
| ENSRNOG00000050636 | Arhgef10l | 1.557 | 0.638946185 | 0.00032191 | 0.016755631 | yes | up |
| ENSRNOG00000061058 | Csde1 | 0.557 | -0.843870243 | 0.000326293 | 0.016860252 | yes | down |
| ENSRNOG00000006227 | Ifih1 | 0.466 | -1.100500104 | 0.000327152 | 0.016860252 | yes | down |
| ENSRNOG00000020440 | Fads2 | 2.49 | 1.316029379 | 0.000329201 | 0.016860252 | yes | up |
| ENSRNOG00000004777 | Esf1 | 0.364 | -1.456404727 | 0.000329891 | 0.016860252 | yes | down |
| ENSRNOG00000005087 | Cebpz | 0.479 | -1.060766771 | 0.000334464 | 0.017017003 | yes | down |
| ENSRNOG00000009156 | Tra2a | 0.546 | -0.873317862 | 0.000338967 | 0.017104585 | yes | down |
| ENSRNOG00000028185 | Tstd3 | 0.424 | -1.238646583 | 0.000339214 | 0.017104585 | yes | down |
| ENSRNOG00000007998 | Ssb | 0.466 | -1.101051259 | 0.00034511 | 0.017324498 | yes | down |
| ENSRNOG00000011781 | Oplah | 1.705 | 0.769894803 | 0.000348223 | 0.017403422 | yes | up |
| ENSRNOG00000013791 | Enpp3 | 1.604 | 0.681640375 | 0.00035183 | 0.017441791 | yes | up |
| ENSRNOG00000026965 | Tmem140 | 1.623 | 0.698504705 | 0.000352079 | 0.017441791 | yes | up |
| ENSRNOG00000049385 | Adamtsl4 | 1.835 | 0.875483698 | 0.000354571 | 0.017466977 | yes | up |
| ENSRNOG00000020038 | Chpf | 2.044 | 1.031212025 | 0.00035568 | 0.017466977 | yes | up |
| ENSRNOG00000003822 | Slu7 | 0.611 | -0.70992593 | 0.000361157 | 0.017510847 | yes | down |
| ENSRNOG00000020918 | Ccnd1 | 2.043 | 1.030437868 | 0.000362655 | 0.017510847 | yes | up |
| ENSRNOG00000013439 | Cpn1 | 1.634 | 0.708490926 | 0.000362766 | 0.017510847 | yes | up |
| ENSRNOG00000028382 | Rfxapl1 | 295.978 | 8.209344562 | 0.000362775 | 0.017510847 | yes | up |
| ENSRNOG00000007410 | Dab1 | 3.292 | 1.718989711 | 0.000370323 | 0.017788602 | yes | up |
| ENSRNOG00000047106 | Mtrf1 | 0.533 | -0.907311768 | 0.000373341 | 0.017788602 | yes | down |
| ENSRNOG00000004481 | Adss | 0.604 | -0.726254489 | 0.00037346 | 0.017788602 | yes | down |
| ENSRNOG00000022745 | RGD1306502 | 0.629 | -0.669760521 | 0.000375106 | 0.017788602 | yes | down |
| ENSRNOG00000002695 | Tfb2m | 0.572 | -0.807017153 | 0.000376403 | 0.017788602 | yes | down |
| ENSRNOG00000001598 | Usp16 | 0.515 | -0.957181155 | 0.000384013 | 0.0179898 | yes | down |
| ENSRNOG00000016099 | Id4 | 1.815 | 0.859749968 | 0.000384304 | 0.0179898 | yes | up |
| ENSRNOG00000048193 | Hnrnph3 | 0.573 | -0.802296735 | 0.000385881 | 0.0179898 | yes | down |
| ENSRNOG00000011261 | Ttc14 | 0.407 | -1.297749143 | 0.000387032 | 0.0179898 | yes | down |
| ENSRNOG00000014674 | Exosc9 | 0.567 | -0.819154914 | 0.000393381 | 0.018209981 | yes | down |
| ENSRNOG00000012651 | Cwc22 | 0.534 | -0.905343041 | 0.000401275 | 0.018499589 | yes | down |
| ENSRNOG00000001770 | Ehhadh | 2.038 | 1.026812209 | 0.000406414 | 0.018660367 | yes | up |
| ENSRNOG00000003291 | Creg1 | 0.425 | -1.234545609 | 0.000412819 | 0.018877705 | yes | down |
| ENSRNOG00000013078 | Zcchc7 | 0.502 | -0.993201014 | 0.000417362 | 0.019008475 | yes | down |
| ENSRNOG00000000925 | Psph | 0.548 | -0.867668707 | 0.000421342 | 0.019070251 | yes | down |
| ENSRNOG00000011944 | Snx10 | 0.308 | -1.697788599 | 0.000422095 | 0.019070251 | yes | down |
| ENSRNOG00000012692 | Riok2 | 0.499 | -1.00160166 | 0.000424788 | 0.019115447 | yes | down |
| ENSRNOG00000011937 | Sgtb | 0.396 | -1.337242176 | 0.00042673 | 0.019126633 | yes | down |
| ENSRNOG00000018005 | Duoxa1 | 2.694 | 1.429966536 | 0.000429935 | 0.019194149 | yes | up |
| ENSRNOG00000014613 | Ddah1 | 1.902 | 0.927883085 | 0.000437688 | 0.019463312 | yes | up |
| ENSRNOG00000000903 | Asl | 1.7 | 0.765117442 | 0.000439862 | 0.019483289 | yes | up |
| ENSRNOG00000056128 | AABR07064719.2 | 0.485 | -1.04361726 | 0.00044829 | 0.019779029 | yes | down |
| ENSRNOG00000046955 | Cbx6 | 1.95 | 0.963303806 | 0.000451748 | 0.019804029 | yes | up |
| ENSRNOG00000018657 | Scrn3 | 0.618 | -0.694506279 | 0.000457196 | 0.019938321 | yes | down |
| ENSRNOG00000048061 | Brcc3 | 0.516 | -0.953392543 | 0.000467801 | 0.020290471 | yes | down |
| ENSRNOG00000012953 | Arsa | 1.501 | 0.585805209 | 0.00047644 | 0.020477044 | yes | up |
| ENSRNOG00000009990 | Zranb2 | 0.617 | -0.695762957 | 0.000477197 | 0.020477044 | yes | down |
| ENSRNOG00000001783 | Tra2b | 0.619 | -0.691301725 | 0.000480046 | 0.020477044 | yes | down |
| ENSRNOG00000010137 | Xpo4 | 0.51 | -0.970405499 | 0.000480426 | 0.020477044 | yes | down |
| ENSRNOG00000023923 | Fastkd2 | 0.569 | -0.812552324 | 0.000489877 | 0.02072278 | yes | down |
| ENSRNOG00000012360 | Gtf2h1 | 0.639 | -0.646199627 | 0.000491696 | 0.02072278 | yes | down |
| ENSRNOG00000011367 | Cyp2r1 | 0.501 | -0.998485586 | 0.000495382 | 0.020800506 | yes | down |
| ENSRNOG00000019501 | Rmnd1 | 0.665 | -0.588760632 | 0.000501062 | 0.020849599 | yes | down |
| ENSRNOG00000009195 | Tnks1bp1 | 1.59 | 0.66874145 | 0.000503118 | 0.020849599 | yes | up |
| ENSRNOG00000049244 | Sepsecs | 0.576 | -0.796911091 | 0.000503197 | 0.020849599 | yes | down |
| ENSRNOG00000018336 | Eps8l2 | 1.526 | 0.60965993 | 0.000504506 | 0.020849599 | yes | up |
| ENSRNOG00000017466 | Kif5b | 0.551 | -0.858981439 | 0.00050687 | 0.020849599 | yes | down |
| ENSRNOG00000017766 | Ca12 | 0.153 | -2.7112698 | 0.000507626 | 0.020849599 | yes | down |
| ENSRNOG00000022597 | Cenpj | 0.542 | -0.882360534 | 0.000509784 | 0.020862337 | yes | down |
| ENSRNOG00000021872 | Zfp213 | 1.537 | 0.620365579 | 0.000516645 | 0.021039006 | yes | up |
| ENSRNOG00000022576 | Nudt12 | 0.633 | -0.660278457 | 0.000520902 | 0.021039006 | yes | down |
| ENSRNOG00000004141 | Vasn | 2.078 | 1.055277629 | 0.000521551 | 0.021039006 | yes | up |
| ENSRNOG00000006990 | Grb7 | 1.697 | 0.762700614 | 0.000523701 | 0.021050538 | yes | up |
| ENSRNOG00000013719 | Dph5 | 0.528 | -0.921817972 | 0.000527145 | 0.021051215 | yes | down |
| ENSRNOG00000060949 | Anxa8 | 0.204 | -2.290388389 | 0.000527445 | 0.021051215 | yes | down |
| ENSRNOG00000015029 | Dbt | 0.61 | -0.713353324 | 0.000531747 | 0.02108546 | yes | down |
| ENSRNOG00000020782 | Pspc1 | 0.439 | -1.188262786 | 0.000532037 | 0.02108546 | yes | down |
| ENSRNOG00000014852 | Fbxo30 | 0.437 | -1.193463955 | 0.000535467 | 0.021147194 | yes | down |
| ENSRNOG00000012406 | Pcbp4 | 1.804 | 0.851114536 | 0.00054085 | 0.021285351 | yes | up |
| ENSRNOG00000009715 | Me1 | 0.416 | -1.26402304 | 0.000558789 | 0.021914997 | yes | down |
| ENSRNOG00000048753 | AABR07008350.1 | 0.381 | -1.393164099 | 0.000565582 | 0.022104672 | yes | down |
| ENSRNOG00000010947 | Mmp14 | 1.639 | 0.712438418 | 0.000582418 | 0.022612093 | yes | up |
| ENSRNOG00000010737 | Mbnl2 | 0.523 | -0.934654604 | 0.000592906 | 0.022934492 | yes | down |
| ENSRNOG00000018333 | Rbm6 | 0.608 | -0.717012193 | 0.000595889 | 0.022971229 | yes | down |
| ENSRNOG00000019743 | Tmem63b | 1.625 | 0.700056985 | 0.000603588 | 0.023188863 | yes | up |
| ENSRNOG00000061031 | Fzd8 | 1.674 | 0.743254636 | 0.000612589 | 0.023226962 | yes | up |
| ENSRNOG00000059244 | LOC100909849 | 1.674 | 0.743254636 | 0.000612589 | 0.023226962 | yes | up |
| ENSRNOG00000000891 |  | 0.509 | -0.972984307 | 0.000613889 | 0.023226962 | yes | down |
| ENSRNOG00000038085 | Rpe | 0.56 | -0.837195 | 0.000614862 | 0.023226962 | yes | down |
| ENSRNOG00000014320 | Inhba | 0.45 | -1.151793345 | 0.000624528 | 0.02338932 | yes | down |
| ENSRNOG00000032885 | LOC100363502 | 0.382 | -1.387400877 | 0.000625439 | 0.02338932 | yes | down |
| ENSRNOG00000012734 | Dcun1d1 | 0.543 | -0.88214784 | 0.000625734 | 0.02338932 | yes | down |
| ENSRNOG00000033101 | Myo18a | 1.639 | 0.713035019 | 0.000627443 | 0.02338932 | yes | up |
| ENSRNOG00000030628 | Eif4a1 | 0.624 | -0.679868236 | 0.000634439 | 0.02357233 | yes | down |
| ENSRNOG00000024482 | Tnrc18 | 1.743 | 0.801305682 | 0.00064408 | 0.023852079 | yes | up |
| ENSRNOG00000014233 | Krt19 | 2.367 | 1.243166277 | 0.000651603 | 0.024051801 | yes | up |
| ENSRNOG00000025025 | Dnttip2 | 0.471 | -1.084754227 | 0.000663178 | 0.024399338 | yes | down |
| ENSRNOG00000017318 | Zfp322a | 0.417 | -1.262301674 | 0.000670072 | 0.024423072 | yes | down |
| ENSRNOG00000004864 | Prpf40a | 0.492 | -1.024169879 | 0.000670278 | 0.024423072 | yes | down |
| ENSRNOG00000017052 | Pianp | 2.304 | 1.204186962 | 0.00067031 | 0.024423072 | yes | up |
| ENSRNOG00000011040 | AABR07063855.1 | 0.499 | -1.001982469 | 0.000676524 | 0.024561397 | yes | down |
| ENSRNOG00000058050 | Bclaf1 | 0.608 | -0.718006306 | 0.000678456 | 0.024561397 | yes | down |
| ENSRNOG00000019175 | Ercc6l2 | 0.567 | -0.818179747 | 0.000691021 | 0.024845244 | yes | down |
| ENSRNOG00000007884 | Bcap29 | 0.524 | -0.931227432 | 0.000692562 | 0.024845244 | yes | down |
| ENSRNOG00000012574 | Pum3 | 0.579 | -0.788475672 | 0.000701058 | 0.025009868 | yes | down |
| ENSRNOG00000032414 | Tmem161b | 0.575 | -0.797438493 | 0.000701915 | 0.025009868 | yes | down |
| ENSRNOG00000013003 | Srms | 2.995 | 1.582371594 | 0.000736108 | 0.025942018 | yes | up |
| ENSRNOG00000000823 | Gcc2 | 0.558 | -0.842667017 | 0.00073645 | 0.025942018 | yes | down |
| ENSRNOG00000000796 | Ranbp2 | 0.495 | -1.013281158 | 0.000737263 | 0.025942018 | yes | down |
| ENSRNOG00000042308 | LOC680200 | 0.421 | -1.248898581 | 0.000740067 | 0.02595981 | yes | down |
| ENSRNOG00000004130 | Anapc4 | 0.637 | -0.649965174 | 0.000742592 | 0.025967729 | yes | down |
| ENSRNOG00000042163 | Btbd19 | 0.474 | -1.075781903 | 0.000758646 | 0.026365873 | yes | down |
| ENSRNOG00000010977 | Igfbp6 | 4.044 | 2.015718037 | 0.000771229 | 0.026720942 | yes | up |
| ENSRNOG00000002642 | Ptges3 | 0.561 | -0.834845648 | 0.000779085 | 0.026828564 | yes | down |
| ENSRNOG00000028616 | Pck1 | 2.946 | 1.558969911 | 0.000785061 | 0.026952181 | yes | up |
| ENSRNOG00000001254 | Col6a2 | 2.015 | 1.010662171 | 0.000790998 | 0.027073691 | yes | up |
| ENSRNOG00000002789 | AABR07039648.1 | 0.454 | -1.138869178 | 0.000794209 | 0.027101466 | yes | down |
| ENSRNOG00000015142 | Timm21 | 0.493 | -1.021347919 | 0.000808738 | 0.027325233 | yes | down |
| ENSRNOG00000047322 | Crb3 | 1.638 | 0.711718849 | 0.000809031 | 0.027325233 | yes | up |
| ENSRNOG00000015267 | LOC108348083 | 0.002 | -9.133272046 | 0.000812674 | 0.027325233 | yes | down |
| ENSRNOG00000022325 | Smc2 | 0.446 | -1.164055052 | 0.000819436 | 0.027325233 | yes | down |
| ENSRNOG00000011278 | Stk3 | 0.577 | -0.793119713 | 0.000821078 | 0.027325233 | yes | down |
| ENSRNOG00000015880 | Dpep1 | 2.034 | 1.024090503 | 0.000822163 | 0.027325233 | yes | up |
| ENSRNOG00000020903 | Lto1 | 1.67 | 0.739659034 | 0.000823374 | 0.027325233 | yes | up |
| ENSRNOG00000051756 | Zfp62 | 0.365 | -1.452312697 | 0.000824159 | 0.027325233 | yes | down |
| ENSRNOG00000010452 | Cycs | 0.49 | -1.02978973 | 0.000825047 | 0.027325233 | yes | down |
| ENSRNOG00000009460 | Pdzd8 | 0.492 | -1.022014589 | 0.000827378 | 0.027325233 | yes | down |
| ENSRNOG00000003901 | Cfap36 | 0.463 | -1.109671072 | 0.000835545 | 0.027514521 | yes | down |
| ENSRNOG00000046996 | Pea15 | 1.537 | 0.620534465 | 0.000844291 | 0.027585418 | yes | up |
| ENSRNOG00000000981 | Scarb1 | 1.611 | 0.687658292 | 0.000846008 | 0.027585418 | yes | up |
| ENSRNOG00000012087 | Ufsp2 | 0.565 | -0.823466564 | 0.000846769 | 0.027585418 | yes | down |
| ENSRNOG00000059852 | Rn5-8s | 2.394 | 1.259122374 | 0.000847467 | 0.027585418 | yes | up |
| ENSRNOG00000019721 | Ypel3 | 1.892 | 0.919650425 | 0.000851935 | 0.027651164 | yes | up |
| ENSRNOG00000034013 | Acaca | 0.338 | -1.563468819 | 0.000860266 | 0.027841551 | yes | down |
| ENSRNOG00000045740 | Tmx3 | 0.401 | -1.316951333 | 0.000872739 | 0.028029226 | yes | down |
| ENSRNOG00000001642 | Tbc1d23 | 0.664 | -0.591703598 | 0.000876063 | 0.028029226 | yes | down |
| ENSRNOG00000020698 | Rnd2 | 1.937 | 0.954152649 | 0.000876264 | 0.028029226 | yes | up |
| ENSRNOG00000005256 | Zc3h15 | 0.574 | -0.80193298 | 0.000879134 | 0.028029226 | yes | down |
| ENSRNOG00000004500 | Myc | 0.363 | -1.46183007 | 0.000880954 | 0.028029226 | yes | down |
| ENSRNOG00000015686 | Cert1 | 0.566 | -0.821053211 | 0.000887915 | 0.028123785 | yes | down |
| ENSRNOG00000001205 | Agpat3 | 1.85 | 0.887518278 | 0.000889967 | 0.028123785 | yes | up |
| ENSRNOG00000017191 | Trim5 | 0.649 | -0.6231396 | 0.000891396 | 0.028123785 | yes | down |
| ENSRNOG00000050932 | Pfdn4 | 0.583 | -0.777627902 | 0.000910366 | 0.028642304 | yes | down |
| ENSRNOG00000004512 | Apool | 0.51 | -0.970396995 | 0.000913008 | 0.028645641 | yes | down |
| ENSRNOG00000002089 | Ccng2 | 1.675 | 0.743986148 | 0.000920988 | 0.028693679 | yes | up |
| ENSRNOG00000011561 | Nln | 0.604 | -0.727235724 | 0.000921287 | 0.028693679 | yes | down |
| ENSRNOG00000015848 | Etfrf1 | 0.565 | -0.822549671 | 0.000922161 | 0.028693679 | yes | down |
| ENSRNOG00000019848 | Rbm39 | 0.604 | -0.72728128 | 0.000929683 | 0.028848259 | yes | down |
| ENSRNOG00000019473 | NEWGENE_1582994 | 0.601 | -0.734990949 | 0.000949902 | 0.029314598 | yes | down |
| ENSRNOG00000011541 | Cygb | 1.938 | 0.954856602 | 0.000981987 | 0.030076027 | yes | up |
| ENSRNOG00000025145 | Rmdn1 | 0.554 | -0.851788299 | 0.000982042 | 0.030076027 | yes | down |
| ENSRNOG00000020990 | Fgf21 | 0.261 | -1.940362573 | 0.000982563 | 0.030076027 | yes | down |
| ENSRNOG00000006335 | Klhl9 | 0.583 | -0.778429787 | 0.000989766 | 0.030134977 | yes | down |
| ENSRNOG00000010468 | Elovl6 | 0.206 | -2.2800922 | 0.000989825 | 0.030134977 | yes | down |
| ENSRNOG00000012775 | Ccdc127 | 0.55 | -0.861341667 | 0.000997964 | 0.030183165 | yes | down |
| ENSRNOG00000004161 | Mtif2 | 0.509 | -0.974662061 | 0.000998085 | 0.030183165 | yes | down |
| ENSRNOG00000030285 | Epha3 | 0.435 | -1.201525506 | 0.001005544 | 0.030183165 | yes | down |
| ENSRNOG00000004521 | Prpf39 | 0.411 | -1.281236895 | 0.001008247 | 0.030183165 | yes | down |
| ENSRNOG00000011647 | S100a6 | 2.822 | 1.496573321 | 0.001009481 | 0.030183165 | yes | up |
| ENSRNOG00000001469 | Eln | 1.724 | 0.785762351 | 0.001011217 | 0.030183165 | yes | up |
| ENSRNOG00000009963 | Ctps1 | 0.452 | -1.144161669 | 0.001013503 | 0.030183165 | yes | down |
| ENSRNOG00000018937 | Gstm7 | 1.651 | 0.723242251 | 0.001015458 | 0.030183165 | yes | up |
| ENSRNOG00000042620 | Marveld1 | 0.293 | -1.769394976 | 0.001018639 | 0.030185423 | yes | down |
| ENSRNOG00000003599 | Utp3 | 0.607 | -0.721167314 | 0.001020879 | 0.030185423 | yes | down |
| ENSRNOG00000002473 | Odr4 | 0.558 | -0.840639993 | 0.001036591 | 0.030552521 | yes | down |
| ENSRNOG00000004844 | Med30 | 0.559 | -0.840248622 | 0.001038705 | 0.030552521 | yes | down |
| ENSRNOG00000001383 | Slc8b1 | 1.78 | 0.831764266 | 0.001054285 | 0.030930252 | yes | up |
| ENSRNOG00000000204 | Syncrip | 0.613 | -0.706297907 | 0.001057436 | 0.030942322 | yes | down |
| ENSRNOG00000039017 | Naa50 | 0.546 | -0.874249578 | 0.001070269 | 0.031236917 | yes | down |
| ENSRNOG00000029922 | Tpk1 | 0.651 | -0.618200622 | 0.001085068 | 0.031587226 | yes | down |
| ENSRNOG00000019892 | Lrrfip1 | 0.536 | -0.898518551 | 0.00108985 | 0.031596986 | yes | down |
| ENSRNOG00000006364 | Dld | 0.599 | -0.738513042 | 0.001092806 | 0.031596986 | yes | down |
| ENSRNOG00000019228 | Pik3r2 | 1.514 | 0.597952703 | 0.001093796 | 0.031596986 | yes | up |
| ENSRNOG00000003931 | Arsg | 1.583 | 0.66292733 | 0.001099443 | 0.031679097 | yes | up |
| ENSRNOG00000053247 | Snx21 | 1.637 | 0.710655461 | 0.00111951 | 0.032175237 | yes | up |
| ENSRNOG00000021102 | Scn1b | 2.618 | 1.38826846 | 0.00112671 | 0.032299966 | yes | up |
| ENSRNOG00000050854 | LOC108348101 | 0.105 | -3.257132918 | 0.001134768 | 0.032448615 | yes | down |
| ENSRNOG00000001829 | Ube2v2 | 0.437 | -1.195421595 | 0.001143111 | 0.03260464 | yes | down |
| ENSRNOG00000007088 | Xpot | 0.563 | -0.82917977 | 0.001164218 | 0.033123035 | yes | down |
| ENSRNOG00000010186 | Cdv3 | 0.623 | -0.683117671 | 0.001167653 | 0.033135824 | yes | down |
| ENSRNOG00000011586 | LOC100909712 | 0.488 | -1.03547533 | 0.001170535 | 0.033135824 | yes | down |
| ENSRNOG00000019374 | Csk | 1.545 | 0.62777176 | 0.001174955 | 0.033177802 | yes | up |
| ENSRNOG00000024336 | Senp6 | 0.603 | -0.729624522 | 0.00118009 | 0.033239681 | yes | down |
| ENSRNOG00000016085 | Mpzl2 | 0.432 | -1.212503475 | 0.001183921 | 0.033264645 | yes | down |
| ENSRNOG00000003350 | Mospd2 | 0.658 | -0.60469976 | 0.001189363 | 0.033334633 | yes | down |
| ENSRNOG00000018662 | Amacr | 1.815 | 0.860098543 | 0.001210638 | 0.033846909 | yes | up |
| ENSRNOG00000009994 | Dlat | 0.509 | -0.975315586 | 0.001214501 | 0.033871074 | yes | down |
| ENSRNOG00000053803 | AABR07058656.1 | 3.419 | 1.773615525 | 0.001240513 | 0.034511326 | yes | up |
| ENSRNOG00000036592 | Zfp518a | 0.446 | -1.163873252 | 0.00124374 | 0.034516067 | yes | down |
| ENSRNOG00000002529 | Rap2c | 0.578 | -0.789650336 | 0.001250915 | 0.034630102 | yes | down |
| ENSRNOG00000010350 | Rcan2 | 2.272 | 1.183735721 | 0.001258555 | 0.034756418 | yes | up |
| ENSRNOG00000054890 | Flna | 1.771 | 0.824880263 | 0.001267561 | 0.034842565 | yes | up |
| ENSRNOG00000011555 | Lrrc40 | 0.665 | -0.588292578 | 0.001267844 | 0.034842565 | yes | down |
| ENSRNOG00000050172 | Thpol1 | 2.285 | 1.191880888 | 0.0012819 | 0.035143337 | yes | up |
| ENSRNOG00000029078 | Hmgn5b | 0.384 | -1.380991519 | 0.001333136 | 0.036336861 | yes | down |
| ENSRNOG00000003508 | Ergic1 | 0.6 | -0.736328234 | 0.001337706 | 0.036336861 | yes | down |
| ENSRNOG00000058842 | Sptbn2 | 1.804 | 0.851561932 | 0.001339612 | 0.036336861 | yes | up |
| ENSRNOG00000056907 | Nipbl | 0.494 | -1.018459724 | 0.001341758 | 0.036336861 | yes | down |
| ENSRNOG00000021780 | Rad51d | 1.862 | 0.896520103 | 0.001353414 | 0.036375416 | yes | up |
| ENSRNOG00000005904 | Cdc27 | 0.55 | -0.861418352 | 0.001356489 | 0.036375416 | yes | down |
| ENSRNOG00000053201 | Gpcpd1 | 0.462 | -1.115011811 | 0.001359046 | 0.036375416 | yes | down |
| ENSRNOG00000006642 | Yipf6 | 0.606 | -0.722156632 | 0.001362584 | 0.036383895 | yes | down |
| ENSRNOG00000024647 | Ppp4r2 | 0.621 | -0.687361147 | 0.00137218 | 0.036402906 | yes | down |
| ENSRNOG00000010150 | Strbp | 0.594 | -0.750913565 | 0.0013759 | 0.036402906 | yes | down |
| ENSRNOG00000021438 | Tuba1c | 0.534 | -0.904181675 | 0.001376463 | 0.036402906 | yes | down |
| ENSRNOG00000036682 | Pycr1 | 0.378 | -1.403290782 | 0.001379411 | 0.036402906 | yes | down |
| ENSRNOG00000032735 | Srek1 | 0.58 | -0.784646821 | 0.001383589 | 0.036428054 | yes | down |
| ENSRNOG00000004477 | Ublcp1 | 0.65 | -0.622030665 | 0.001397287 | 0.036685827 | yes | down |
| ENSRNOG00000014610 | Anpep | 1.798 | 0.84650447 | 0.001403123 | 0.036685827 | yes | up |
| ENSRNOG00000012654 | Vps36 | 0.6 | -0.736952172 | 0.001410482 | 0.036748774 | yes | down |
| ENSRNOG00000043201 | Coq8a | 1.584 | 0.663973206 | 0.001413979 | 0.036748774 | yes | up |
| ENSRNOG00000010260 | Dixdc1 | 0.496 | -1.010205602 | 0.001415291 | 0.036748774 | yes | down |
| ENSRNOG00000007719 | Ccnc | 0.57 | -0.809839485 | 0.001440761 | 0.037238882 | yes | down |
| ENSRNOG00000050539 | Fbln5 | 2.108 | 1.075869643 | 0.001445192 | 0.037268149 | yes | up |
| ENSRNOG00000005067 | Zfp36l2 | 2.178 | 1.122915081 | 0.001467234 | 0.037750361 | yes | up |
| ENSRNOG00000007098 | Gtpbp10 | 0.483 | -1.051177674 | 0.001476807 | 0.037910317 | yes | down |
| ENSRNOG00000003228 | Mid1ip1 | 0.66 | -0.600373754 | 0.001484864 | 0.038028859 | yes | down |
| ENSRNOG00000013493 | Psma4 | 0.531 | -0.913467589 | 0.001490715 | 0.038028859 | yes | down |
| ENSRNOG00000016541 | Enc1 | 1.787 | 0.837668507 | 0.001505606 | 0.038215316 | yes | up |
| ENSRNOG00000032293 | Polg | 1.927 | 0.946198032 | 0.00150953 | 0.038229004 | yes | up |
| ENSRNOG00000016105 | Xrcc5 | 0.608 | -0.717937725 | 0.001520876 | 0.0384302 | yes | down |
| ENSRNOG00000010890 | Bmp1 | 1.673 | 0.742331739 | 0.001528629 | 0.038539882 | yes | up |
| ENSRNOG00000032152 | Rab11fip3 | 1.656 | 0.727400565 | 0.001536624 | 0.038569252 | yes | up |
| ENSRNOG00000005849 | Aco1 | 1.669 | 0.738811193 | 0.001550182 | 0.038823287 | yes | up |
| ENSRNOG00000005982 | C1d | 0.615 | -0.701269182 | 0.001567926 | 0.039180798 | yes | down |
| ENSRNOG00000011078 | LOC100912604 | 0.281 | -1.830740752 | 0.001586086 | 0.039309969 | yes | down |
| ENSRNOG00000012876 | Slc6a13 | 2.086 | 1.060901236 | 0.001587016 | 0.039309969 | yes | up |
| ENSRNOG00000053991 | P3h1 | 1.5 | 0.585360952 | 0.00159754 | 0.039484069 | yes | up |
| ENSRNOG00000026913 | Bbs10 | 0.497 | -1.010025888 | 0.001612094 | 0.039756766 | yes | down |
| ENSRNOG00000017612 | Vps35 | 0.652 | -0.616114434 | 0.001628613 | 0.040076664 | yes | down |
| ENSRNOG00000024136 | Fam151b | 0.523 | -0.935294488 | 0.001652065 | 0.040537005 | yes | down |
| ENSRNOG00000014750 | Tasor | 0.506 | -0.981697659 | 0.001654498 | 0.040537005 | yes | down |
| ENSRNOG00000020770 | Arl4d | 0.312 | -1.680372662 | 0.001659926 | 0.040581967 | yes | down |
| ENSRNOG00000012580 | Ccdc141 | 0.398 | -1.327570097 | 0.001670269 | 0.040746618 | yes | down |
| ENSRNOG00000026316 | Wdr43 | 0.496 | -1.010771013 | 0.001687006 | 0.041066235 | yes | down |
| ENSRNOG00000058081 | AABR07069733.1 | 0.332 | -1.590205348 | 0.001701484 | 0.041329605 | yes | down |
| ENSRNOG00000012500 | Ddx10 | 0.541 | -0.886333513 | 0.001725428 | 0.041540979 | yes | down |
| ENSRNOG00000007650 | Cd63 | 1.949 | 0.962586016 | 0.001726113 | 0.041540979 | yes | up |
| ENSRNOG00000013653 | Pdlim7 | 1.83 | 0.872010055 | 0.001732253 | 0.041540979 | yes | up |
| ENSRNOG00000051606 | LOC100360380 | 0.369 | -1.440128286 | 0.001748659 | 0.041783727 | yes | down |
| ENSRNOG00000046984 | St6galnac6 | 1.515 | 0.599069303 | 0.001749775 | 0.041783727 | yes | up |
| ENSRNOG00000009310 | Nmd3 | 0.589 | -0.763390214 | 0.001757578 | 0.041881531 | yes | down |
| ENSRNOG00000018011 | Tasor2 | 0.47 | -1.088729417 | 0.001764404 | 0.041955663 | yes | down |
| ENSRNOG00000059956 | Bcl6b | 0.663 | -0.593677564 | 0.001785682 | 0.042283606 | yes | down |
| ENSRNOG00000003256 | Ccng1 | 0.575 | -0.797313485 | 0.001811751 | 0.042811143 | yes | down |
| ENSRNOG00000007548 | Polr3f | 0.614 | -0.704845038 | 0.001827761 | 0.043009795 | yes | down |
| ENSRNOG00000060979 | Hspa13 | 0.611 | -0.710688495 | 0.001827773 | 0.043009795 | yes | down |
| ENSRNOG00000020995 | Fut1 | 0.181 | -2.465943496 | 0.001835624 | 0.043104718 | yes | down |
| ENSRNOG00000016855 | B3galnt2 | 0.637 | -0.650805999 | 0.001858628 | 0.043464181 | yes | down |
| ENSRNOG00000029966 | Xrcc4 | 0.437 | -1.194670111 | 0.001870232 | 0.043645195 | yes | down |
| ENSRNOG00000000082 | Hltf | 0.402 | -1.31647987 | 0.00190231 | 0.04430225 | yes | down |
| ENSRNOG00000051563 | Giot1 | 0.37 | -1.434984764 | 0.001914331 | 0.044490463 | yes | down |
| ENSRNOG00000009565 | Pdk4 | 0.472 | -1.083524603 | 0.001921645 | 0.044568759 | yes | down |
| ENSRNOG00000002832 | Slc16a2 | 1.571 | 0.651839511 | 0.001940346 | 0.044910272 | yes | up |
| ENSRNOG00000007673 | Ppig | 0.499 | -1.004282934 | 0.001971504 | 0.045538107 | yes | down |
| ENSRNOG00000016066 | Bambi | 1.919 | 0.940109113 | 0.001975857 | 0.045545509 | yes | up |
| ENSRNOG00000006185 | Etaa1 | 0.483 | -1.049420196 | 0.001988181 | 0.04573627 | yes | down |
| ENSRNOG00000004035 | Krr1 | 0.523 | -0.934193779 | 0.001993969 | 0.045776185 | yes | down |
| ENSRNOG00000033065 | Rad50 | 0.542 | -0.884453327 | 0.002042613 | 0.046614361 | yes | down |
| ENSRNOG00000000512 | Slc26a8 | 0.565 | -0.823677854 | 0.00204286 | 0.046614361 | yes | down |
| ENSRNOG00000025669 | Tmem104 | 1.664 | 0.734760245 | 0.002057004 | 0.046642639 | yes | up |
| ENSRNOG00000021573 | Dpy19l3 | 1.531 | 0.614555226 | 0.002059096 | 0.046642639 | yes | up |
| ENSRNOG00000000480 | Phf1 | 1.561 | 0.642529038 | 0.002060618 | 0.046642639 | yes | up |
| ENSRNOG00000020480 | Fads1 | 2.33 | 1.220247961 | 0.002072087 | 0.046755509 | yes | up |
| ENSRNOG00000021881 | Metap2 | 0.645 | -0.632788187 | 0.002077325 | 0.046755509 | yes | down |
| ENSRNOG00000006391 | Smarcad1 | 0.527 | -0.923512403 | 0.002079322 | 0.046755509 | yes | down |
| ENSRNOG00000001376 | Mettl7a | 1.881 | 0.911463305 | 0.002085456 | 0.046755509 | yes | up |
| ENSRNOG00000006632 | Rps6ka3 | 0.503 | -0.990732794 | 0.002086302 | 0.046755509 | yes | down |
| ENSRNOG00000046867 | Zfp260 | 0.004 | -8.006036997 | 0.002091276 | 0.046774187 | yes | down |
| ENSRNOG00000008307 | Nanp | 0.578 | -0.791932088 | 0.002097862 | 0.046828761 | yes | down |
| ENSRNOG00000006973 | RGD1307947 | 0.469 | -1.091029544 | 0.002113876 | 0.047001785 | yes | down |
| ENSRNOG00000054011 | Tbc1d8b | 0.589 | -0.764610505 | 0.002113936 | 0.047001785 | yes | down |
| ENSRNOG00000016945 | Pla2g2a | 5.294 | 2.404289336 | 0.002127669 | 0.047214179 | yes | up |
| ENSRNOG00000005775 | Phf14 | 0.54 | -0.887916866 | 0.002141023 | 0.047248636 | yes | down |
| ENSRNOG00000007240 | Rrs1 | 0.465 | -1.10575828 | 0.002155387 | 0.047390237 | yes | down |
| ENSRNOG00000056753 | Hectd2 | 0.503 | -0.991607638 | 0.002156581 | 0.047390237 | yes | down |
| ENSRNOG00000012705 | Pcgf2 | 1.585 | 0.664589568 | 0.002162673 | 0.04741934 | yes | up |
| ENSRNOG00000017069 | Mark4 | 1.63 | 0.704937854 | 0.002168524 | 0.04741934 | yes | up |
| ENSRNOG00000024929 | Nudcd1 | 0.523 | -0.934099124 | 0.002173695 | 0.04741934 | yes | down |
| ENSRNOG00000058039 | Acta2 | 1.939 | 0.955166375 | 0.002174698 | 0.04741934 | yes | up |
| ENSRNOG00000048025 | Cstf2 | 0.568 | -0.81674042 | 0.002179842 | 0.047439909 | yes | down |
| ENSRNOG00000036571 | Ces2c | 1.877 | 0.90860435 | 0.002198696 | 0.047666556 | yes | up |
| ENSRNOG00000033570 | Arhgap8 | 1.671 | 0.741125644 | 0.002213517 | 0.047851859 | yes | up |
| ENSRNOG00000050437 | Bod1l1 | 0.578 | -0.789882006 | 0.002215717 | 0.047851859 | yes | down |
| ENSRNOG00000028348 | Lhx8 | 0.444 | -1.169778643 | 0.002233338 | 0.047978111 | yes | down |
| ENSRNOG00000048109 | LOC100365839 | 0.435 | -1.200773702 | 0.002237589 | 0.047978111 | yes | down |
| ENSRNOG00000002035 | Paqr3 | 0.564 | -0.826757332 | 0.002238554 | 0.047978111 | yes | down |
| ENSRNOG00000008415 | Nab2 | 2.608 | 1.383149792 | 0.00226096 | 0.048366557 | yes | up |
| ENSRNOG00000051256 | R3hcc1l | 0.593 | -0.754673875 | 0.002289533 | 0.048791327 | yes | down |
| ENSRNOG00000048932 | Smagp | 1.567 | 0.647560732 | 0.002293243 | 0.048791327 | yes | up |
| ENSRNOG00000023969 | Herc6 | 0.456 | -1.131576937 | 0.002293776 | 0.048791327 | yes | down |
| ENSRNOG00000005021 | Orc4 | 0.489 | -1.033223974 | 0.002299759 | 0.048826647 | yes | down |
| ENSRNOG00000020989 | Tm7sf2 | 1.867 | 0.900695279 | 0.002310325 | 0.048958959 | yes | up |
| ENSRNOG00000007489 | Zfp41 | 1.729 | 0.790239119 | 0.002326011 | 0.049108741 | yes | up |
| ENSRNOG00000055232 | U6 | 0.439 | -1.188534845 | 0.002326089 | 0.049108741 | yes | down |
| ENSRNOG00000011678 | Wbp4 | 0.618 | -0.693942016 | 0.00233394 | 0.04918256 | yes | down |
| ENSRNOG00000003233 | Serpinf2 | 1.535 | 0.61804891 | 0.002346727 | 0.049359927 | yes | up |
| ENSRNOG00000015734 | Ube3a | 0.538 | -0.893872555 | 0.002356228 | 0.049392224 | yes | down |
| ENSRNOG00000061429 | Rph3al | 1.545 | 0.627477965 | 0.002357008 | 0.049392224 | yes | up |
| ENSRNOG00000037227 | Yes1 | 0.479 | -1.060795272 | 0.002383041 | 0.04984527 | yes | down |

**Table S3|** The detection standard curve for each fatty acid

| **Name** | **Linear equation** | **R^2^** | **Linear range (ug/ml)** |
| --- | --- | --- | --- |
| C4:0 | y = 0.676115 * x + 0.010459 | 0.999579272 | 0.002-50 |
| C6:0 | y = 2.145062 * x + 2.400182E-004 | 0.999673401 | 0.002-50 |
| C8:0 | y = 2.808091 * x + 3.552830E-004 | 0.999690439 | 0.002-50 |
| C10:0 | y = 2.974875 * x + 4.407884E-004 | 0.999709411 | 0.004-100 |
| C11:0 | y = 2.938498 * x + 3.125274E-004 | 0.999767058 | 0.002-50 |
| C12:0 | y = 2.924615 * x + 3.922994E-004 | 0.999828801 | 0.004-100 |
| C13:0 | y = 2.774600 * x + 3.058208E-004 | 0.999713304 | 0.002-50 |
| C14:0 | y = 2.578346 * x + 0.015441 | 0.999618099 | 0.002-50 |
| C14:1N5 | y = 1.093605 * x - 6.418088E-004 | 0.999330953 | 0.002-50 |
| C15:0 | y = 2.534515 * x + 1.383307E-004 | 0.999747865 | 0.002-50 |
| C15:1N5 | y = 0.979738 * x + 1.153024E-004 | 0.999792521 | 0.002-50 |
| C16:0 | y = 2.542880 * x + 0.071060 | 0.999665542 | 0.004-100 |
| C16:1N7 | y = 0.765158 * x + 0.005247 | 0.999702007 | 0.002-50 |
| C17:0 | y = 2.399196 * x - 8.808896E-004 | 0.99969776 | 0.004-100 |
| C17:1N7 | y = 0.746499 * x + 1.371566E-004 | 0.999695696 | 0.002-50 |
| C18:0 | y = 2.435727 * x + 0.084298 | 0.999631009 | 0.004-100 |
| C18:1TN9 | y = 0.775054 * x - 0.004505 | 0.999647376 | 0.002-50 |
| C18:1N9 | y = 0.716432 * x + 0.100592 | 0.999527484 | 0.004-100 |
| C18:2TTN6 | y = 0.964511 * x - 0.020342 | 0.999577512 | 0.002-50 |
| C18:2N6 | y = 0.956557 * x - 0.026392 | 0.999563221 | 0.002-50 |
| C18:3N6 | y = 0.781160 * x + 3.118248E-004 | 0.999855424 | 0.004-100 |
| C18:3N3 | y = 1.046795 * x - 0.004403 | 0.999779978 | 0.002-50 |
| C20:0 | y = 1.870038 * x + 1.526439E-004 | 0.999768281 | 0.004-100 |
| C20:1N9 | y = 0.827732 * x - 0.007419 | 0.999985157 | 0.002-50 |
| C20:2N6 | y = 0.914886 * x - 0.023660 | 0.999542002 | 0.002-50 |
| C21:0 | y = 1.552001 * x + 0.001375 | 0.999867097 | 0.002-50 |
| C20:3N6 | y = 0.878719 * x - 0.029676 | 0.999492438 | 0.002-50 |
| C20:4N6 | y = 0.948662 * x - 0.047859 | 0.999535526 | 0.002-50 |
| C20:3N3 | y = 1.123874 * x - 0.064862 | 0.999479333 | 0.002-50 |
| C22:0 | y = 1.602850 * x - 2.476369E-004 | 0.999544385 | 0.004-100 |
| C20:5N3 | y = 1.080144 * x - 0.010530 | 0.999981046 | 0.002-50 |
| C22:1N9 | y = 0.940492 * x - 0.008849 | 0.999855724 | 0.002-50 |
| C22:2N6 | y = 0.548524 * x + 3.968627E-005 | 0.999440545 | 0.002-50 |
| C23:0 | y = 1.880202 * x - 0.008220 | 0.999889805 | 0.002-50 |
| C22:4N6 | y = 1.009597 * x - 0.025842 | 0.999199063 | 0.002-50 |
| C22:5N6 | y = 1.021545 * x - 0.025451 | 0.999221472 | 0.002-50 |
| C24:0 | y = 1.786569 * x - 0.018456 | 0.999835667 | 0.004-100 |
| C22:5N3 | y = 1.026703 * x - 0.038717 | 0.999941074 | 0.002-50 |
| C24:1N9 | y = 1.041510 * x - 0.048647 | 0.999675 | 0.002-50 |
| C22:6N3 | y = 1.096107 * x - 0.067056 | 0.999531592 | 0.002-50 |


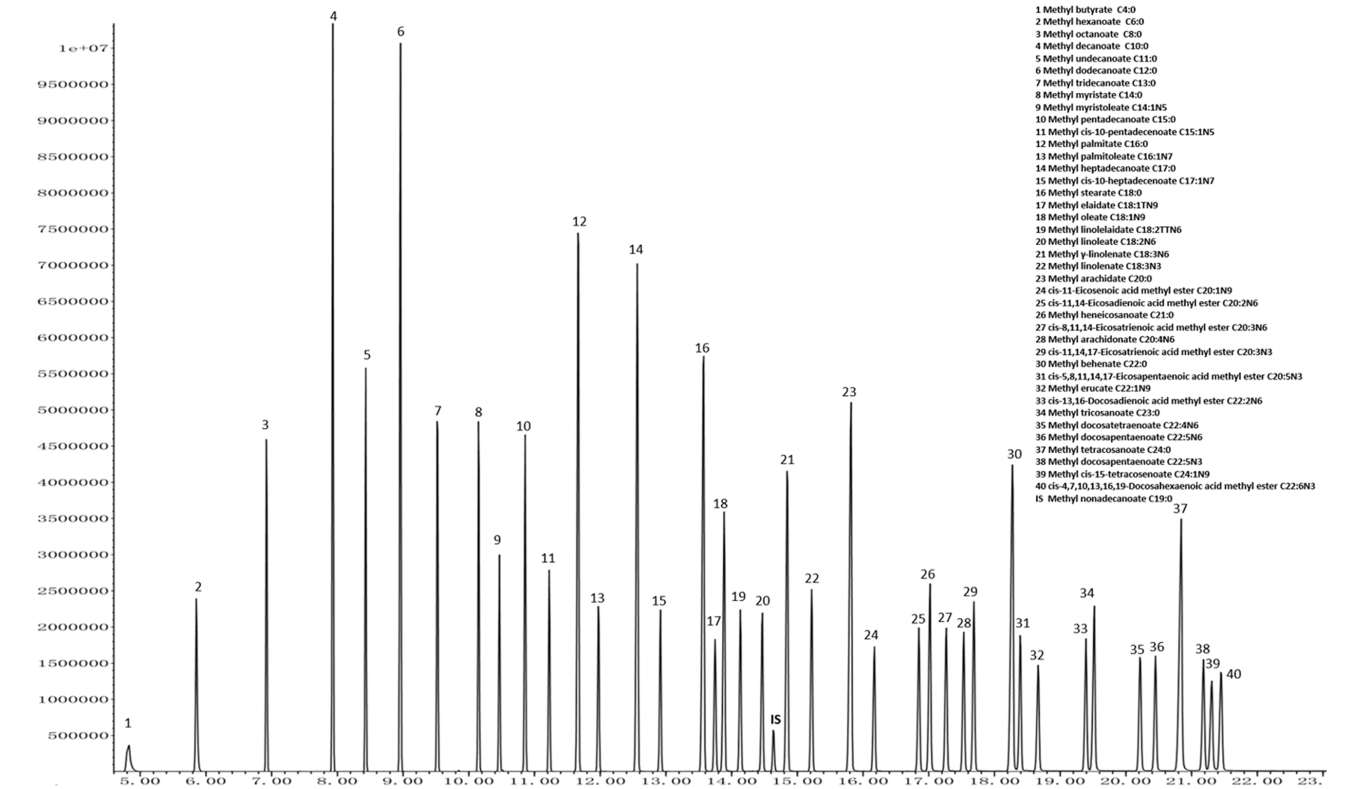


**Figure S1|** The total ions chromatogram (TIC) of hepatic fatty acids





**Figure S2|** General parameters of fructose-induced rats by baicalein treatment. Data were represented as Mean ± SEM (n = 6/group), *^##^p < 0.01*, *^#^p < 0.05*, compared with CTR; ***p < 0.01*, **p < 0.05*, compared to FRU.
